# Is there a gender gap in chemical sciences scholarly communication?†
†Electronic supplementary information (ESI) available: Total numbers, percentages, confidence intervals and significances for figures. See DOI: 10.1039/c9sc04090k


**DOI:** 10.1039/c9sc04090k

**Published:** 2020-01-28

**Authors:** A. E. Day, P. Corbett, J. Boyle

**Affiliations:** a Royal Society of Chemistry , Thomas Graham House (290), Science Park, Milton Road , Cambridge , CB4 0WF , UK . Email: daya@rsc.org

## Abstract

The Royal Society of Chemistry is committed to investigating and addressing the barriers and biases which face women in the chemical sciences. The cornerstone of this is a thorough analysis of data regarding submissions, review and citations for Royal Society of Chemistry journals from January 2014 until July 2018, since the number and impact of publications and citations are an important factor when seeking research funding and for the progression of academic career. We have applied standard statistical techniques to multiple data sources to perform this analysis, and have investigated whether interactions between variables are significant in affecting various outcomes (author gender; reviewer gender; reviewer recommendations and submission outcome) in addition to considering variables individually. By considering several different data sources, we found that a baseline of approximately a third of chemistry researchers are female overall, although this differs considerably with Chemistry sub-discipline. Rather than one dominant bias effect, we observe complex interactions and a gradual trickle-down decrease in this female percentage through the publishing process and each of these female percentages is less than the last: authors of submissions; authors of RSC submissions which are not rejected without peer review; authors of accepted RSC publications; authors of cited articles. The success rate for female authors to progress through each of these publishing stages is lower than that for male authors. There is a decreasing female percentage when progressing through from first authors to corresponding authors to reviewers, reflecting the decreasing female percentage with seniority in Chemistry research observed in the “Diversity landscape of the chemical sciences” report. Highlights and actions from this analysis form the basis of an accompanying report to be released from the Royal Society of Chemistry.

## Abstract 2

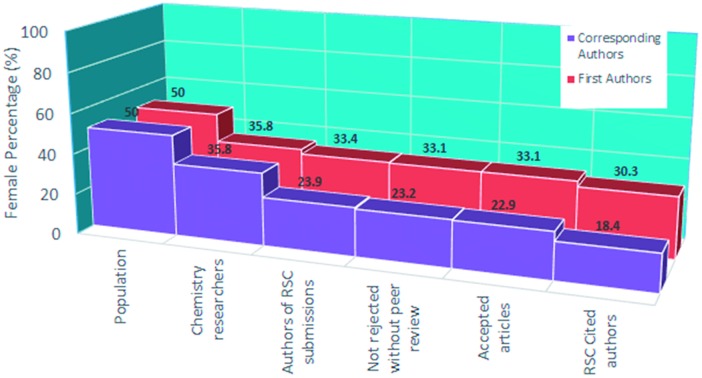
Characterisation of gender differences throughout peer-review publication process as revealed by thorough analysis of Royal Society of Chemistry submissions, publications and citation data.

## Introduction

The “Diversity landscape of the chemical sciences” report,^1^ published in 2018 by the Royal Society of Chemistry, highlighted the importance of diversity to our mission to advance the chemical sciences, and drew together some of the available evidence about its current state through education and career progression. One particular cause for concern highlighted therein was “the leaky pipeline” which clearly demonstrated the drop-off of female researchers with career progression in higher education. The causes of this were explored further in our “Breaking the Barriers” report^2^ along with a five-point action plan to counteract it. The Royal Society of Chemistry is not only a professional body for chemists, but also a publisher of peer-reviewed articles. Since publications and citations are influential in the career progression of academic scientists, we have a unique opportunity to follow up these previous reports by a more in-depth gender analysis focussed through the publication process from submission to review to community interest. Our purpose for the current study is three-fold: to present detailed results of our findings; to guide future action items to address points for concern in the accompanying report; and to provide advice for others who wish to undertake such analyses.

There have been previous studies into gender influences of science publications.^3^–^16^ Other publishers have interrogated their own publications *e.g.* Elsevier,^3^ Nature publishing,^4^,^5^ Institute of Physics (IOP) publishing,^6^ Functional Ecology^7^ and American Geophysical Union (AGU).^8^ Studies by the wider community have focussed on a particular publisher *e.g.* Frontiers journals,^9^ or a particular journal *e.g.* eLife,^10^ New Zealand Journal of Ecology,^11^ Behavioral Ecology^12^ and Journal of the American Society for Information Science and Technology.^13^ Some have been multi-disciplinary, spanning science and medicine^14^,^15^ and some are more specific to a particular field of interest *e.g.* astronomy.^16^ These studies have given evidence of the under-representation of women as authors,^3^–^6^,^8^–^10^,^14^,^16^ editors,^7^–^10^ reviewers^4^–^11^ and members of editorial boards.^6^ Some of them have investigated acceptance rates for authors,^6^–^13^ some considered gender interactions between different roles in the publishing process *e.g.* reviewers and authors^8^–^10^,^12^,^13^,^15^ and some investigated potential gender bias in citations.^3^,^16^ Most agree that female percentage decreases with seniority of authorship and that female percentages are increasing over time, although the times estimated for gender parity are long without intervention.^14^ We have conducted this study since none of these previous studies has specifically focussed on the chemical sciences and its sub-disciplines, none were so broad in scope as this, and we required analysis of our own publication metadata to understand the demographics of our Chemistry research community better and identify specific points of action relevant to us. We believe that this detailed synopsis would be of particular interest and relevance to the authors, reviewers, readers and editors who make up our Royal Society of Chemistry's community; it shows how their individual contributions make up the bigger picture, especially since they would not usually have access to the data on which it's based. The RSC publishes many journals that cover a range of Chemistry sub-disciplines with a variety of different impact factors and editorial models, which allows us to investigate a wide scope of gender relationships and facets across these.

Here we use techniques described in the methodology section to break down and analyse the stages of the publication process by gender for submissions to RSC journals between January 2014 and July 2018 and inter-RSC citations between August 2011 and September 2018. We have divided this manuscript into stages that broadly align to a stage in the publication process. Section A covers the background gender characteristics of chemical science researchers. We then investigate further the subset of those authors who have submitted articles to the Royal Society of Chemistry in Section B. Our submissions undergo an initial pre-screening process to determine their overall suitability for the journal. In this paper, we investigate whether there are gender differences evident in the decision to reject a submission without peer review or to progress it through, and the editors who decide it (Section C). RSC articles undergo single-blind review, so gender characteristics of reviewers are described in Section D, and the review recommendations that they make in Section E. Publication is not the end of the story though, so we look at various gender issues in citation behaviour in Section G, and the long-term effect of these publication and citation imbalances on the gender distribution of living chemists with highest H-index scores in Section H. We will not cover the gender make-up of RSC editorial boards since this was covered in the “Diversity landscape of the chemical sciences report”.^1^

Within each of these sections, there are numbered sub-analyses to explore different facets. Variables investigated include author position (corresponding and first authors are investigated in the greatest detail), corresponding author country, number of submissions, number of authors and submission date. As publishers of the submissions under investigation, it was also possible for us to subset the articles by reviewer gender, number of revisions, editor gender, editorial model of journal, chemistry sub-discipline, and significantly, impact factor of journal for each sub-discipline. The latter displayed significant trends of decreasing female author proportions of submissions and publications with increasing impact factor.

For every figure shown in this paper the corresponding numbers plotted, with percentages and confidence intervals, significances and *p*-values are given in the tables of the ESI.†

Note that this study reports gender gaps, but has not fully explained their causes. It is more obvious whether bias is the cause of such gaps by conducting experiments which compare multiple reviews of the same research which differ only in their displayed author names and genders, so as to control for other variables, but this was not possible here (since this is outside the usual publication processing pipeline which was being investigated). It should be noted that one such experiment to investigate race and gender bias in the initial review of NIH grant proposals^17^ did not find evidence of bias.

Within this paper, we focus on the technical details of the methodology and analysis. Whilst we make observations, and enter into some basic discussion, wider discussion of the key points and resulting actions are made in an accompanying report to be released by the Royal Society of Chemistry.

## Methodology and materials

Here we will describe the techniques, statistical methods applied, and the dataset to which these were applied.

The techniques presented were used in two important areas: gender mapping and paper categorisation – as both are important in such studies. The first is important not just for transparency but also because we need to understand the biases that are inherent in gender to name mapping. The second is important since chemistry is not homogeneous and we also need to characterise by different sub-disciplines. We attempted to standardise on techniques and methods and chose statistical methods, which were easy to explain, flexible, and could be visualised. Where possible we used a binominal significance test for simpler analytics. For more in depth consideration of multiple variables we used Generalised Linear Models.

The techniques and methods described in this paper were implemented *via* R^18^ within the RStudio environment.^19^ Most graphics were produced using the package ggplot2.^20^

### Techniques

The techniques that we describe are gender name mapping (essential to infer gender from name if it is not explicitly available) and article categorisation (since breakdown of results into sub-disciplines was an important consideration).

#### Gender name mapping

A key requirement of this study was to deduce a person's gender for data sources where that information is not directly captured, such as the dataset of authors of RSC submissions. All sources did, however, record their first names and these could be used to infer gender. To infer gender we used a methodology outlined in the “Gender Profiles in UK patenting report”.^21^ The method was originally devised by Matias^22^ based on name and gender data from US Social Security Administration and the UK Office for National Statistics (ONS) which were collected and cleaned and made available in an open data set Global Name Data.^23^ This name/gender source list was our starting point for gender mapping *via* an in-house R script which performed a simple comparison of each input first name against it to deduce a gender of male, female or unknown. The genders of a test set of first names were calculated and the results manually checked. In addition to manual checking we confirmed that the proportion of female chemistry researchers deduced using gender mapping methods from first name matched the known gender proportion. As a result of this manual checking and validation, we adjusted the list of first names and gender to some extent from its original source, and the reference data set of first names and gender are available at the bitbucket repository linked at the end of this article.

In Section B3 we explore the impact on the scope of this study of using this method by investigating how its results vary with author country, and compare these with an alternative method, the Python-based package “gender-guesser” (version 0.40).^24^ This package uses a similar method to the ONS method above, whereby an input name is queried by comparison against an input data set of names and genders (which has been gathered from a wide range of countries). The name is then assigned a gender of *male*, *female*, *mostly_female* (which we have grouped together with *female*), *mostly_male* (which we have grouped together with *male*), *unknown* or *andy* (androgynous, which we have grouped together with *unknown*).

We found that both methods gave similar results but that the ONS method gave a better yield of Asian names than gender-guesser. As further validation of our method, in Section A, we found that the ONS method gave similar overall female percentages of chemistry researchers to the results of a dataset where gender was explicitly declared.

#### Article categorisation

Our article categorisation system was developed in-house by the RSC in 2013, for the purposes of helping readers to find papers in multi-topic journals, and has been in use in live production since then. It is based on Latent Sematic Analysis.^25^ The system converts documents into sets of terms – these terms comprise single words, common pairs of words in the same sentence, in a particular order, and the titles of journals cited by the document. During development, an initial corpus, containing RSC articles from 2000 to 2013, was used to prepare a term-document matrix, which was transformed by Singular Value Decomposition into 300-dimensional term vectors and document vectors. Document vectors for documents not in that corpus are constructed by weighting and summing the term vectors for the terms in that document. Documents are compared to one another by cosine similarity, which gives a score ranging from –1 to 1, with scores close to 1 representing high similarity and scores close to zero representing un-relatedness. During development, for each of the twelve categories defined in broad alignment with the RSC organisation of its journals (*Analytical*; *Biological*; *Catalysis*; *Chemical Biology and Medicinal*; *Energy*; *Environmental*; *Food*; *Inorganic*; *Materials*; *Nanoscience*; *Organic*; *Physical*), a document vector was prepared, by selecting by hand terms that the developers considered exemplified the category. Each vector was accompanied by a hand-assigned threshold. To classify a document, a document vector is prepared, and cosine similarities with the vectors for each category are calculated. The document is assigned to those categories where the similarity score exceeds the threshold. In cases where no threshold is exceeded, the paper is assigned to the category it has the highest similarity score for.

As mentioned, article categorisation has been used in the live production server as a means of navigating RSC articles and users have found this useful. Basic validation has been performed by comparison of all article/category pairs in our data set of submissions found by this method *versus* those from the categories that the journal belongs to (according to Table ESI_1† mappings) and the results are shown in Fig. ESI_2 in the ESI.† Note that both methods allow the categorisation of each submission into multiple categories, but the article categorisation method finds more categories for each submission with a mean of 1.8 categories per article compared to 1.25 using the journal category method, which is why the total number of mappings is higher for most categories by this method. In general, we see that the trends in article/categorisation pairings for each of the different categories follow the same trends for the results from article categorisation, journal category and the overlap between them.

The lists of terms exemplifying each category, and threshold values, were tuned during development in 2013, and have not been adjusted since then.

### Statistical methods

We used a limited set of statistical methods to try to standardise the analysis given the wide scope defined. We focussed on methods that are standard, easy to use, visualise and understand. We used two main methods – binomial significance test for cases where there is no outcome variable (*e.g.* when we are testing if a number is unusual) and generalised linear models for cases with an outcome (*e.g.* if we change a variable how does it affect the value of a dependent variable – what is the relationship between them). Where necessary, in parallel with both of these methods we used *T*-test calculations to establish whether the difference between weighted means of a variable, *e.g.* number of submissions per author, were significant for different groups, *e.g.* female or male, using the *t.test* function of the *R Stats package* (v 3.5.1).

#### Binomial significance test

This technique was used to derive the significance of differences in proportions seen in sampled populations from a known baseline. The baseline was typically the female proportion of all chemistry researchers but sometimes a more specific one, *e.g.* the average for corresponding authors, was appropriate. The significance of the difference from the baseline female proportion was calculated using the exact Binomial test,^26^ with the two-sided alternative method and we take *p*-values less than 0.05 (applying a confidence interval of 0.95) to be significant. The default confidence interval method was used for simplicity. The calculations were performed using the *binom.test* function from the *stats* package (version 3.5.1) in R. The binomial significance test can identify sub-populations where gender proportions fluctuate significantly from the baseline for gender characterisation. It can also identify stages in the publication process that result in a significant change in the female percentage from the baseline, which might be points of concern.

#### Success rate as a function of gender

While the binomial significance test can highlight stages in the publication that have significantly lower female percentages than the baseline, these fluctuations might have other causes and are not in themselves an indication of gender bias. The calculation of success rates of each stage in the publication process as a function of author gender is a more direct indication of gender disparities. We have calculated the success rate of the different publication stages separately for male and female corresponding authors (and female first authors for comparison with male first authors). A similar technique was previously used in a study of gender diversity in peer review.^10^ The Chi square test of independence was used to evaluate whether there was a significant relationship between gender and frequency of success or not (again, *p* < 0.05 indicates significance) using the *chisq.test* function of the *R Stats package* (v 3.5.1).

#### Binomial (logistic) generalised linear model (GLM)

For more in-depth analyses of the relationship between variables and a binary outcome we used binomial Generalised Linear Model (GLM) models.^27^ These were also used to explore the effects of interactions between variables on a binary outcome. For example, we can use this method to study the relationship between gender of the corresponding author (the outcome) and the chemistry sub-discipline of the submitted article. In the paper we provide the formula for such modelling in the form GenderOfAuthor ∼ ChemistrySubDiscipline.

Furthermore, we present models that partition submissions by journal, chemistry sub-discipline and impact factor to allow gender effects to be observed independent of wide variations across these groups.

It is important to control for quality of submissions so that the effects of gender are more apparent. We would have liked to have a control for quality from as early on in the publication process as possible, so investigated whether a flag that indicates whether a submissions is single-authored could be used as a proxy for quality of a submission. The reasoning behind this is that single-authored papers: tend to be by established researchers (who do not need to include a supervisor as a co-author); indicate a certain amount of confidence; and avoid any ambiguity of mixed-gender or different-sized teams. However, as we demonstrate in Section C2, there is actually a higher proportion of these single-authored submissions that are rejected without peer review compared to multiple author submissions. This means that this would not be an appropriate quality for control. As an alternative, we considered using the control of reviewer consensus of first round reviews where appropriate since articles that are accepted with reviewer consensus might be expected to be less controversially higher quality papers than those where at least one reviewer suggested either rejection or major revisions. This control would also have the advantage that there are multiple reviews for each article which controls for all other manuscript features *e.g.* impact of journal, submission volumes to journal, number of authors, quality of submission *etc.*^28^ However, using a control which is dependent to one of our key outcomes of reviewer decision is somewhat circular, so we have not pursued this. Since we have not found an appropriate control for quality, care should be taken in the interpretation of results since when we subset by gender it is possible that we are not comparing submissions of equal quality and that this may be mis-interpreted as gender bias. Thus, we are identifying gender gaps and differences rather than gender bias.

We used the *glm* function from the *stats* package (version 3.5.1) in R to perform the binomial GLM calculation. This technique allows us to explore the significance of additional variables (for example journal Impact Factor in the example above). We can calculate whether the addition of a variable to a model has significant effect by calculating the Chi square analysis of variance (or deviance) (ANOVA) significance tables^29^ of the model using the *anova* function of the *stats* package (version 3.5.1). GLMs make it relatively simple to study the effects of interactions between, typically categorical, variables. For each model we show plots, equations, Chi-square *p*-values, and significance. The *effect* function of the *effects* package (version 4.0-3) was used to create an output object suitable for plotting with *ggplot2* from the GLM model output.

#### Multinomial (categorical) generalised linear model

Where the outcome is not binomial but has more than two possible values, we have applied a multinomial GLM model which we applied using the *multinom* function from the *nnet* package (version 7.3-12) in R. This could be used in a similar way to the binomial GLM model, with one or more input variables. Again, the *effect* function of the *effects* package (version 4.0-3) was used to create an output object suitable for plotting with *ggplot2* from the GLM model output.

### Data set

The main data set was submission data (authors, editors, reviewers, reviewer recommendations, journals, dates and outcomes for each revision) regarding all 717 108 articles submitted to RSC journals from January 2014 until July 2018. Author, editor and reviewer genders were not available directly, so were inferred from first names using gender-mapping methods as described in the methodology.

Female percentages were calculated as the percentage of the population with known gender, so that people with unknown gender were omitted from the percentage calculation. In this study we are primarily focused on the difference between male and female genders which may be more apparent when comparing names that are more clearly associated with a particular gender. Inclusion of more gender ambiguous names that could not be assigned a gender reliably would introduce other considerations. For example, we consider the geographical implications of this scope definition further in Section B3. However, we highlight that the approach gave good agreement between baseline female percentages calculated from different sources, regardless of how the gender was obtained, geography and sample size (see Section A3 for further background to this decision).

For the GLM models, this meant that for any model we filtered out any data with an unknown gender in the outcome or variables being tested in that model.

Much of this study is broken down into “original submissions” – this terminology is used to clarify potential ambiguity regarding multiple revisions being submitted for each article. An “original submission” counts all of the multiple revisions of an article with a particular manuscript ID only once. Some sections of this paper consider breakdowns per original submissions, some consider breakdowns per review of each revision of those original submissions, and some refer to the final outcome of each original submission which is the outcome of the last revision within the time period that the data was gathered.

## (A) Background chemical sciences gender landscape

A baseline female percentage for chemistry researchers is a necessary starting point for comparison throughout the publishing process. The UNESCO Institute for Statistics (UIS) reported the percentage of women in all science research (not just Chemistry) as 28.8%.^30^ We used three different data sources and approaches to calculate this baseline female percentage.

### (A1) Higher Education Statistics Agency (HESA) female researcher percentage

The first approach to finding an appropriate baseline was to look at data provided by HESA‡
‡This publication contains data provided by the Higher Education Statistics Agency (HESA), based on the HESA Student and Staff record from 2015/16. Copyright: Higher Education Statistics Agency Limited. Neither the Higher Education Statistics Agency Limited nor HESA Services Limited can accept responsibility for any inferences or conclusions derived by third parties from data or other information supplied by HESA Services. for students and staff for the academic year 2015/16. Staff were restricted to UK chemistry staff who perform research. The HESA student dataset was included since PhD Students are also chemistry researchers and contribute to the authorship of publications. See footnote §
§Staff numbers are obtained from HESA staff dataset by summing the full-time equivalent (FTE) numbers filtered by: “Session population marker” = “Contract counted within session population”; “Cost Centre” = “(113) Chemistry”; “AcademicEmploymentFunction” = “Academic contract that is both teaching and research” or “Academic contract that is research only”; “SOC marker ((311) Science, engineering and production technicians/Other)” = “Other”; “Contract levels” = “A0 to C2 Senior management” or “D and E Head of Schools/Senior Function head” or “F1 Professor” or “I0 Senior/principal lecturer, Reader, Principal Research fellow” or “J0 Lecturer, Senior Lecturer, Senior Research Fellow” or “K0 Lecturer, Research fellow, Senior research assistant, Teaching fellow” or “L0 Research assistant, Teaching assistant”. Chemistry postgraduate students from the HESA student data set were included by summing the FTEs of the HESA student record with the following filters applied: “StudentPopulationMarker” = “The record is counted within the standard HE registration population”; “StudyLevel” = “Doctorate”; “Subject2DigitJACS” = “(F1) Chemistry”; “Session population marker” = “The instance is counted within the HE session population”. for details of the populations that were included and not included in order to consider only research staff and doctorate students who might publish academic articles.

According to these interrogations, there are 9255 UK chemistry researchers (staff and PhD students) of which 33.6% are female. Note that according to HESA rounding strategy, this percentage has been calculated from numbers that were rounded to the nearest 5. While it is possible for the gender recorded by HESA to have the values “male”, “female” or “other”, the numbers with “other” gender round to zero under this rounding strategy.

Within this headline gender distribution, there are differences by seniority of job, as can be seen below.

**Fig. 1:**
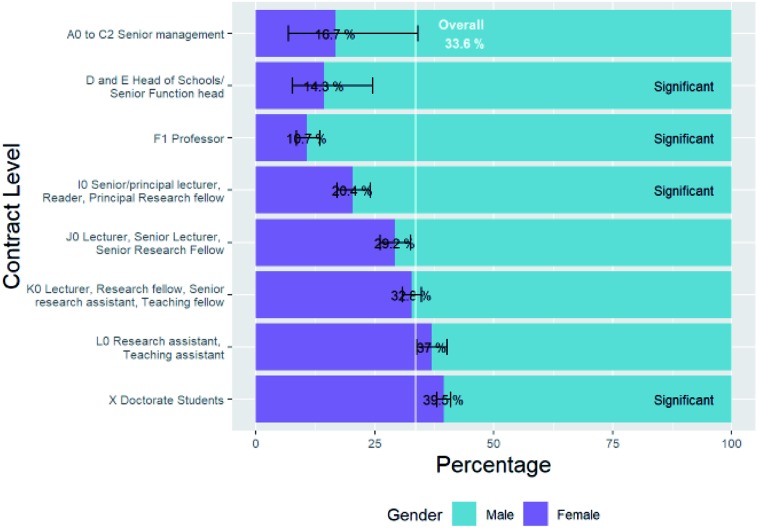
Percentage breakdown of chemistry researcher gender by HESA contract level.

Fig. 1 shows a breakdown by contract level for the staff dataset, additionally with all doctorate students from the student dataset shown in the lowest row. The same filters were applied as above.

A steep drop-off of women with rising “seniority” is in line with the RSC breaking the Barriers report^2^ (“the Leaky Pipeline”). Above the *professorial* level, the sample size reduces considerably which means that trends are no longer significant.

The HESA data suggested that the percentage of chemists who are female is 33.6%, and the number of UK research chemists who are female is 3110.

### (A2) RSC membership data female researcher percentage

Another data source available to us as the Royal Society of Chemistry is the details of our membership as the UK's professional body for chemical scientists. See footnote ¶
¶RSC Membership data was filtered to only include members with membership levels: “Affiliate”, “Affiliate Undergraduate”, “Associate Member”, “Fellow”, “Honorary Fellow”, “Member” and contacts with a job type categorised as “T01 Research” or “T25 Academic” or “T36 Postgraduate (excl. part-time)” and whose Company Type is “54 University”. This data was extracted on 28/6/2018. for data extraction details to consider only chemistry researchers by restricting by membership level and job type categories. Gender was inferred from members' first names, as described in the methodology.

We found that there were 10 023 RSC members who met these criteria of whom the gender of 8197 could be deduced, of whom 31.6% were female.

**Fig. 2:**
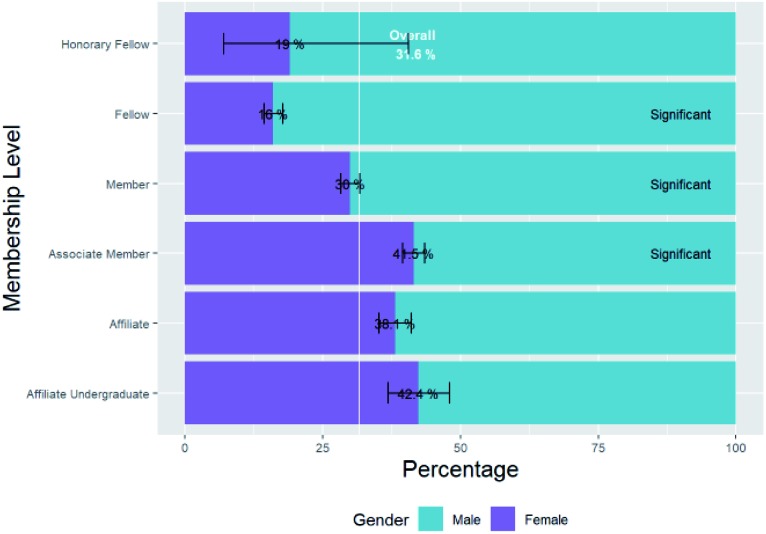
Percentage breakdown of chemistry researcher gender by RSC membership level.

To get a better picture of the gender distribution in chemistry research, this overall female percentage has been broken down into that at different levels of RSC membership included. The results have been plotted in Fig. 2.

Again, we see a decrease in the overall numbers as the levels increase in seniority, ascending the plot.

RSC membership data suggested that the percentage of UK research chemists who are female is 31.6%, and the number of chemists who are female is 2590.

### (A3) All authors data female researcher percentage

The final method of estimating the baseline female percentage of chemistry researchers was to look at the female percentage of all authors of our data set of all submissions to RSC journals for the last 3 years. All authors were considered – not just corresponding or first authors, and were de-duplicated by name. Our best approximation of uniqueness of authors, in the absence of ORCID or other unambiguous author identifier, was established by deduplication of combined first name, middle name and last name, although there were some instances with an unfeasibly frequent number of submissions within this time period (as will be discussed later). Based on all authors of RSC submissions during this time-period there were 637 499 chemistry researchers of whom the gender of 310 377 could be deduced, of whom 35.8% are female. This number is in broad agreement with the proportion of all published Chemistry authors in an extensive multidisciplinary gender analysis of publications in PubMed and arXiv databases which gives a proportion of 34.7–35.4%.^14^ It was noted there that in comparison with the 112 other science disciplines analysed there, that this placed chemistry in topics with fewest female authors.

There is good consistency between the female percentages of all three potential baseline sources (HESA, RSC membership and all RSC authors) despite differences in their sample sizes, geographies and methods of obtaining them. We have chosen to use the value based on all RSC authors, which sets the baseline of female percentage of chemistry researchers at 35.8%, since this is most relevant to the populations and methods being interrogated further in this report. The consistency in the baselines was also considered to validate our methods for estimating gender from first names, as the proportions from HESA where gender was directly known matched those of the chemistry researchers where gender was not directly known but inferred from first name.

We therefore have a baseline for the number of female chemists of 35.8%. However, note that in some cases we will use different baselines specific to corresponding authors, first authors and reviewers when breaking down fluctuations within these groups.

## (B) Gender characteristics of submissions to RSC journals

This section describes investigation of possible gender imbalances apparent during the submission process of articles to the Royal Society of Chemistry.

### (B1) Female percentage of submissions by author role

In the previous section, we considered the female percentage of all authors regardless of role, but is the female percentage of the subset of these who are corresponding authors, or first authors significantly different from the general baseline percentage of female chemists? Again having de-duplicated by name, we see that there are 121 785 unique corresponding authors of whom the gender of 64 396 could be deduced from their first name, of which 29.2% are female. There are 213 281 unique first authors of whom the gender of 103 246 could be deduced from their first name, of which 36.9% are female.

The female percentage of corresponding authors is significantly lower than the baseline of female percentage of chemistry researchers. The female percentage of corresponding authors is closest to the female percentage of HESA chemistry researchers at the level “Lecturer, Senior Lecturer, Senior Research fellow” in Section A1. This is in line with the general convention that corresponding authors are largely heads of research groups.

The female percentage of first authors is significantly higher than the baseline female percentage of chemistry researchers.

The female percentage of first authors is closest to the female percentage of HESA chemistry researchers at the levels “Doctorate students” and “Research assistant, teaching assistant” in Section A1. This is in line with the general convention that first authors are largely the researchers who conduct the research and primarily write it up.

However, we will not consider and compare female percentages of *de-duplicated* female authors of submissions as baseline figures in in the rest of this paper, but rather female percentages of authors of original submissions *without deduplication*. Because of this, we will use the percentages of *original submissions* from female corresponding authors and first authors: 23.9% and 33.4% respectively. These differ from the percentages of unique authors above due to skew caused by some authors having submitted many articles, as will be discussed in the next section.

It is possible to identify the cases where the corresponding author is the same person as the first author (by matching their names). There are 234 317 original submissions from authors whose genders could be assigned and where this is the case, of which 26.5% are female. This lies between the two percentages for first and corresponding authors. The typical case where the corresponding author is the same as the first author might be that of early-career researchers who are establishing their own independent research. They are more senior than typical first authors are since they can address the correspondence regarding the publication themselves, but are not as senior as typical corresponding authors who are research group leaders rather than those who conduct the research themselves.

### (B2) Female percentage of submissions by number of original submissions

In the previous section, there was a difference between the female percentage of authors and the percentage of submissions by female authors. To investigate the difference between these further we look at trends in the gender of each author with the number of original submissions from them over the three-year period for which we have data.

**Fig. 3:**
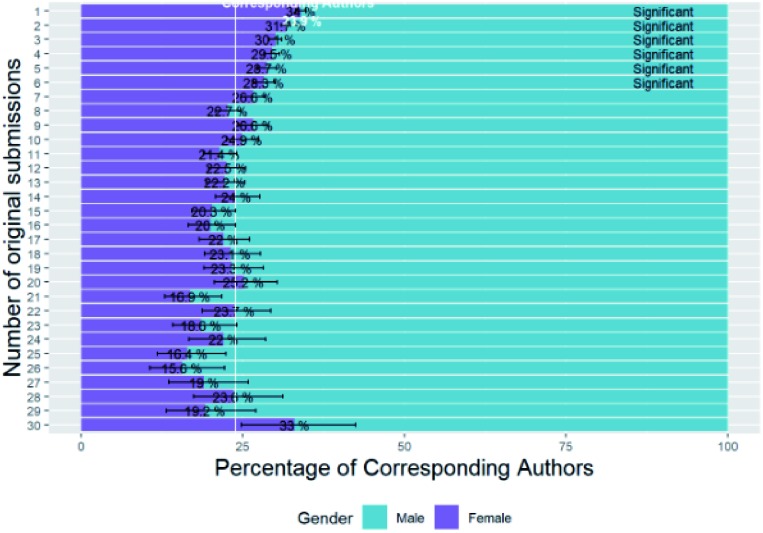
Percentage breakdown of corresponding author gender with number of submissions.

Fig. 3 shows the relationship between corresponding author gender with the number of submissions and the analogous graph for first authors is in Table B2b of the ESI.† The female percentage baseline shown is the average for original submissions from corresponding authors. One point for concern is that there are up to 610 original submissions from each unique corresponding author name (based on first name, middle name and last name) and 522 from each first author name. Names that correspond to the higher end of this scale are not likely to be from a single author but are most likely from multiple authors with the same name. Only numbers of submissions up to 30 are shown, to filter out this common name problem, and because significances are low for higher submission numbers due to the low sample sizes involved. For both first authors and corresponding authors there is a steady drop in the percentage of females as number of submissions increases although care should be taken as the confidence intervals also increase due to smaller sample sizes as the number of initial submissions increases.

Summarising these figures (for all numbers of submissions with no cut-off), the mean number of submissions for female corresponding authors (5.40, standard error = 0.083, *n* = 18 704) is significantly lower than for male corresponding authors (7.25, standard error = 0.070, *n* = 45 378) (two sample *t*-test, *p* = 2.08 × 10^–64^). Likewise, the mean number of submissions for female first authors (3.84, standard error = 0.042, *n* = 37 774) is significantly lower than those for male first authors (4.55, standard error = 0.036, *n* = 64 436) (two sample *t*-test, *p* = 9.01 × 10^–38^).

This lower submission rate of female authors is the source for our observation in Section B1 that the percentage of *original submissions* from female corresponding and first authors was less than the percentage of unique female corresponding and first authors respectively.

### (B3) Female percentage of submissions by country

**Fig. 4:**
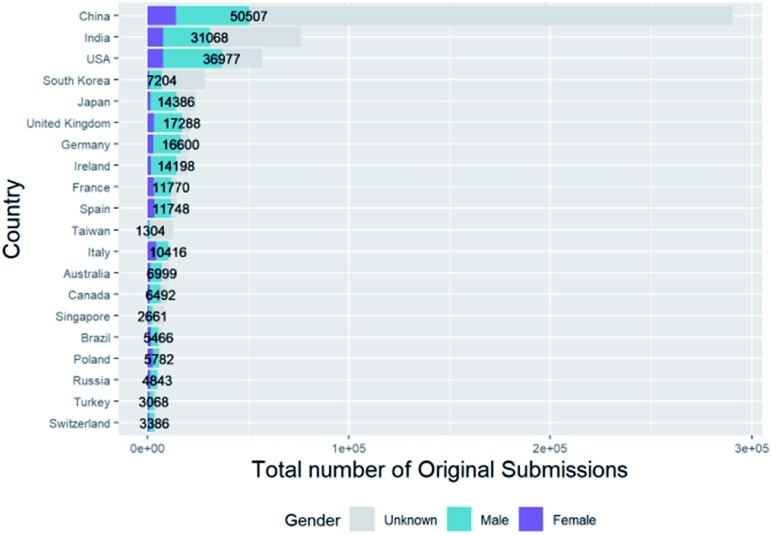
Total breakdown of submissions by corresponding author gender and country for top 20 countries (including unknown gender).

Geographic effects are important to consider. While we do not have country data for all authors, we do have the correspondence address for the corresponding author at the time of publication. The corresponding authors come from a total of 157 different countries but for clarity, we show only the distribution from the top 20 countries in terms of submissions in Fig. 4 (total numbers, including unknown genders).

The country where most of the corresponding authors who submit to the RSC are from is China. However, the majority of these authors do not have first names whose gender can be deduced from their first names and similarly, India, South Korea, Taiwan, Singapore and Turkey have very high proportions of corresponding authors with unknown genders. This is because our gender-assignment code uses Westernised name data sets and is not as successful with non-Westernised names. This issue would not easily be solved by simply using a more geographically diverse reference name-gender data set for comparison since, for example, it is common for Chinese, Indian, South Korean, Taiwanese and Singaporean names to be non-gender-specific and there are additional ambiguities when Asian names are converted into Latin alphabets for matching.

**Fig. 5:**
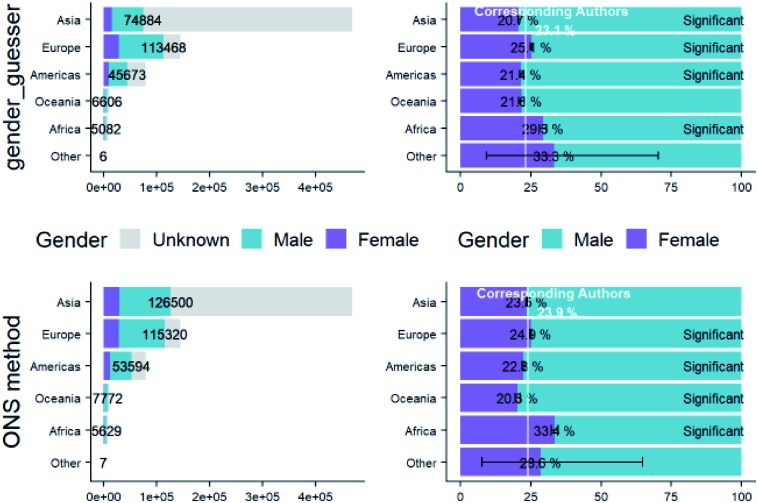
Total (left-hand) and percentage (right-hand) breakdown of corresponding author gender calculated by ONS method (top row) and gender-guesser method (bottom row) with continent of corresponding author address.

In our methodology, we stated that we would omit people whose gender was unknown from percentage calculations and analyses. We can see from these figures that their inclusion would complicate the results and their interpretation-comparing traits of those with unknown gender alongside male and female would not simply compare gender differences between these sample sets, but also introduce geographical differences. We should however be aware that, as we can see from Fig. 5, by omitting people with unknown gender we have effectively filtered out more people from Asian countries.

To explore whether a potential alternative method might increase the inclusion rate of non-Westernised author genders we have performed an additional comparison of our gender-inference method (which we will refer to as the “ONS method”) with that of another method, “gender-guesser”. Using this program, we calculated the genders of the corresponding authors, and compared them with those calculated by the ONS method. The percentage of genders of corresponding author names that matched those from the ONS method were 82.7%, which is good agreement. The distribution of these corresponding author genders by continent in Fig. 5 are very similar to our ONS method and indeed, the ONS method is able to assign more genders to Asian corresponding authors than gender-guesser although we should note that analysis of only known genders by both this ONS method and gender-guesser will under-represent Asian corresponding authors. In fact, of the submissions from corresponding authors with unknown gender (from the ONS method), 84.7% are from Asian countries by this breakdown. We discuss the implications of this further in the Conclusions of this paper.

We will explore geographical imbalances further in subsequent studies but the scope of this study focuses on gender disparities, and these will be more apparent by comparison of submissions from authors with names which are more readily associated with a particular gender.

### (B4) Submission co-authorship characteristics

We now consider various traits of co-authorship of submissions: number of authors; author position; and relationship between first author gender and corresponding author gender.

**Fig. 6:**
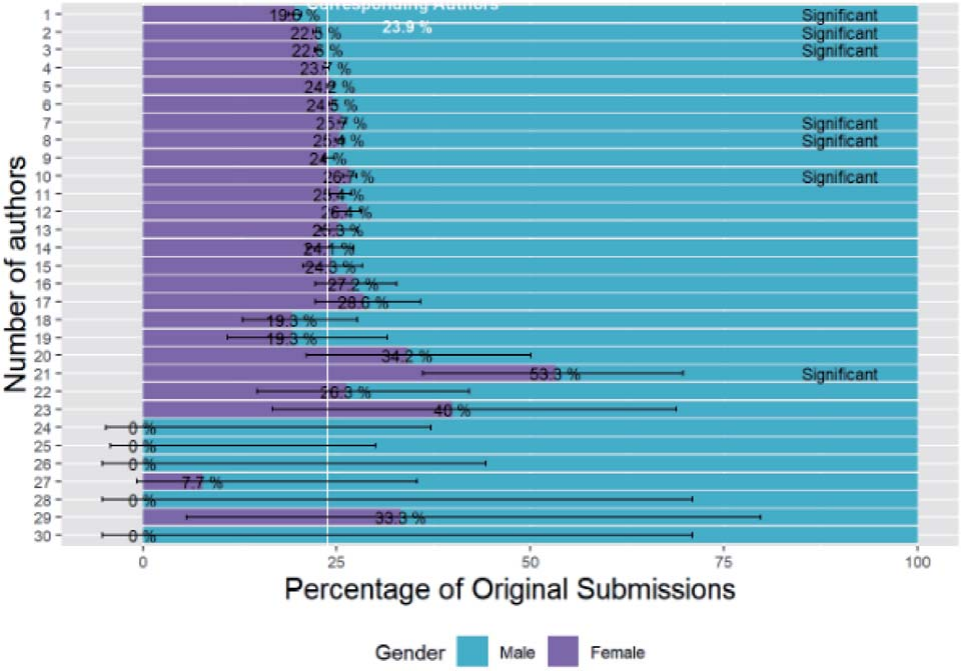
Percentage breakdown of submissions by corresponding author gender and number of authors (having omitted unknown gender).

In Fig. 6 we investigate whether the number of authors of articles changes with the gender of their corresponding author.

The most common number of authors for articles is five and it is apparent that there is a steady decline of female percentage of corresponding author with decreasing number of authors for articles with five or less authors. This is most apparent for single-authored papers for which there are significantly less female authors than expected from the baseline at 19.6%. The mean number of authors for submissions from female corresponding authors (5.28, standard error = 0.009, *n* = 74 306) is significantly greater than that for male corresponding authors (5.12, standard error = 0.005, *n* = 236 310) (two sample *t*-test, *p* = 4.88 × 10^–47^).

**Fig. 7:**
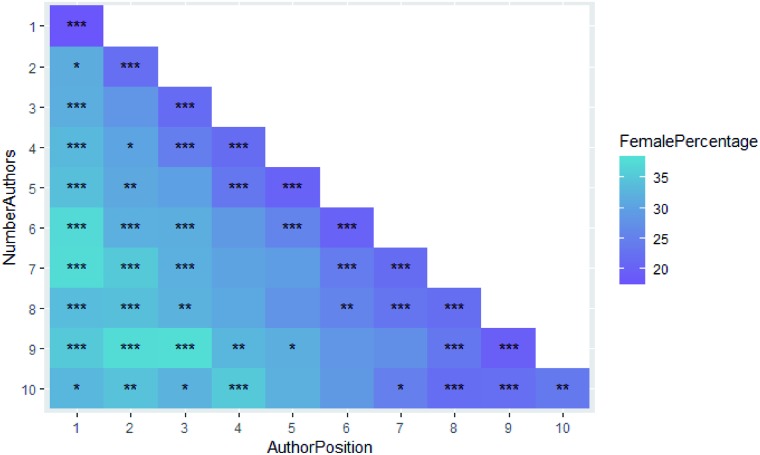
Percentage of female authors with author position and number of authors – asterisks indicate the significance of the binomial *p*-value: *** is highly significant (*p* < 0.001); ** is very significant (*p* < 0.01); * is significant (*p* < 0.05); and no value implies not significant.

In a previous investigation for internal use at the Royal Society of Chemistry we investigated some of these effects, *e.g.* gender distribution of single-authored papers and author position of female researchers, in more detail and here we will summarise the results. All authors (not just corresponding or first authors) were considered for publications to RSC journals from 2016 to 2018 and genders were assigned by the same mapping methods as in this study. Submissions were divided into sets defined by number of authors, and then for authors at each position in the author list the percentage of female authors ascertained, and we used a binomial significance test for comparison against the overall background female proportion. These percentages have been plotted in Fig. 7 for the various numbers of authors and author positions for which they were calculated and asterisks indicate the significance of the *p*-values of their binomial significance tests.

Low female percentages are shown in purple and indicate that males are more likely to appear towards the end of the author list – traditionally the places held by the heads of research groups. Female enrichment is indicated by turquoise and is more likely to appear at the start of the author list (as we have seen in Section B1 – the female percentage of first authors is higher than all authors and corresponding authors). Other observations with this data set are that:

• Female corresponding author papers involve more institutions (*p*-value = 0.004).

• The longer the author list, the more likely they are to contain a higher female/male ratio.

• Female corresponding author papers involve more authors (*p*-value = 1 × 10^–6^).

We have separately investigated the percentage of female corresponding and first authors, and will now investigate how the female percentage of corresponding authors differs with first author gender. Cases where the first author and corresponding author are the same person have been removed since these will skew results towards same-sex authorship and not truly reflect co-authorship trends. The full set of figures for the breakdown is given in Table B4b of the ESI,† but to summarise, the female percentage of corresponding authors for female first authors is 27.7% but that for male first authors is 19.2% and both differ significantly (*p* = 7.30 × 10^–75^ and *p* = 9.61 × 10^–200^ respectively) from the baseline of the average for corresponding authors of 23.9%. We can see a tendency for female corresponding authors to publish with female first authors.

We observe the “gender homophily” (higher than expected occurrence of men co-authoring with men and women co-authoring with women) that has been noted previously in the life sciences.^15^

### (B5) Female percentage of submissions by date

**Fig. 8:**
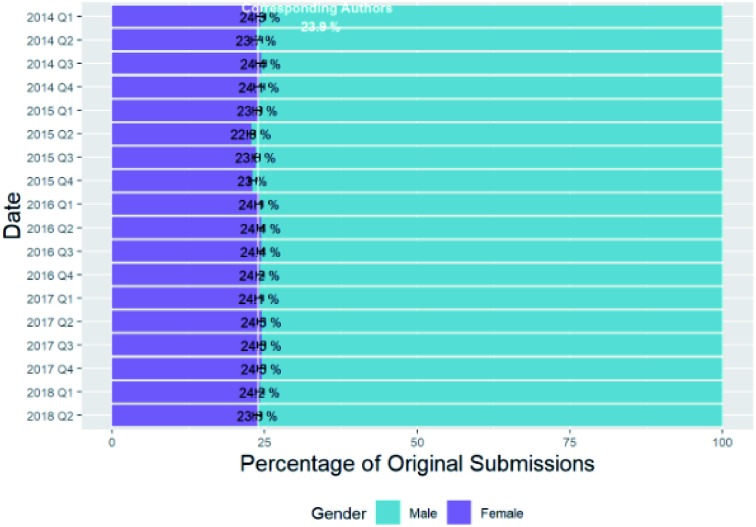
Percentage breakdown of submissions by corresponding author gender and date.

In Fig. 8 we investigate whether there is any change in the female authorship of submissions with time and the analogous graph for first authors is in Table B5b of the ESI.†

There is no obvious trend, based on quarter, of the percentage of female corresponding authors or first authors since 2013. It should, however, be noted that this is a short time span to monitor changes over, especially in comparison with the other similar gender studies mentioned in the introduction, the majority of which showed tendencies towards gender parity over time.^9^

### (B6) Female percentage of submissions by chemistry sub-discipline and impact factor

**Fig. 9:**
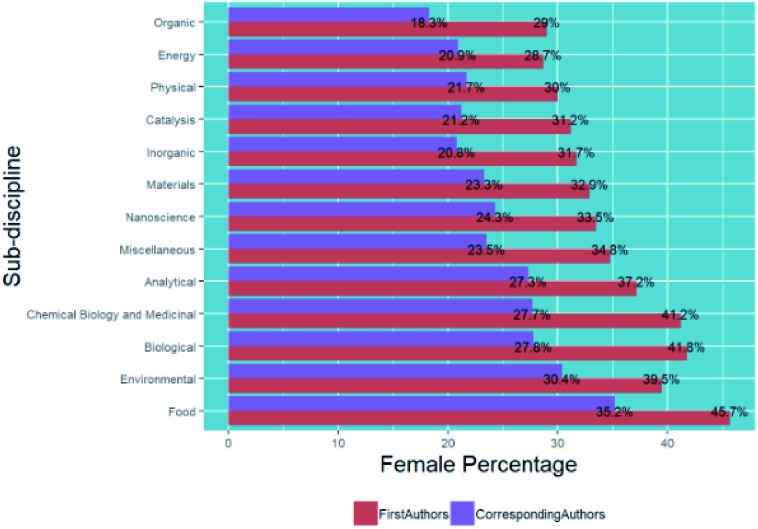
Percentage breakdown of submissions by corresponding and first author gender and chemistry sub-discipline.

Within the general field of chemical sciences, there are sub-disciplines, so in Fig. 9 we show a breakdown of submissions categorised by chemistry sub-discipline (using methods described in the methodology section) and corresponding and first author gender.

In general, we see similar trends for the female percentage of corresponding authors and first authors in the different chemistry sub-disciplines – for example, *Food* and more biological subjects show higher female percentages, and *Organic*, *Inorganic*, *Physical and Energy* show lower female percentages. Although our exact methods differ from those reported by the IOP in their similar analysis,^6^ our values of 39.5% for submitted corresponding authors of environmental articles and 32.9% for materials articles are in line with those that they found of 39% and 32.9% respectively.

We have seen how author gender differs within chemistry sub-disciplines, and can now consider the effect of a second variable – impact factor – on the corresponding author gender within each chemistry sub-discipline by applying a GLM model. Comparisons involving impact factors across all sub-disciplines should be avoided, since there are large differences between the typical number of citations for different fields, which is why we have modelled and presented results separately for each sub-discipline. Impact factors are given with other journal information in Table ESI_1 of the ESI† and are based on 2017 Journal Citation Reports® (Clarivate Analytics, June 2018). Note that impact factors of *Energy & Environmental Science* (30.06) and *Chemical Society Reviews* (40.182) were omitted to give a more consistent range of impact factors across the different journals over which the models are fitted.

**Fig. 10:**
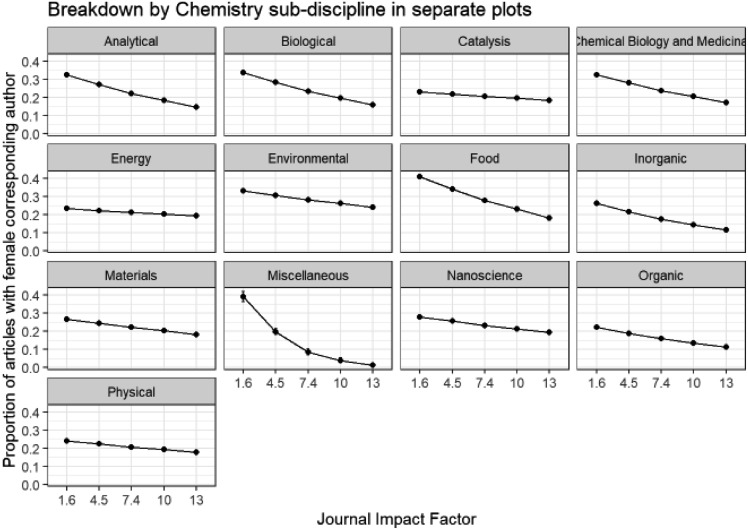
Binomial GLM model of corresponding author gender of original submissions, chemistry sub-discipline and journal impact factor (Model: CorrAuthorGender ∼ Category * ImpactFactor). ANOVA Pr(>Chi): category is highly significant (0.00 × 10^0^); ImpactFactor is highly significant (0.00 × 10^0^); category: ImpactFactor interactions are highly significant (3.75 × 10^–97^).

In Fig. 10 we see that within all of the chemistry sub-disciplines there is a decline in submissions from female corresponding authors with increasing impact factor of journal and this is also seen in the analogous plot for first authors in Fig. B6d in the ESI.† This tendency for female authors to submit to lower impact journals may indicate an attempt to minimise risk of rejection, or may be the result of previous rejection from higher impact journals. It is a point for concern, potentially limiting the take-up and impact of the published research of female authors compared to male though. The greatest drop-off of corresponding author female proportion with impact factor is observed in *Food* and *Miscellaneous* sub-disciplines. The mean impact factor of journals of female corresponding author submissions (4.86, standard error = 0.009, *n* = 71 292) is significantly lower than that of male corresponding authors (5.15, standard error = 0.005, *n* = 224 404) (two sample *t*-test, *p* = 2.49 × 10^–198^).

## (C) Gender characteristics of editors and rejection without peer review

After submission, the next stage in the publishing process involves an editor being assigned to each submitted article to conduct an initial assessment of its suitability for the journal and publication. If it is not suitable, the editor can reject it without peer review, or transfer it to another journal for consideration, or progress it through to peer review. We will investigate gender disparities in this stage of the publishing process in this section.

### (C1) Gender characteristics of editors

**Fig. 11:**
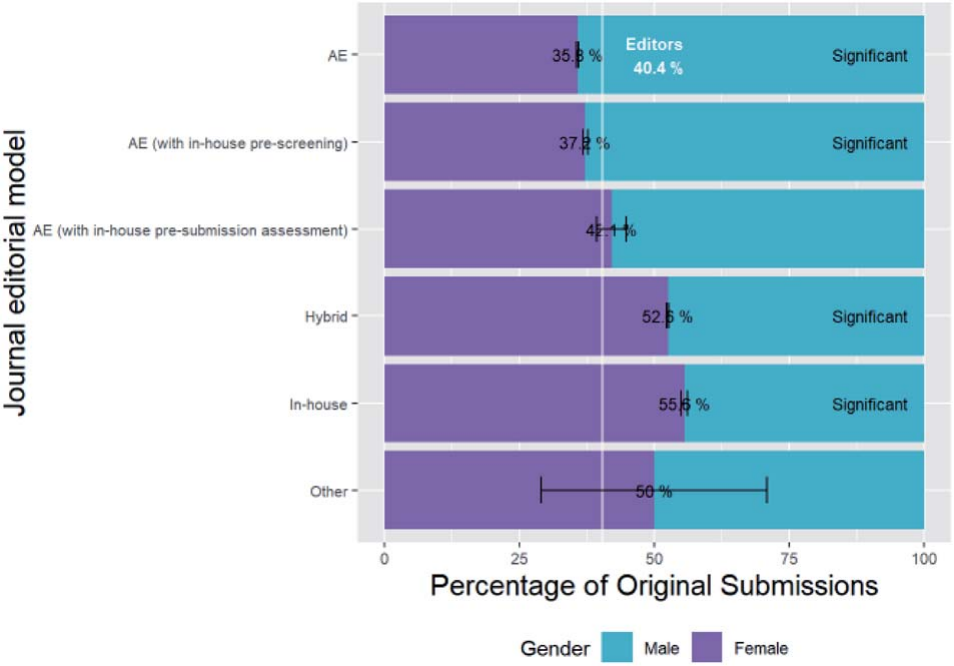
Percentage breakdown of submissions by editor gender and journal editorial model.

We obtained the names of all the handling editors of each submission, de-duplicated them and assigned genders where possible. The genders of 691 of the 936 unique editors could be deduced, and of these, 40.4% were female. This is significantly higher than the baseline of 35.8% for chemistry researchers. Some of these editors are members of RSC staff and some of them are external Associated Editors (AE) depending on the editorial model of the journal (as listed in Table ESI_1 in the accompanying ESI†). Fig. 11 shows the breakdown of editor gender of all original submissions based on the editorial model of the journal.

The female percentage of in-house RSC editorial staff is much higher than that of the external associate editors, who are academic researchers (with a female percentage in line with the baseline for chemistry researchers). This is not surprising since RSC in-house editors are not academic researchers, although many of them were previously graduates from the chemical sciences, either with undergraduate or postgraduate degrees. Indeed when considering the “leaky pipeline”, the RSC is an example of one of the alternative employment destinations where many female researchers go to having left academia. Editors of submissions to journals with a hybrid model, or other mixtures of internal and external editors, have female percentages somewhere between these two extremes, as might be expected.

### (C2) Rejection without peer review

When a paper is submitted to the RSC, the editor who is handling it will assess its suitability for the journal, and publication as a whole. The percentage of original submissions from female corresponding authors which are rejected before peer review is 25.6% compared to 23.2% which progress to peer review and both differ significantly (*p* = 6.79 × 10^–28^ and *p* = 6.15 × 10^–8^ respectively) from the baseline of the average for corresponding authors of 23.9% (full breakdown figures are in Table C2a of ESI†). It is therefore apparent that there is a small but significant difference between the percentage of original submissions from female corresponding authors which are rejected without peer review and those that go on to be reviewed. To express this in an alternative way, the success rate for female corresponding authors to progress to peer review is 69.31% and for corresponding males it is 71.98% (Chisq test of independence of success frequency with gender is significant with *χ*^2^ (1, *n* = 310 616) = 197.52, *p* = 7.26 × 10^–45^).

For first authors the female percentages of submissions which are rejected before peer review are 34.2% compared to 33.1% which progress to peer review and neither differ significantly (*p* = 0.0631 and *p* = 1.00 respectively) from the baseline of the average for first authors of 33.4% (full breakdown figures are in Table C2a of ESI†). However, the success rate for female first authors to progress to peer review is 69.78% and for corresponding males it is 70.74% (Chisq test of independence of success frequency with gender is significant with *χ*^2^ (1, *n* = 317 710) = 30.78, *p* = 2.89 × 10^–8^). It is thus apparent that female authors have a slightly, but significantly lower success rate for initial rejection without peer review which is more apparent for corresponding authors than first authors.

**Fig. 12:**
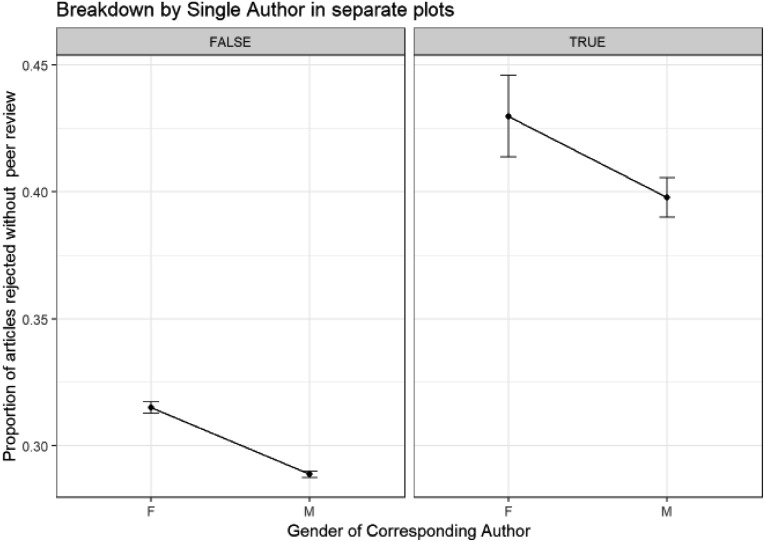
Binomial GLM model of proportion of submissions rejected without peer review and corresponding author gender controlled by whether the publication is single-authored or not (Model: RejectedWithoutPeerReview ∼ SingleAuthor * CorrAuthorGender). ANOVA Pr(>Chi): SingleAuthor is highly significant (0.000); CorrAuthorGender is highly significant (0.000); SingleAuthor: CorrespondingAuthorGender is not significant (0.903).

The above figures apply no controls, so while they give us an idea of overall gender distribution, the reduction in female percentage of corresponding authors through this initial “rejection before peer review” stage and lower female success rates may be because we are not comparing similar types of submissions. To compare more similar sets of submissions we show the results of a GLM model of proportion of submissions rejected without peer review and corresponding author gender with the test control whether the submissions have a single author (as discussed in the methodology) in Fig. 12.

The single-authored submissions show a much higher proportion of rejection at this initial stage of the publication process, which indicates that they are not a good proxy for quality to use as a control. Nevertheless, they are an interesting subset to compare, and a higher proportion of submissions from female single authors are rejected without peer review than male single authors. These proportions are not different from what might be expected for female and male corresponding authors publishing with other (potentially other gender) authors.

### (C3) Rejection without peer review broken down by editor gender and journal editorial model

**Fig. 13:**
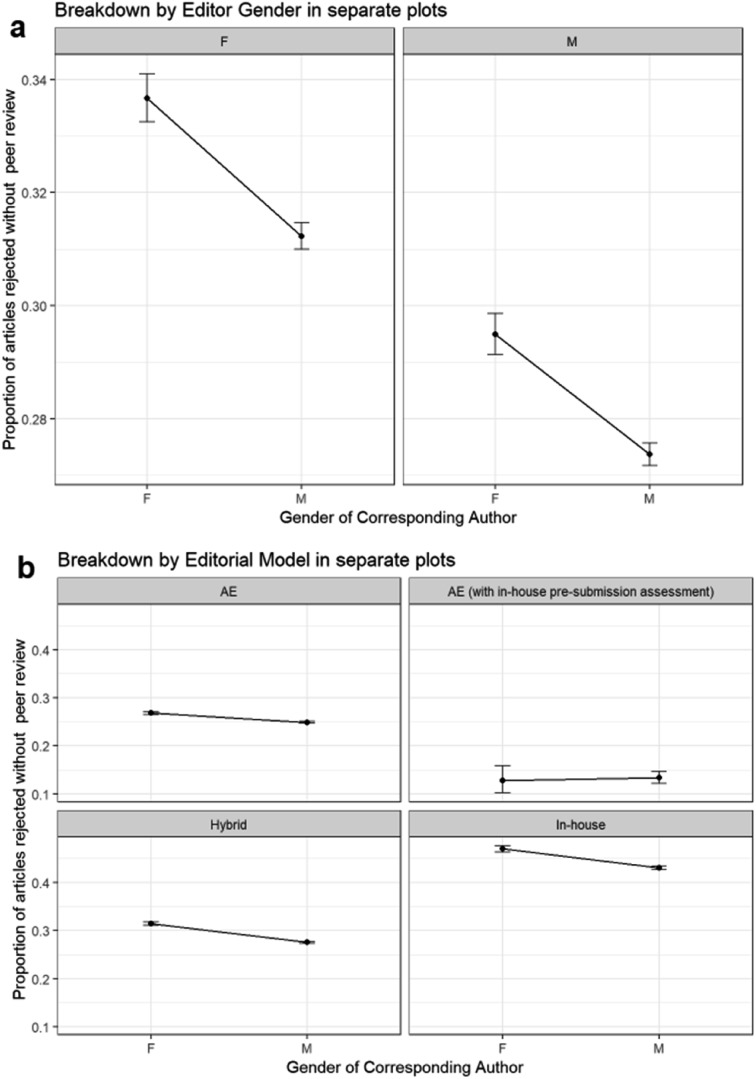
Binomial GLM models of proportion of original submissions rejected without peer review. (a) – Binomial GLM model of proportion of original submissions rejected without peer review, corresponding author gender and editor gender (Model: RejectedWithoutPeerReview ∼ CorrAuthorGender * EditorGender). ANOVA Pr(>Chi): CorrAuthorGender is highly significant (1.71 × 10^–20^); EditorGender is highly significant (8.98 × 10^–79^); CorrespondingAuthorGender: EditorGender is not significant (0.76). (b) – Binomial GLM model of proportion of original submissions rejected without peer review, corresponding author gender and journal editorial model (Model: RejectedWithoutPeerReview ∼ CorrAuthorGender * EditorialModelOfJournal). ANOVA Pr(>Chi): CorrAuthorGender is highly significant (6.18 × 10^–41^); EditorialModelOfJournal is highly significant (0.00); CorrAuthorGender: EditorialModelOfJournal is highly significant (3.07 × 10^–4^).

We can see the effects of editor gender on whether submissions from female and male corresponding authors are rejected before, or progress to peer review, using binomial GLM methods again in Fig. 13a.

We can see from Fig. 13b and its ANOVA output that while corresponding author gender is significant (a higher proportion of submissions from female corresponding authors are rejected without peer review than those from male corresponding authors) and editor gender is significant (a higher proportion of submissions are rejected without peer review by female editors than male editors), there is no significant interaction between these two variables. The proportion of submissions rejected without peer review when both variables are considered is as expected from the given the variables separately. As such, no evidence can be seen that female or male editors are positively or negatively inclined to reject more submissions without peer review from female corresponding authors.

In a similar way, we can investigate whether the level of rejection without peer review of external associate editors is the same as that of in-house editors *via* a binomial GLM model (Fig. 13b).

To simplify the analysis, we have filtered out editorial models with small sample sizes. From the ANOVA results, the editorial model of the journal is significant, both on its own and when it interacts with corresponding author. There are higher rates of rejection without peer review from in-house editors than associate editors with hybrid models lying in between them. “Associate editors with in-house pre-submission assessment” have the lowest rates of rejection without peer review, but note the low sample numbers for these.

From the ANOVA results, the relationship between the proportion of rejected submissions without peer review with editorial model given its relationship with corresponding author is significant. In particular, there is a larger positive difference between the proportion of rejected submissions without peer review for female corresponding authors compared to that for male corresponding authors for journals with in-house and hybrid editorial models – indicating higher gender disparity for these journal models. While “Associate editors with in-house pre-submission assessment” seem to show a much smaller and opposite gender difference, the differences between the male and female corresponding authors are not significant.

## (D) Gender characteristics of reviewers

The next stage of the publishing pipeline is review. Here we will investigate gender issues when choosing reviewers.

### (D1) Gender breakdown of reviewers, their invitations and responses

Firstly, we will consider the overall gender distribution of all of the reviewers of all submissions to the RSC during the 3 years under investigation. There were 118 808 unique (de-duplicated by name) reviewers of which the gender of 68 015 could be assigned, and 24.5% were female. This value is significantly and considerably lower than the analogous female percentages of corresponding and first author of submissions in Section B1 (29.2% and 36.9% female respectively) and editors in Section C1 (40.4% female) and considerably lower than the baseline for chemistry researchers of 35.8%. This might partly be because reviewers tend to be selected from established researchers who are more senior in their career – we note that the female percentage of reviewers is between the female percentages of HESA chemistry staff at the levels “Senior/principal lecturer, Reader, Principal Research fellow” and “Lecturer, Senior lecturer, Senior Research Fellow” in Section A1.

If we consider the percentage of reviews performed by female reviewers (no longer de-duplicated by name) it is even less, at 20.8%. This difference is because the average number of reviews by female reviewers over this time period was 5.69 which is significantly less than the value for male reviewers of 7.05. So not only are there less female reviewers, but each of them perform fewer reviews than their male counterparts do.

Firstly, we will consider trends in reviewer invitations. We explored if there are less women reviewers because less women are invited or because they do not accept reviewer requests as often. During the three years investigated, there were a total of 1 162 024 invitations to reviewers, and of these, the gender of 619 607 could be deduced, 21.1% of which were female. This is not significantly different from the baseline percentage of reviews performed by female reviewers (20.8%), which indicates that the low female percentage of reviews performed reflects the low percentage of invitations to female reviewers.

The full set of figures for the breakdown is given in Table D1 of the ESI,† but to summarise, the female percentage of reviewer invitation responses was 21.5% agreed, 21.7% declined, 19.6% failed to respond and all differ significantly (*p* = 5.00 × 10^–10^, *p* = 1.15 × 10^–20^ and *p* = 1.85 × 10^–28^ respectively) from the baseline of the average for female reviewer invitations of 17.5%. A significantly lower percentage of female reviewers fail to respond to their reviewer invitations than male reviewers (more female potential reviewers respond). Out of those that respond, there is no significant difference between the female percentages who accept or decline their invitations.

We can therefore see that the low female percentage of reviews performed by female reviewers is predominantly due to them being invited less than male reviewers. When the Institute of Physics investigated this issues they found that male reviewers were invited to review more than female reviewers, and that there was “no significant difference in the propensity for men or women to accept review invitations”.^6^ The American Geophysical Union also found that women were invited to review less than men, but that they had a slightly higher decline rate.^8^ The journal Functional Ecology found that women female reviewers were less likely to respond to reviewer requests, but if they did, they were more likely to respond positively.^7^

### (D2) Relationship between reviewer gender, corresponding author gender and editor gender

We will now investigate how the percentage of reviews by female reviewers differs with corresponding author gender. The full set of figures for the breakdown is given in Table D2a of the ESI,† but to summarise, the percentage of reviews by female reviewers for female corresponding authors is 23.9% and that for male corresponding authors is 19.3% and both differ significantly (*p* = 1.54 × 10^–61^ and *p* = 7.26 × 10^–52^ respectively) from the baseline of the average for reviewers of 17.5%.

There are significantly more reviews from female reviewers for submissions from female corresponding authors. This may be because female corresponding authors are more likely to suggest female reviewers for their papers, or because editors are more likely to select female reviewers for these papers or because female reviewers are more likely to agree to review papers from female corresponding authors. We do not have the data to test the first possible contributory factor but we can investigate the second two.

**Fig. 14:**
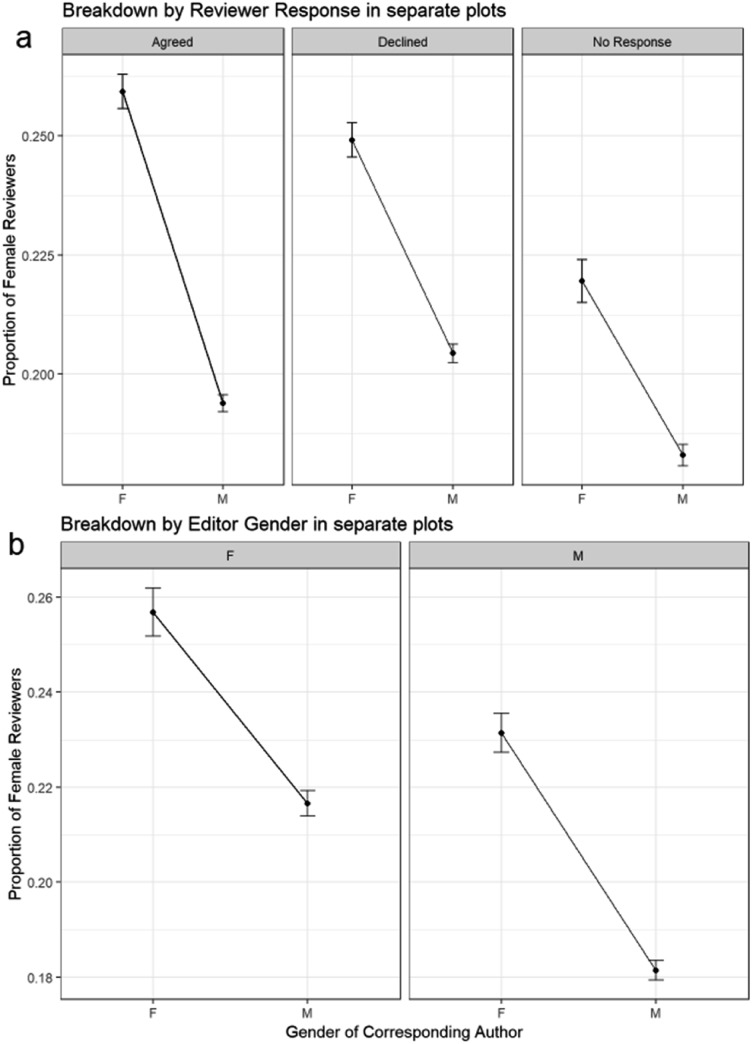
Binomial GLM model of proportion of reviews. (a) – Binomial GLM model of proportion of review invitations by female reviewers, corresponding author gender and reviewer response (Model: ReviewerGender ∼ CorrAuthorGender * Response). ANOVA Pr(>Chi): CorrAuthorGender is highly significant (1.77 × 10^–161^); Response is highly significant (2.13 × 10^–28^); CorrAuthorGender: Response is highly significant (3.08 × 10^–8^). (b) – Binomial GLM model of proportion of reviews by female reviewers, corresponding author gender and editor gender (Model: ReviewerGender ∼ CorrAuthorGender * EditorGender). ANOVA Pr(>Chi): CorrAuthorGender is highly significant (1.64 × 10^–64^); EditorGender is highly significant (1.86 × 10^–46^); CorrAuthorGender: EditorGender is significant (9.04 × 10^–3^).

A binomial GLM model of the outcome proportion of invitations to female reviewers changing with corresponding author gender and reviewer response is shown in Fig. 14a.

The outcome in Fig. 14a is the proportion of all *invitations* that are to female reviewers, rather than the proportion of *reviews*, as in the previous section. However, the same trend is apparent with more invitations going to female reviewers for submissions from female corresponding authors. The biggest difference in female proportion of reviewers with corresponding author gender is observed for reviewers who accept their invitations rather than those who decline or fail to respond. It can be seen that female reviewers accept significantly more invitations for submissions from female corresponding authors and less for submissions from male corresponding authors. From the ANOVA chi square test *p*-value the additional consideration of reviewer response is significant, and so the increased proportion of submissions from female corresponding authors having a female reviewer is a combination of editors inviting them more, and the female reviewers being more likely to accept these papers.

A previous study of publications in Frontiers journals found substantial gender homophily – with editors of both genders showing substantial same-gender preference when appointing reviewers.^9^ In Fig. 14b we consider the effect on female reviewer proportion of corresponding author gender together with editor gender.

The variable EditorGender has a significant effect on reviewer gender – there is a higher female proportion of reviewers for reviews with female handling editors than male editors. Interactions between corresponding author gender and editor gender are also significant – there is a higher proportion of female reviewers for female corresponding authors for submissions with male editors than female editors.

### (D3) Gender characteristics of reviews by date, number of reviewers and number of revisions

**Fig. 15:**
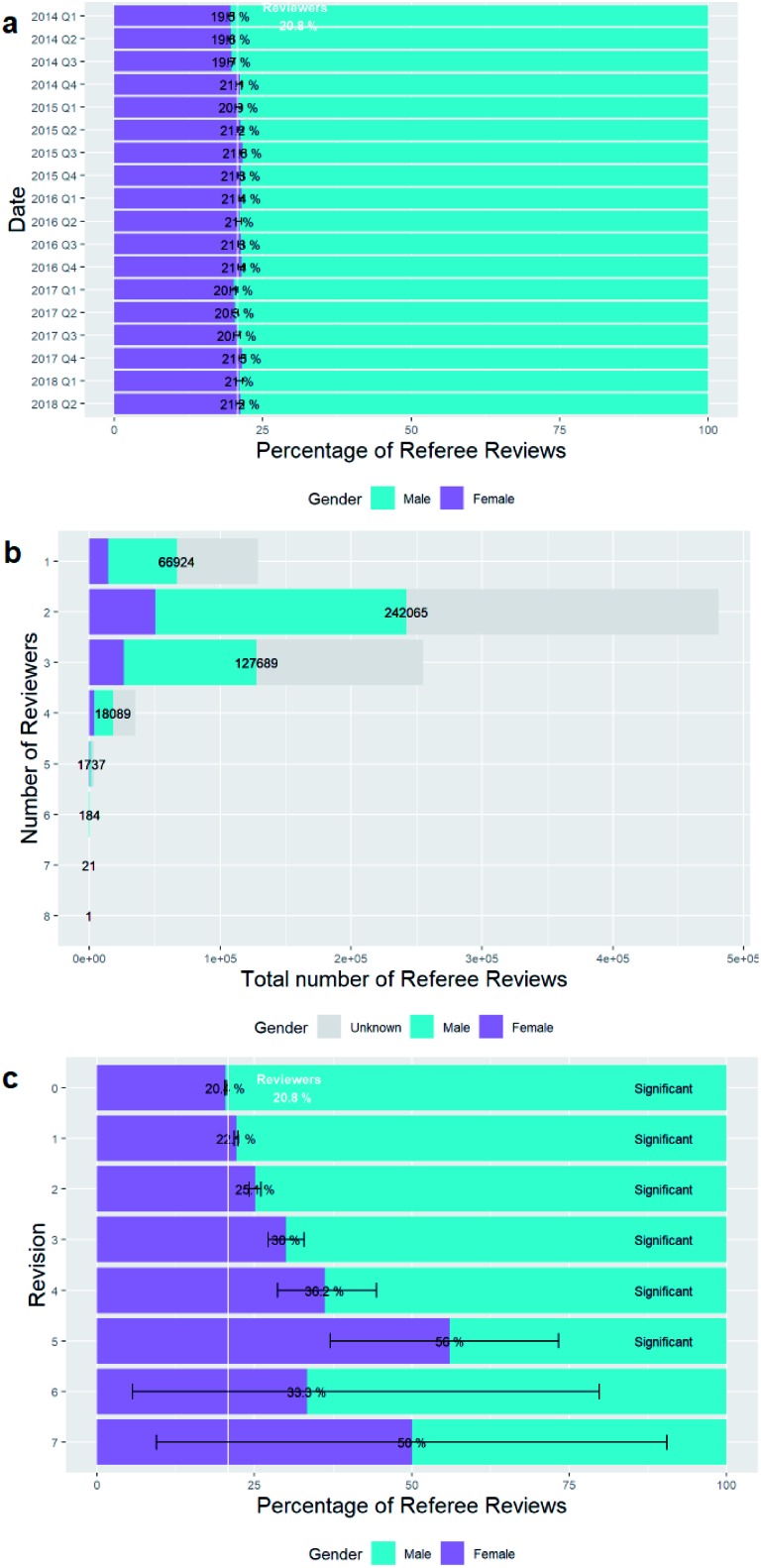
Gender breakdowns of reviews. (a) – Percentage breakdown of reviews by reviewer gender and date. (b) – Total numbers of reviews by reviewer gender and number of reviewers for that submission. (c) – Percentage breakdown of reviews by reviewer gender and number of revisions.

In Fig. 15a we investigate how female reviewership of submissions changes with time (grouped by quarter).

There was a slight increase in the female percentage of reviewers through 2014 but there have been no significant changes in the percentage of female reviewers since then.

There is no set number of reviewers required for a submission. Fig. 15b shows the distribution of the total number of reviewers for each submission, and the numbers plotted and their percentage breakdown by gender is given in Table D3b of the ESI.†

If multiple versions of an article are submitted then the number of reviewers of each version are included separately.

We have shown the total numbers plot and not just the percentage gender breakdown to show that most submissions have 2 reviewers, but 3 and 1 are also common. The percentage breakdown of these figures shows no significant difference in the percentage of female reviewers with the number of reviewers for the article revision taking into consideration the small sample sizes and large confidence intervals where there are more than 4 reviewers.

Likewise, there are no set number of revisions for a submission. When we look at the female percentage of reviewers for each total number of revisions of submissions, as in Fig. 15c we can see some significant trends.

There is a progression to higher percentages of women reviewers for submissions that have been revised many times. This ceases to be significant for more than 5 revisions and the small sample size of submissions which have more than this number of reviews should be noted.

### (D4) Female percentage of reviews by chemistry sub-discipline

**Fig. 16:**
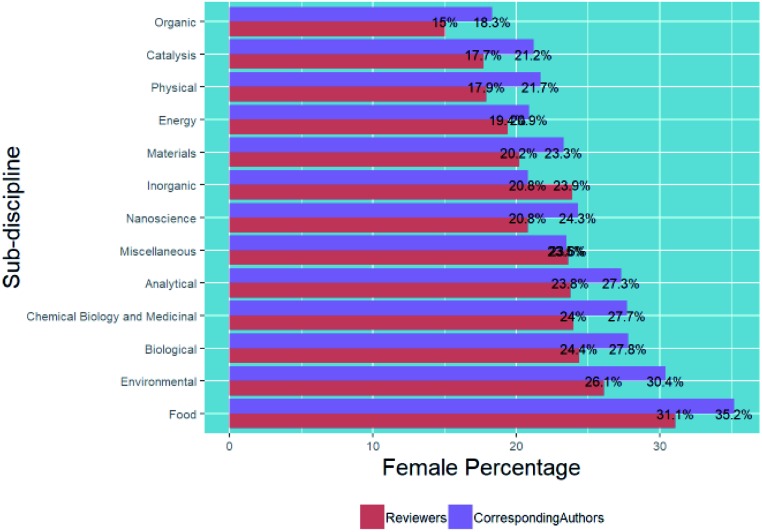
Percentage breakdown of reviews by reviewer gender for each chemistry sub-discipline in comparison with percentage of submissions from female corresponding authors.

In Fig. 16 we observe that the same gender differences by chemistry sub-discipline in Section B6 apply to reviewers as they did to corresponding authors. The chemistry sub-discipline of each article reviewed was assigned by the same methods, and all reviews for each article considered separately.

We see greater differences between the female percentages of reviewers and those of corresponding authors than the differences in Section B6 between corresponding authors and first authors. Although *Food* has the highest female percentage of any chemistry sub-discipline, the female percentage of reviewers is 4.1% lower than that of corresponding authors. In contrast, *Inorganic* reviews have a female percentage of reviewers 3.1% higher than that of corresponding authors. In all, 11 of the categories have a female percentage of reviewers lower than the female percentage of corresponding authors. In particular, sub-disciplines with bigger negative differences between the female percentage of reviewers compared to corresponding authors are *Environmental* (4.3%), *Food* (4.1%), *Physical* (3.8%), *Chemical Biology and Medicinal* (3.7%).

## (E) Gender characteristics of reviews

We have investigated the gender characteristics of reviewers and will now investigate the effect of reviewer and author gender on reviewer recommendation. The primary outcome for this section is reviewer recommendation, and although the headline major *accept*/*reject* recommendation is binomial, this misses out on some important subtleties which are recorded in the reviewers' minor recommendations which has 4 main possible values: *accept*; *minor revisions*; *major revisions*, *reject*. The first three values are grouped together to an “accept” major recommendation. The overall percentages of reviews with each of these possible outcomes in this data set were: 20.1% accept; 28.2% for minor revision; 21.6% for major revision; 30.1% reject. From the set of reviews considered there were some additional recommendations which were recorded but with very low occurrence: *recommend after revision* (12 times); *recommended after revision after revision* (1 time); and *revisions required* (1214 times). When considered in the context of 904 050 reviews these outcomes are small in number, and so these recommendations have been omitted for clarity of analysis results. Nevertheless, this still leaves 4 possible outcome values to investigate, which means that it is necessary to move from a binomial GLM analysis (as used previously in this analysis) to a multinomial GLM analysis (see the methods section for more details). We show plots of all reviews (only limited by reviewer and corresponding author genders which are unknown) and contrast these with plots which only show first revision reviews with a control applied to show whether all reviewers were agreed on the same decision or not.

### (E1) Relationship between reviewer recommendations and corresponding author gender

**Fig. 17:**
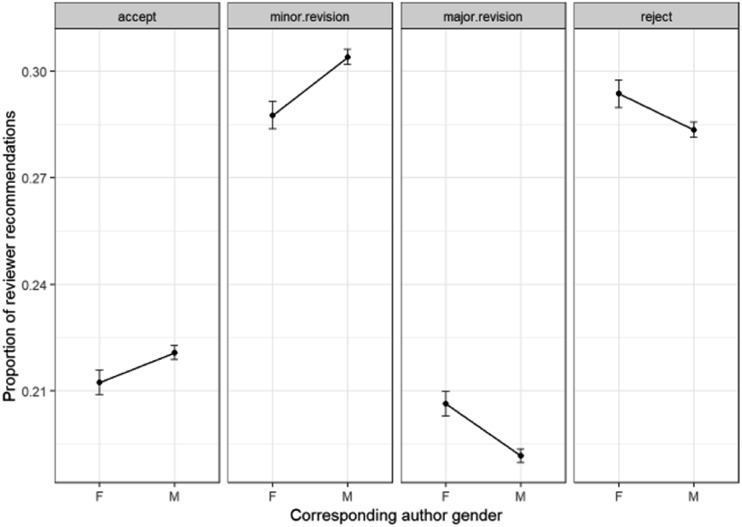
Multinomial GLM model of reviewer recommendations and corresponding author gender (Model: ReviewerRecommendation ∼ CorrAuthorGender).

Fig. 17 shows the results of the multinomial GLM model of the outcome *reviewer recommendation* with corresponding author gender.

While the difference between the extreme outcomes of “accept” and “reject” is not so marked between male and female corresponding authors, the difference is more apparent for the grey areas in the middle – “major revision” and “minor revision”. It is apparent that the two more positive outcomes – “accept” and “minor revision” are less common for female corresponding authors, and the two more negative outcomes – “major revisions” and “reject” are more common for them.

### (E2) Relationship between reviewer recommendations and gender

**Fig. 18:**
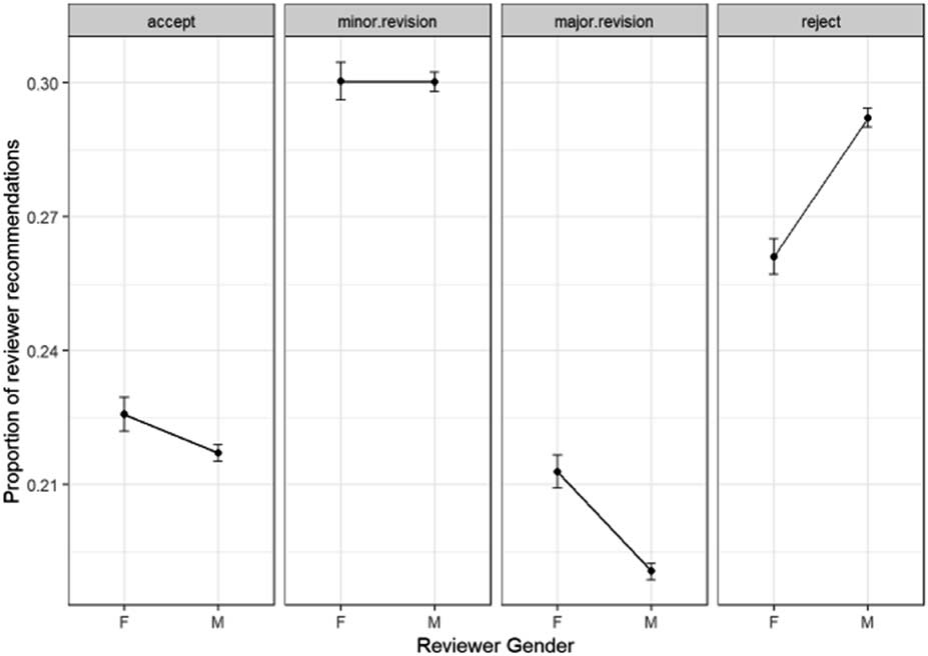
Multinomial GLM model of reviewer recommendations and reviewer gender (Model: ReviewerRecommendation ∼ ReviewerGender).

We will now investigate the effect of reviewer gender rather than corresponding author gender on the reviewer recommendations by multinomial GLM model in Fig. 18.

The strongest difference between male and female reviewers apparent in these plots is that females are less likely to reject papers than males. It is also apparent is that female reviewers are more likely to recommend major revisions than reject a paper whereas the reverse is true for male reviewers. Female reviewers are slightly (but significantly) more likely to accept papers outright than male reviewers.

### (E3) Relationship between reviewer recommendations, corresponding author and reviewer gender

**Fig. 19:**
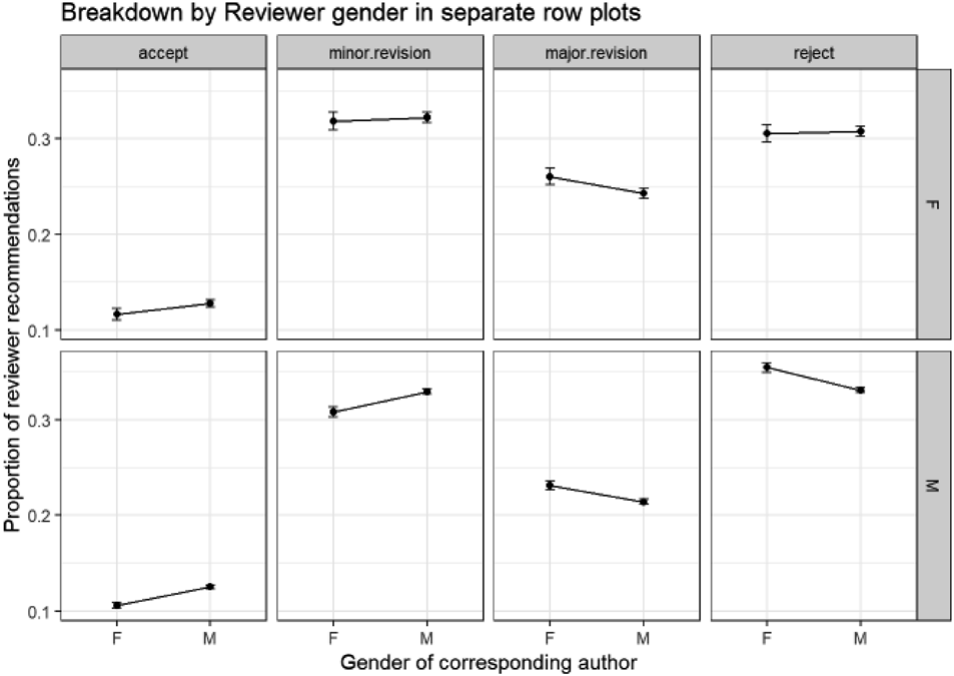
Multinomial GLM model of first round reviewer recommendations, corresponding author gender and reviewer gender (female reviewers on top row and male reviewers beneath) (Model: ReviewerRecommendation ∼ ReviewerGender * CorrAuthorGender). ANOVA Pr(>Chi): ReviewerGender: CorrespondingAuthorGender interactions are highly significant (0.00 × 10^0^).

We will now consider the relationship between the interactions of these two variables of reviewer and author gender and first-round reviewer recommendation using a multinomial GLM. The effects are shown in Fig. 19.

The lower plots in Fig. 19 are very similar to those shown in Section E2 since the majority of reviewers are male, so we would expect this to be the case.

The column with the biggest difference between the female reviewers on the top row and the male reviewers on the bottom row is that for the “reject” recommendations. The difference between female and male reviewers reflects the observation in the discussion for Section E2 that female reviewers are less likely to reject papers than male reviewers are. The additional interaction between the variables shows that while male reviewers are slightly more likely to reject papers from female corresponding authors, female reviewers do not follow this pattern.

The observation in the discussion for Section E2 that female corresponding authors are less likely to have submissions accepted by reviewers than male authors is not the case when the reviewers are female, and likewise for minor revisions. Indeed, there is no significant difference between the proportions for male or female corresponding authors in all of the plots on the top line for female reviewers – suggesting that the recommendations of female reviewers differ less with corresponding author gender than those of male reviewers.

## (F) Gender characteristics of final manuscript outcome

In Section D3 we saw that most submissions have more than one reviewer for each revision (2 or 3 were most common). We have analysed the individual reviewer recommendations and although each review is a large influence on the final outcome of the submission, the editor must evaluate and consider all reviews when coming to a final decision about the outcome of the submission. Here we will consider gender characteristics of the final outcome of the article.

### (F1) Relationship between final outcome of original submission and author gender

We traced through all of the revisions that were submitted for each original submission in order to evaluate the final outcome resulting from each original submission within the data time period. There are four such possible outcomes of a submission – most (380 714, 53.1%) are rejected (including those that are rejected without peer review), some are accepted (327 688, 45.7%), some are undecided (4,162, 0.6%) and some have a status of “revise” (506, 0.07%). Most of the latter two cases are for submissions that had not reached their final revision before the end cut-off date of the data interrogated which is why their numbers are low. The percentage of original submissions from female corresponding authors for each these outcomes are 24.9% *rejected*, 22.9% *accepted*, 23.9% *undecided* and 22.8% *revise* (full breakdown figures are in Table F1a of ESI for corresponding authors and Table F1b† for first authors).

The percentage of original submissions from female corresponding authors that are ultimately accepted, at 22.9%, is very slightly, but significantly (*p* = 1.25 × 10^–14^) below that of all submissions. Conversely, the percentage of original submissions from female corresponding authors that are ultimately rejected is very slightly, but significantly (*p* = 3.21 × 10^–16^) higher than that of all submissions (23.9%). The small sample sizes for undecided and submission for revision mean that any difference from the baseline is not significant (*p* = 1.00 × 10 for both). When considering submissions from first authors rather than corresponding authors, there is no significant difference between any of these outcomes and the baseline.

These percentages of female authors who have accepted submissions are broadly in line with corresponding numbers for Chemistry articles in PubMed and arXiv of 20.4–21.0% for last authors and 34.8–35.4% for first authors.^14^

Expressing the outcome a different way, the success rate for acceptance of all submissions for female corresponding authors is 47.38% and for male corresponding authors it is 50.1% (Chisq test of independence of success frequency with gender is significant with *χ*^2^ (1, *n* = 310 616) = 169.94, *p* = 7.64 × 10^–39^).

For female first authors, the success rate is 48.0% and male first authors is 48.8% (Chisq test of independence of success frequency with gender is significant with *χ*^2^ (1, *n* = 317 710) = 18.97, *p* = 1.32 × 10^–5^).

### (F2) Relationship between agreement between reviewer recommendations and final revision status, reviewer gender, corresponding author gender, editor gender

We have investigated the reviewer recommendations and their gender characterisations previously in Section E and saw in particular that submissions from female corresponding authors were more likely to receive more negative review decisions and less likely to receive positive review decisions (particularly from male reviewers as in Section E3). We have also seen in Section F1 that this translates through to a slightly lower percentage of submissions from female corresponding authors being ultimately accepted, and a higher proportion being ultimately rejected. We will now consider the editor decision that links them, and in particular how likely it is to agree with the separate reviewer recommendations. For each headline accept/reject reviewer recommendation (where minor and major recommendations are included in the headline “accept” recommendation) we have evaluated a new variable “status and recommendation agree” with values dependent on the reviewer recommendation and final status of the revision. If the reviewer major recommendation of reject or accept matches the final status of revision, “status and recommendation agree” takes the value “Agree”. The final status “revise” is defined as agreeing with reviewer major recommendation “accept”. Other combinations are mapped onto a “Disagree” value for “status and recommendation agree”.

We use this dependent variable that is generated for each reviewer recommendation to investigate whether it differs with reviewer gender. The full set of figures for the breakdown is given in Table F2a of the ESI,† but to summarise, the female percentage of reviewers where the status and reviewer recommendation agree is 20.9% and that for reviews where the status and reviewer recommendation disagree is 20.2% (Chisq test of independence is significant with *χ*^2^ (1, *n* = 456 710) = 20.59, *p* = 5.70 × 10^–6^). There is a small but significant difference between the level of agreement for female and male reviewers – editors are slightly more likely to decide on final statuses that agree with the recommendations of female reviewers than male reviewers.

**Fig. 20:**
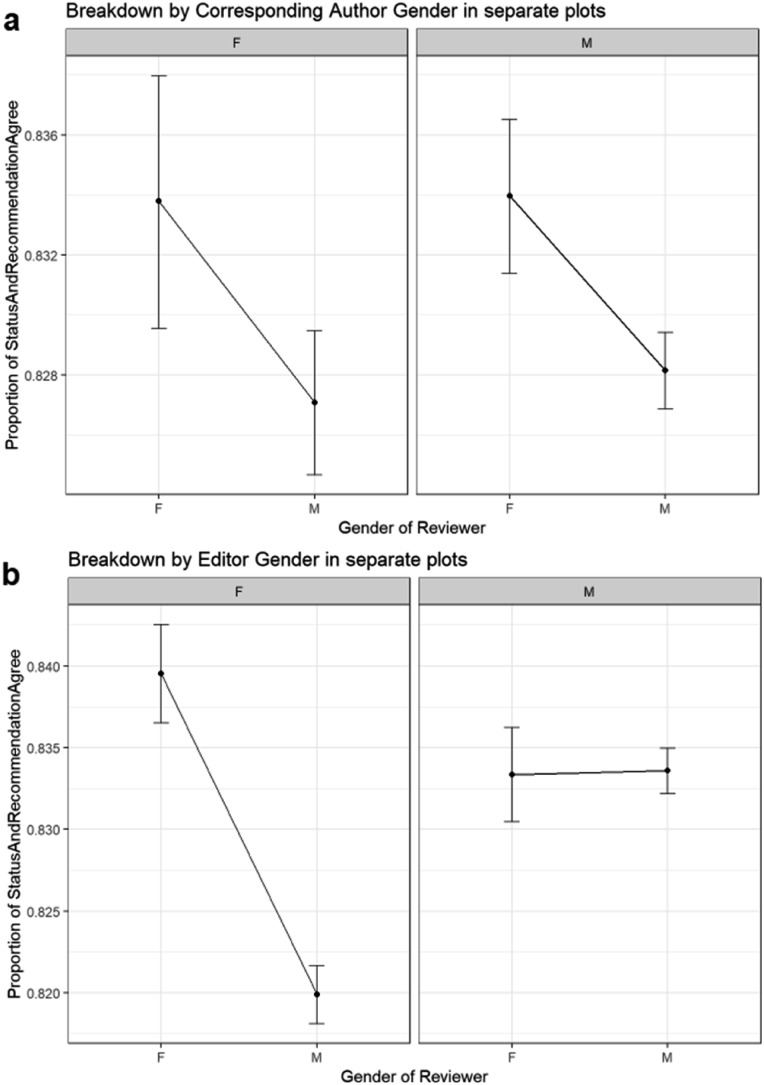
Binomial GLM models of “Status and recommendation agree” variable and reviewer gender. (a) – Binomial GLM model of “Status and recommendation agree” variable, reviewer gender and corresponding author gender (Model: StatusAndRecommendationAgree ∼ ReviewerGender * CorrAuthorGender). ANOVA Pr(>Chi): ReviewerGender is significant (0.0018); CorrAuthorGender is not significant (0.643); ReviewerGender: CorrAuthorGender is not significant (0.843). (b) – Binomial GLM model of “Status And Recommendation Agree” variable, reviewer gender and editor gender (Model: StatusAndRecommendationAgree ∼ ReviewerGender * EditorGender). ANOVA Pr(>Chi): ReviewerGender is highly significant (1.09 × 10^–5^); EditorGender is highly significant (1.05 × 10^–9^); ReviewerGender: EditorGender is highly significant (1.42 × 10^–7^).

The effect of an additional variable of corresponding author gender were again modelled using a logistic GLM – the effects are shown in Fig. 20a.

There is no significant relationship between the “status and recommendation agree” variable and the corresponding author gender, and no significant interactions between corresponding author gender with this outcome.

In contrast, including the interactions of an additional variable of editor gender rather than corresponding author gender in the binomial GLM model shown in Fig. 20b is significant.

The *p*-value of the modelling of the relationship between the outcome variable of “status and recommendation agree” and the interaction of editor gender and reviewer gender shows that the relationship is significant. Female editors agree with female reviewers significantly more than male reviewers. For male editors there is no significant difference between the proportions of reviews when they agree with male reviewers compared to female reviewers.

### (F3) Female percentage of accepted submissions by chemistry sub-discipline and impact factor

**Fig. 21:**
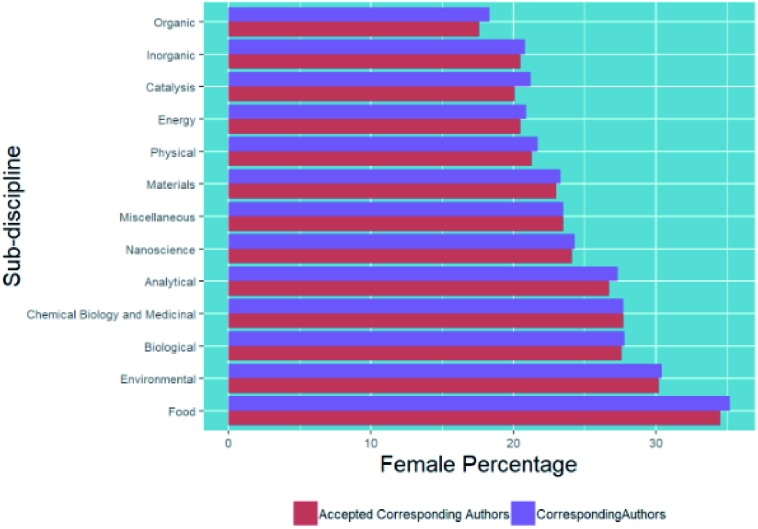
Percentage breakdown of accepted submissions by corresponding author gender and chemistry sub-discipline in comparison to that of all original submissions.

In Section B6 we showed how the gender of corresponding authors of original submissions vary with chemistry sub-discipline, and here we investigate whether the subsections of each of these which is accepted for publication differs significantly in Fig. 21. The analogous plot for first authors is in Fig. F3b of the ESI.†

For all chemistry sub-disciplines, the female percentage of corresponding authors of accepted submissions is less than that of original submissions (indicating that more submissions from female corresponding authors are rejected between these stages). This is not the case for female first authors where for 6 of the 13 high level subjects, the female percentage of accepted submissions is higher than that of original submissions which indicates that first authors don't experience the same level of gender imbalance.

*Catalysis* is the sub-discipline with the greatest decrease in female percentage of corresponding authors of accepted submissions *versus* all original submissions with a difference of 1.1%, but *Organic*, and *Food* are slightly lower at 0.7% and *Miscellaneous*, and *Analytical* at 0.6%. All three are sub-disciplines that had a much smaller percentage of female reviewers than corresponding authors did in Section D4. In contrast, there is no difference between the female percentage of corresponding authors for submissions and acceptances for *Chemical Biology and Medicinal* and only a small difference of 0.2% for *Biological*, *Nanoscience* and *Environmental*, but it is worth noting that these are subjects with considerably fewer female reviewers than corresponding authors in Section D4.

**Fig. 22:**
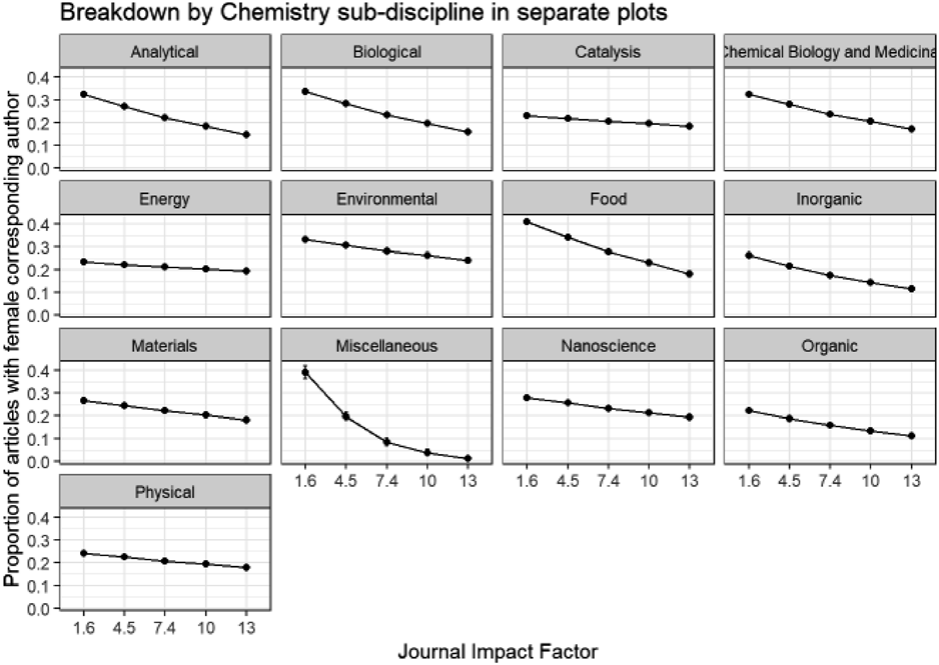
Binomial GLM model of corresponding author gender of accepted submissions, chemistry sub-discipline and journal impact factor (Model: CorrAuthorGender ∼ Category * ImpactFactor). ANOVA Pr(>Chi): Category is highly significant (0.00 × 10^0^); ImpactFactor is highly significant (0.00 × 10^0^); Category: ImpactFactor interactions are highly significant (3.18 × 10^–73^).

Similarly, we can revisit our analysis in Section B6, in which we investigated how the gender of authors varied across impact factor within the different sub-disciplines. Now we can consider just the subset of these submissions that were accepted. The effects of the resulting GLM model is shown in Fig. 22 for corresponding authors and the analogous plots for first authors are shown in Fig. F3d of the ESI.†

We see the same trends of female authorship for published articles as for submitted articles – the review process does not disrupt this, and there is still a drop off in the percentage of female corresponding and first authors of journals with a higher impact factor for all chemistry sub-disciplines.

This is in line with previous studies which also found that a negative correlation between the Impact Factor of journals (standardised by discipline) by the proportion of women authors^14^ and also in the field of neuroscience publications.^31^ This has important implications for the careers of female researchers since impact factor of journals of publications is used as a proxy for their excellence and impact, and so the lower submission rates of female authors to higher impact journals that leads to it is a point for concern.

### (F4) Female percentage of accepted submissions by final number of revisions

**Fig. 23:**
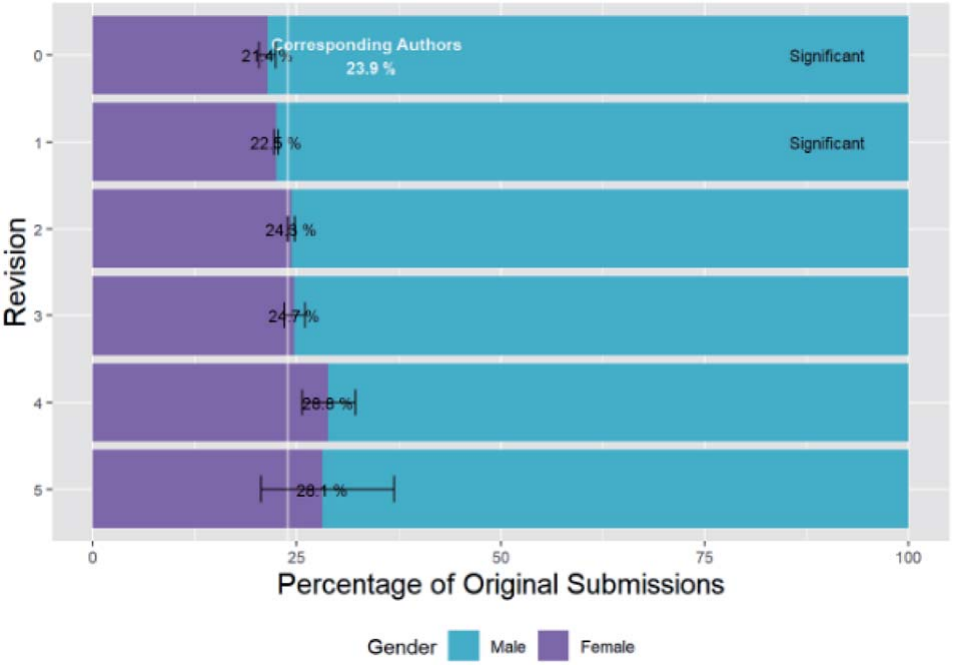
Percentage breakdown of submissions by corresponding author gender and final number of revisions.

In Fig. 23 we can see the breakdown of the number of revisions of accepted submissions *versus* corresponding author gender, and the analogous figure for first authors in Fig. F4b of the ESI.†

Fig. 23 shows that female-authored papers take more revisions to get to final publication than male. For corresponding authors there is a slight but significant increase in the female corresponding authorship of submissions with increasing number of revisions. These findings are in line with our observation in Section E2 that reviewers are more likely to recommend major revisions (which will result in another revision for review being necessary) for female corresponding authors than male, and less likely to recommend acceptance or minor revisions (which do not result in another revision for review being necessary) for female corresponding authors than male. The mean number of revisions for accepted submissions from female corresponding author (1.27, standard error = 0.003, *n* = 35 206) is significantly higher than the equivalent for male corresponding authors (1.24, standard error = 0.002, *n* = 118443) (two sample *t*-test, *p* = 7.30 × 10^–18^).

We also looked at the time from initial submission to final editor decision for accepted submissions, and the mean number of days for female corresponding authors (17.81, standard error = 0.169, *n* = 43 194) is significantly greater than for male corresponding authors (16.35, standard error = 0.089, *n* = 134913) (two sample *t*-test, *p* = 2.85 × 10^–14^).

These observations for female corresponding authors do not apply to first authors – while there is a slight increase in the female first authorship of submissions with increasing number of revisions, this is not significant.

## (G) Gender characteristics of citations

Article publication is not the end of the story. Community interest and uptake is important if the article is to further the careers of the researchers who wrote it. While it is not the only, or necessarily the best way, the number of citations of a published article has an impact on the future career of the researcher. As such, here we investigate the effect of gender on citation behaviour.

Analyses in all parts of this Section G are based on 141 073 citations from RSC articles to RSC articles (no other cited or citing articles from other publishers are considered) from August 2011 until September 2018.

Here we focus on corresponding authors rather than first authors since the trends are the same for both but the differences larger for corresponding authors.

The baseline we use for comparison in this section is the female percentage of corresponding authors of cited articles shown above, 18.4%.

### (G1) Overview of citations by gender

**Fig. 24:**
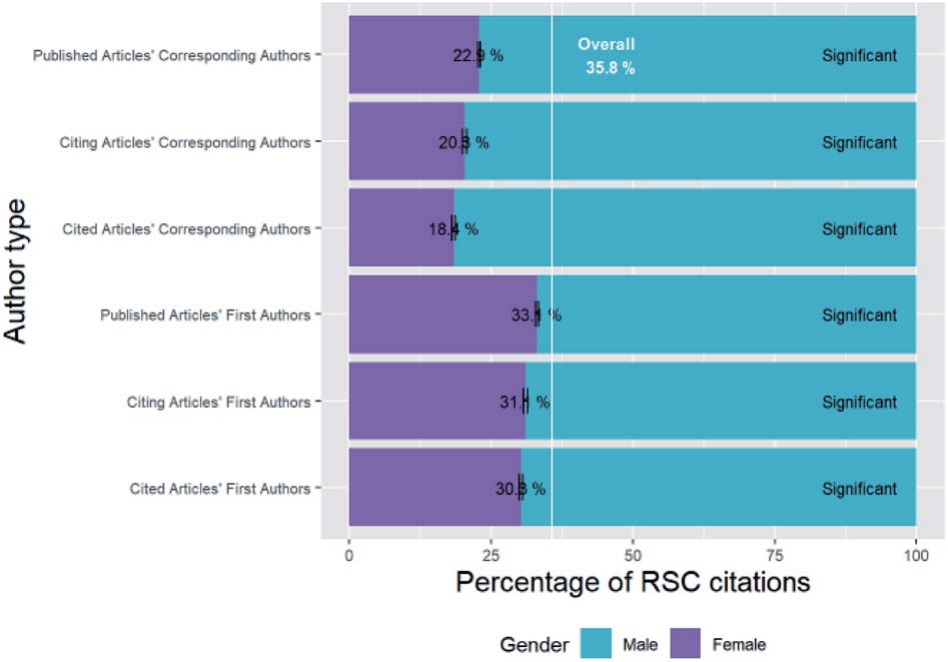
Gender percentage breakdown of corresponding and first authors of cited and citing articles of citations in comparison to those of all publications.

In Fig. 24 we show the female percentage of authors (corresponding and first authors) of all published articles *versus* those of citing and cited articles for citations between RSC articles.

The percentage of RSC articles that are authored by female corresponding authors and cite other RSC articles is lower than the average of all RSC articles that are accepted for publication (as described in Section F1) by female corresponding authors, and likewise for first authors.

It is apparent that the female percentage of citing articles' corresponding and first authors is less than that for published articles respectively, indicating that female authors cite less than male authors. Indeed, the mean number of citations from female-corresponding-authored articles (7.79, standard error = 0.073, *n* = 11 132) is significantly less than that of male-corresponding-authored articles (10.46, standard error = 0.089, *n* = 43 606) (two sample *t*-test, *p* = 1.95 × 10^–119^).

It is also evident from this data set that overall publications from female corresponding and first authors are cited less than those from male authors. This is corroborated by the mean number of RSC citations to female-authored RSC articles (5.6, standard error = 0.078, *n* = 10 906) being significantly less than that for male-authored RSC articles (7.18, standard error = 0.05, *n* = 48 379) (two sample *t*-test, *p* = 3.92 × 10^–64^). This is in contrast to Elsevier's analysis of citations in its publications^3^ that found that although women publish fewer papers than men do, there was no difference in the numbers of citations that they received.

We have also calculated the “success rate” of published articles which were cited by cross-referencing the articles which were found to have been published between 2014 to 2018 (from Section F) with the cited articles from this data set (by matching DOIs) so as to flag the “successes” as those which have been cited at least once by another RSC article. As such, the success rate for female corresponding authors is 5.25% and for male corresponding authors it is 6.79% (Chisq test of independence of success frequency with gender is significant with *χ*^2^ (1, *n* = 242 281) = 174.17, *p* = 9.08 × 10^–40^). Similarly, for female first authors the success rate is 5.56% and for male corresponding authors it is 6.57% (Chisq test of independence of success frequency with gender is significant with *χ*^2^ (1, *n* = 248 946) = 98.29, *p* = 3.61 × 10^–23^). These success rates are low because citations build up over time and these publications are relatively recent, but a gender disparity is evident even on these timescales.

**Fig. 25:**
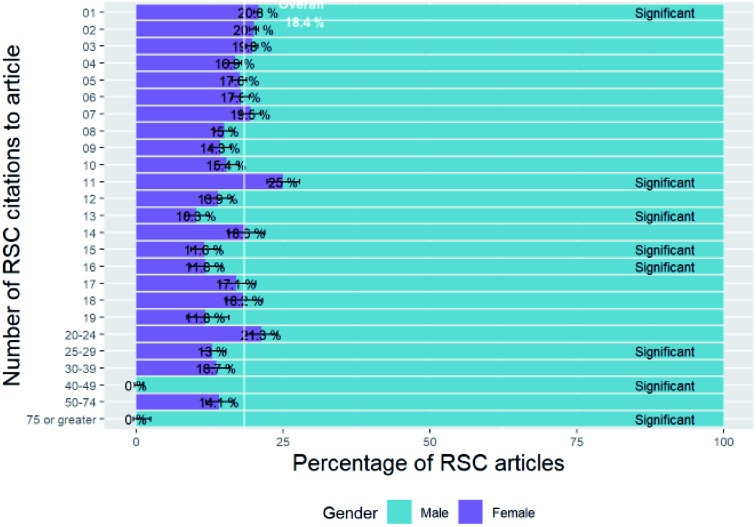
Percentage breakdown of articles' corresponding author gender by number of RSC citations to it.

Fig. 25 breaks down all cited RSC articles by the number of citations from other RSC articles to it.

For articles with less than 10 citations, a progression to higher percentages of male authorship with increasing number of citations can be seen, and the bottom of the plot (which corresponds to highly cited articles) shows higher male authorship. For articles with citations greater than 10 there is no clear trend and small sample sizes are common particularly for the articles with large numbers of citations.

### (G2) Citation success of published article by whether they were unanimously accepted and corresponding author gender

**Fig. 26:**
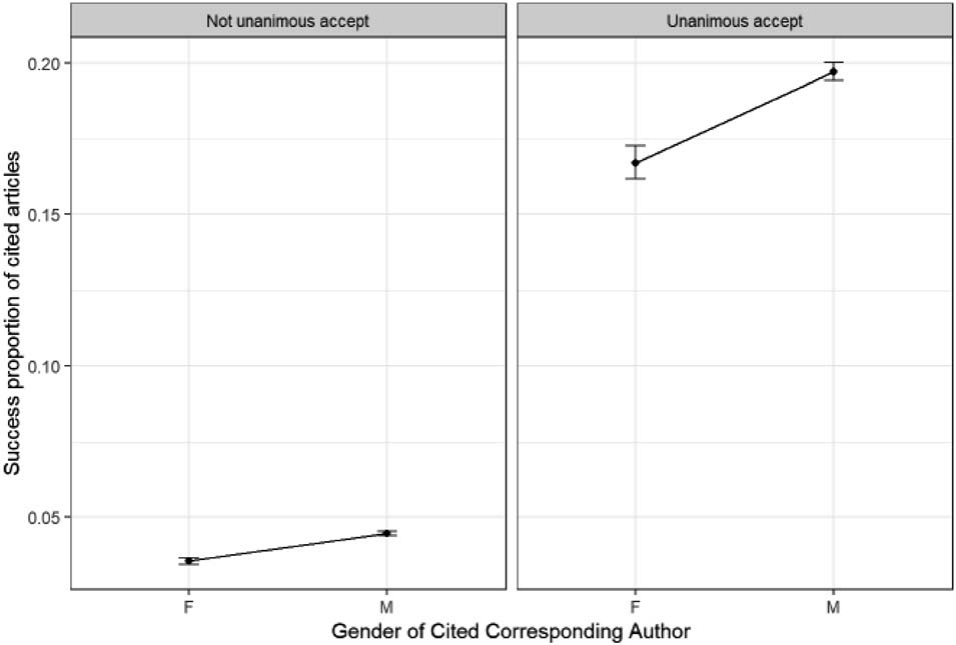
Binomial GLM model of citation success of published articles and corresponding author gender controlled by whether the article was unanimously accepted in its first revision (Model: Cited ∼ UnanimousAccept * CorrAuthGender). ANOVA Pr(>Chi): UnanimousAccept is significant (0.00); CorrAuthorGender is significant (3.22 × 10^–27^); UnanimousAccept: CorrAuthorGender interactions is not significant (0.408).

Here we investigate whether citation success varies with whether the submission was unanimously accepted in its first revision (and we include the “minor revisions” recommendation to be accepted, but not “major revisions”). Fig. 26 shows a GLM model which this time shows the proportion of articles in the original submission data set (of all submissions between 2014 to 2018) that were cited at least once by another RSC article in the citations set of data, varying with corresponding author gender and whether the article was unanimously accepted in its first revision.

The lower citation success rate of articles which were not unanimously accepted (left-hand plot) compared to those that were (right-hand plot) is evident from the ANOVA *p*-value. For both, the proportion of articles published by female corresponding authors have lower citation success proportions than those by male corresponding authors, and this difference is also significant. However, while both variables are significant, the interaction between them is not significant, which indicates that female corresponding authors are cited less than their male counterparts in both sets of articles.

### (G3) Female percentage of citations by date

**Fig. 27:**
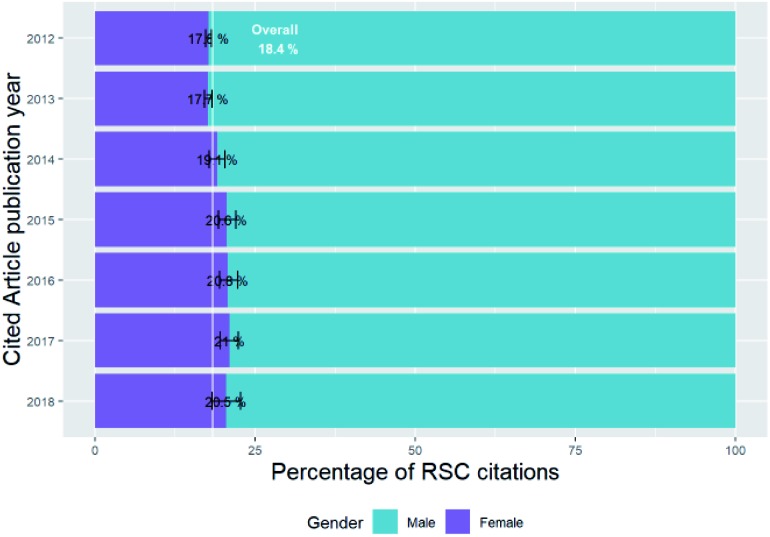
Percentage breakdown of cited corresponding author gender of citations by the cited article's publication year.

We now investigate how citations to female authors changes over time (according to the date of the cited article) in Fig. 27.

There is a slight increase of the percentage of articles which cite articles from female corresponding authors over the time period since 2012 (discounting years where the sample size was too small to tell). However, for no year is the female percentage significantly different from the baseline of the average for citations.

### (G4) Self-citations by gender

**Fig. 28:**
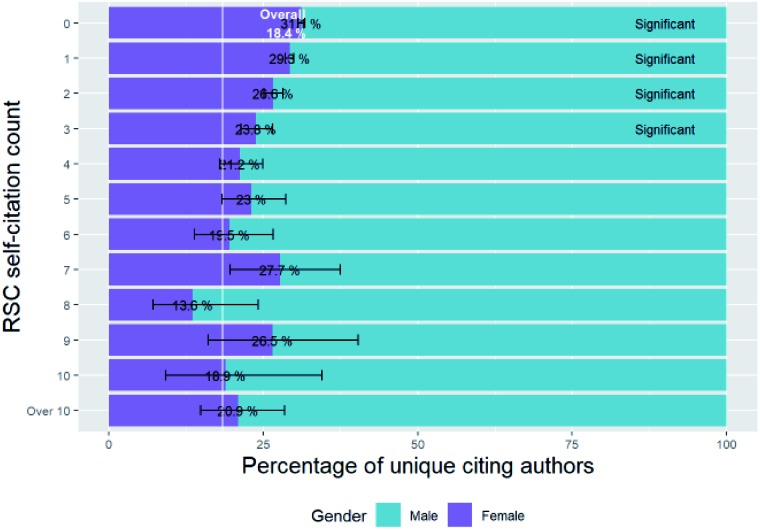
Breakdown of gender of de-duplicated authors of all citing articles and number of self-citations in RSC citation data set.

We investigated whether there are any significant differences between self-citation behaviour between articles in RSC journals according to gender by looking at all unique (by first name and last name) authors of all citing manuscripts in the RSC citation data set and counted the number of times that they cited a manuscript of which they were an author (by matching first name and last name). If multiple authors are self-cited in the same citing-article-cited-article pair each self-citation for each author is counted. In Fig. 28 we show the gender breakdown of RSC self-citation counts.

Authors with no self-citations show a higher female percentage than those with one or more self-citations, and there is a slight, but significant, trend of decreasing female percentage with increasing self-citation count. The mean number of self-citations for female authors (0.6, standard error = 0.011, *n* = 15 175) is significantly lower than that for male authors (0.71, standard error = 0.007, *n* = 35 658) (two sample *t*-test, *p* = 7.335 × 10^–17^). The overall percentage of authors who self-cited was 44.0%.

### (G5) Relationship between cited corresponding author gender and citing corresponding author gender

We now investigate whether female corresponding authors are more likely to cite other female corresponding authors. We have filtered out direct self-citations of the same cited and citing corresponding author so that results will show whether female corresponding authors cite *other* female corresponding authors more or less than male corresponding authors (and *vice versa*). The full set of figures for the breakdown is given in Table G5 of the ESI,† but to summarise, the female percentage of cited corresponding authors for female citing corresponding authors is 20.6% but that for male citing corresponding authors is 17.8% (Chisq test of independence of is significant with *χ*^2^ (1, *n* = 23 432) = 19.2, *p* = 1.16 × 10^–5^). We can see that the percentage of female corresponding authors cited by other female corresponding authors is higher than that for male corresponding authors. This effect is small but significant.

### (G6) Female percentage of citations by chemistry sub-discipline and impact factor

**Fig. 29:**
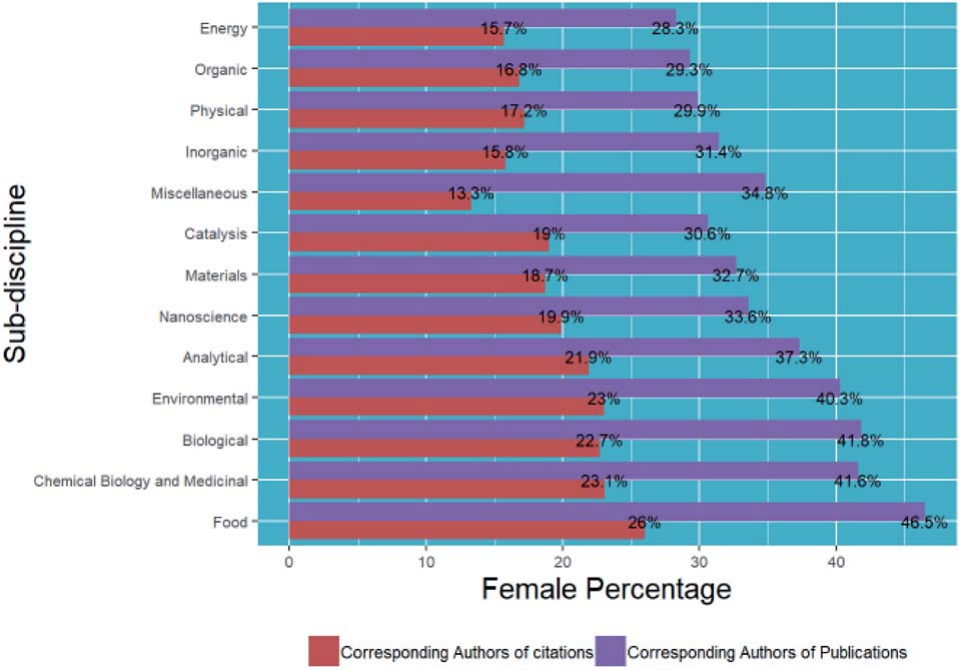
Gender percentage breakdown of cited corresponding authors of citations by chemistry sub-discipline in comparison to that of corresponding authors of all accepted submissions.

Each cited article has been categorised into relevant chemistry sub-disciplines as described in the methodology. The female percentage of cited authors of citations in each sub-discipline is shown in Fig. 29 alongside the female percentage of corresponding authors of all accepted articles in that chemistry sub-discipline.

The distribution by chemistry sub-discipline is in general consistent with trends shown previously – sub-disciplines with higher female percentages of corresponding authors of published articles are those with higher percentages of citations of those. Categories in which female under-citing might be occurring (with the biggest differences in percentages for cited and published corresponding authors) are *Miscellaneous* (22.9% difference), *Food* (20.6% difference), *Biological* (19.1% difference), *Chemical Biology and Medicinal* (18.5% difference), *Environmental* (17.2% difference).

**Fig. 30:**
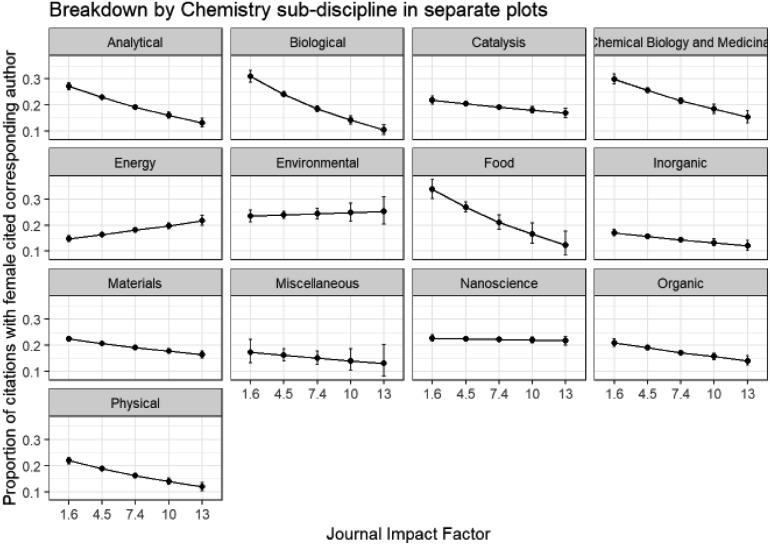
Binomial GLM model of corresponding author gender of citations, chemistry sub-discipline and journal impact factor (Model: CitedCorrAuthorGender ∼ Category * ImpactFactor). ANOVA Pr(>Chi): category is highly significant (3.26 × 10^–66^); ImpactFactor is highly significant (1.96 × 10^–17^); category: ImpactFactor interactions are highly significant (2.49 × 10^–14^).

In Fig. 30 we show a binomial model of female proportion of citations with impact factor broken down by category.

For the majority of Chemistry sub-disciplines, there is a decrease in the percentage of citations to articles with female corresponding authors with increasing impact factor. The exceptions are Energy, which shows the opposite trend, and Environmental and Nanoscience show no marked increase or decrease with female corresponding author citation percentage with impact factor.

## (H) Gender characteristics of H-index

We have seen small but significant gender-based differences during the publication process over the limited time range under investigation. We now turn out attention to consider the combined effect over time of these differences on H-index,^32^ as a quantitative measure of research career success (although concerns have been expressed about its appropriateness and fairness^33^). To study this we used the “H-index ranking of living chemists”^34^ – compiled by Henry Schaefer, of the University of Georgia, US, together with colleague Amy Peterson and published online by Chemistry World. They assessed the H-index of around 2000 chemists to identify the list that includes only those with highest scores of 55 or greater. Note that this list and the H-index values have not been updated since 2011, and not all of the chemists in the list are still living.

**Fig. 31:**
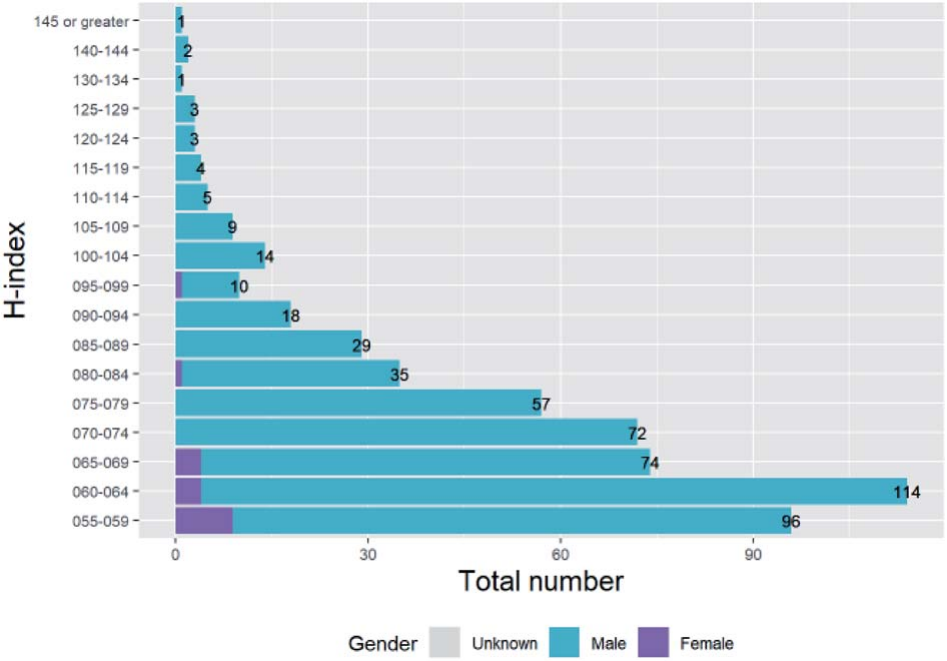
Total breakdown of living Chemists with highest H-index ranking by gender and H-index.

We have taken this list, and expanded the first initial to a name (using Wikipedia and Google Scholarly API lookups or manual lookups if neither of these returned anything) and run these first names through our gender mapping script to assign gender of each. Because of the small sample size, and small number of females that emerged, thorough manual checking and adjustment was performed on the genders that were obtained. The total numbers for each H-index band are shown in Fig. 31.

Only 19 female authors appear in the list of 547 chemistry researchers – a percentage of 3.5%, which is significantly different from the baseline female percentage of chemistry researchers overall 35.8%.

We should, however, note that H-index has been criticised as an unfair measure of publication success. H-Index values vary greatly with chemistry sub-discipline, favouring sub-disciplines that are more prolific in terms of publications and citations at the expense of those that produce fewer publications. Also, by its nature, H-index ranking favours those researchers who are further on in their career and in years. To investigate this we have also obtained the years of birth of these chemists where available from Wikipedia and internet searching and found that the mean age of female chemists when the original analysis was conducted in 2011 was 66.1 (standard error = 4.06, *n* = 16) and for male chemists was 69.3 (standard error = 0.53, *n* = 403), the difference between which is not significant (two sample *t*-test, *p* = 0.44). However, the main salient point from considering the ages of these chemists is that, as might be expected, they correspond to an age and career stage where females are underrepresented (due to the “leaky pipeline”) even before the number of publications and citations are considered. Reflection of the gender balance of a previous generation of chemists may also be exacerbated by these H-index values and their rankings being from 2011 rather than more current.

However, even given all of these factors, we have highlighted that using the H-index in its simplest form as a measure of publication and career success results in dramatic underrepresentation of female researchers. Using a modified form (for example weighting by length of career) might address this to some extent.

## Conclusions

We have conducted a thorough analysis of data available to us as the Royal Society of Chemistry, and as publishers of the chemical sciences, in order to identify gender imbalances.

We are releasing code, tools and data with this paper for others to use in their analysis. The work described in this paper is only the start in terms of studying gender bias in the chemical sciences. We are planning to extend this work to ascertain reasons for the imbalances we see. We plan to explore further the imbalances we observed by investigating: improving the gender assignment methods to get a more inclusive picture of the differences; patterns of behaviour (*e.g.* cliques); and differences in review styles (*e.g.* sentiment).

The female percentages quoted are percentages relative to those with known gender (omitting those whose gender could not be deduced). This means that the analysis considers 53% of corresponding authors, 48% of first authors, 57% of reviewers, but 74% of editors, and as discussed in Section B3, it was apparent that those not included are predominantly of Asian origin, even when an alternative gender-assignment method and name-gender mapping data set were used. For parts of this analysis which focus on characteristics of authors, reviewers and editors this is an unfortunate but somewhat unavoidable limitation in the scope of this study that we should highlight. When considering potential conscious and unconscious biases towards authors and reviewers when it comes to rejection without peer review, reviewer decision, editor decision and citations, these biases will be stronger towards or against those researchers whose names are more readily associated with a particular gender. This paper therefore focuses on the effects of more inter-Westernised gender biases, rather than those that might exist in non-Westernised cultures or between Westernised and non-Westernised cultures, the latter of which would additionally be complicated by geographical biases.

**Fig. 32:**
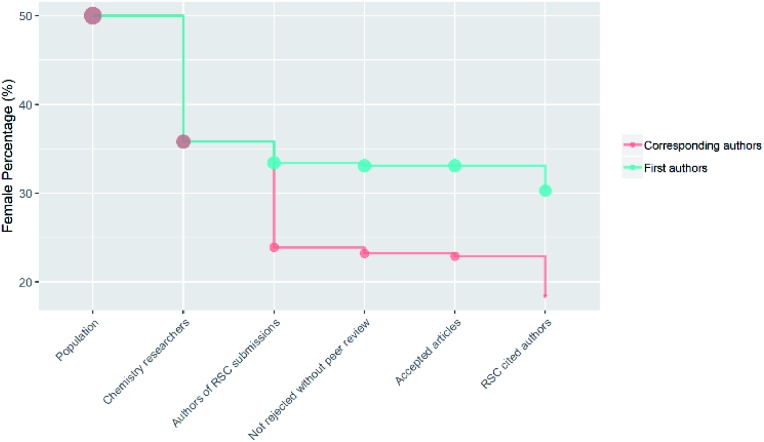
Female percentage of corresponding authors throughout publication process.

In Section A we looked at 3 different data sets to establish a baseline of 35.8% of chemistry researchers that are female. As demonstrated in Fig. 32, at each step of publication we see small but significant drops in the female percentage of authors that gradually decrease from this baseline. Rather than one dominant factor, it is more akin to “death by a thousand cuts”. This female drop off through the publication process is most marked for female corresponding authors, but is also apparent to a lesser degree for female first authors. The cumulative pattern by the end of the publication process investigated here is seen in Fig. 32. The same trends were also apparent when considering the complementary technique of success rates as a function of gender through these various publication stages. The female corresponding author success rates were consistently significantly less than the male corresponding author success rates: for submissions progressing through to peer review (69.31% female, 71.98% male); submissions being accepted for publication (47.38% female, 50.1% male); and publications being cited at least once by another RSC article (5.25% female, 6.79%). A similar trend was apparent for first authors' success rates to a lesser extent. Each step of Fig. 32 was broken down and investigated in more detail through the sections of this article.

In Section B, we saw that female authors submit fewer articles (Section B2), are less likely to author a paper on their own (Section B4) and are more likely to submit to journals with a lower impact factor within a particular chemistry sub-discipline (Section B6) than their male counterparts. Female first authors are more likely to co-author with female corresponding authors than male corresponding authors and the converse is true (Section B4).

Articles authored by female corresponding and first authors are more likely to be rejected without review than those from male corresponding authors (Section C2). While there is a higher percentage of female editors than the baseline for Chemistry researchers (Section C1) (due largely to the gender balance of in-house RSC staff), female reviewers are under-represented, reflecting the female percentage of invitations rather than a tendency to accept those invitations less or decline them more (Section D1). Female reviewers are more likely to review papers that have been under revision many times *e.g.* revisions 3, 4, and 5 (Section D3). Female reviewers are more likely to review submissions from female corresponding authors (D2) and when they do, they are more likely to accept or recommend minor revisions for these female corresponding authors than their male counterparts (Section E3). However, though, in general, reviewers are more likely to recommend rejection or major revisions rather than acceptance or minor revisions for submissions from female corresponding authors than male ones (Section E1). Female reviewers are more likely to recommend major revisions rather than rejection for submissions that they review (Section E2).

These reviewer recommendations add up to slightly (but significantly) fewer submissions from corresponding authors being accepted for publication than those of their male counterparts (Section F1) although this is not significant for female first authors compared to male first authors (Section F1). Editors are slightly but significantly more likely to choose a final status of a revision which agrees with female reviewers than male reviewers (Section F2) and this difference increases more in cases where the editor is female (Section F2). There is a slightly but significantly higher female percentage of corresponding authors of submissions with increasing number of revisions (Section F4). The mean time from submission to final decision for accepted submissions from female corresponding authors is significantly greater than that from male corresponding authors (Section F4). The proportion of female corresponding and first authors of accepted articles for higher impact journals within a sub-discipline are lower than that for lower impact journals (Section F6), following the similar trend for submissions by impact factor and gender (Section B6), and with important implications to potentially limit the impact of the research and careers of these female authors.

Published articles from female corresponding and first authors (to a lesser extent) receive fewer citations than those from their male counterparts (Section G1). Female corresponding authors cite less articles than male corresponding authors (Section G1). Publications with female corresponding authors are less likely to be highly cited than those of their male counterparts (Section G1). Articles that were unanimously accepted by reviewers were proportionally cited less for female corresponding authors than those by male corresponding authors (G2). Male authors self-cite more than their female counterparts (Section G4). Female corresponding authors are more likely to cite other female corresponding authors and the converse is true (Section G5). There is a decrease of the percentage of female corresponding authors of citations with impact factor for the majority (but not all) of Chemistry sub-disciplines (G6).

In summary, we do not see one dominant factor disadvantaging female researchers in the publishing process, but a series of small but significant results. We did, however, observe some evidence of gender homophily (between authors, and their reviewers and editors) – with female researchers working together to counteract these imbalances.

There are some differences within Chemistry sub-disciplines, but those which are strongest in female corresponding and first authorship of submissions and publications – *Food*, *Environmental*, *Biological* and *Chemical Biology and Medicinal* – (Sections B6 and F3) are also those which are most under-represented by female reviewers (Section D4) and under-cited for female corresponding authors (Section G6). The sub-disciplines with lower female authorship are *Organic*, *Catalysis*, *Inorganic* and *Energy* (Section B6 and F3), but *Inorganic* is the only sub-discipline which has a higher percentage of reviewers than that of submitting corresponding authors (Section D4).

There is no significant change in the female proportion of corresponding or first authors of submissions (Section B5) or reviewers (Section D3) over the 3 year period investigated, or the number of citations to publications by female corresponding authors over time from 2012 till 2018 (Section G3).

In Section H, a marked under-representation of female researchers was evident in the living chemists with the highest H-index scores.

We have provided evidence that there are compounding disparities between male chemists and their female counterparts through the publication process and have characterised these as far as possible. Several differences identified in this paper (lower likelihood of publications being accepted, increased number of revisions and therefore time until publication) could be interpreted as being due to lower productivity at fixed evaluation points (such as recruitment, grant proposal review or promotion). An underlying question is whether this gender difference is due to (1) gender bias, (2) a difference in the research quality according to author gender or (3) other systematic differences in the submission features of male and female co-authors that might affect publication success *e.g.* impact of journal or number of authors. Cause 2 (whether submission quality varies with gender) is somewhat debateable and controversial, but research quality could be affected by factors such as lower research grant funding being awarded to female principal investigators^35^ or female authors holding themselves to higher standards,^36^ or indeed the “leaky pipeline” itself acting as a circular cause and effect of the publication disparities. We have provided breakdowns by publication features to address cause 3, but it is hard to disentangle causes 1 and 2 when quality of submissions is judged by the peer review that is under investigation. We have had little success in identifying a control to act as a proxy for quality that could be used to uncouple causes 1 and 2 and are reluctant to judge the quality of a submission by a proxy external to the article itself. As such the consistently lower female proportions of successful submissions in all groups that we observed should be considered in this wider context.

In an accompanying report^37^ we follow these observations up by highlighting specific points for action by the Royal Society of Chemistry and its community.

## Availability of materials and R code

R code examples and partial datasets for this project are available for download at: https://bitbucket.org/rscapplications/genderdiversity/src/master/.

## Conflicts of interest

Aileen Day, Peter Corbett and John Boyle are employees of the Royal Society of Chemistry (RSC), however they were not involved in, or had access to information on, the peer review selection, evaluation and decision process for this article. No RSC staff members were involved in the editorial decision taken on this manuscript, which was taken by *Chemical Science* Associate Editor, Alan Aspuru-Guzik. In the interests of full transparency, the identities of the reviewers are published alongside the reviewer reports and authors' response accompanying this article as ESI.

## Supplementary Material

Peer review details

Supplementary information
