# Supplementary material for: Is there a gender gap in chemical sciences scholarly communication?
Source: Chem Sci. 2020 Jan 28;11(8):2277–301. doi: 10.1039/c9sc04090k (PMC7057853; doi:10.1039/c9sc04090k)
Supplement: Supplementary file 2 [file SC-011-C9SC04090K-s002.pdf]

Electronic Supplementary Material (ESI) for Chemical Science.
This journal is © The Royal Society of Chemistry 2020

Electronic Supplementary Information

Is there a gender gap in Chemical Sciences scholarly communications?

A. E. Day*[a], P. Corbett [a] and J. Boyle [a]

[a]Royal Society of Chemistry, Thomas Graham House (290), Science Park, Milton Road, Cambridge, CB4 0WF

Corresponding Author

*E-mail: daya@rsc.org (A.E.D.).

For every figure shown in the parent article, the corresponding numbers plotted, with percentages and confidence intervals, significances and p-values are given in the tables of this ESI numbered with the section number that the figure appears in e.g. numbers corresponding to Figures 3 and 4 in section B2 of the main article are displayed in Table B2a and B2b of the ESI.

Table ESI_1: RSC journals information

Journal Name	Journal Sercode	Impact factor (2017)[1]	Journal Editorial Model	Journal Chemistry sub-disciplines[2]
Analyst	an	3.864	AE	Analytical
Analytical Methods	ay	2.073	AE	Analytical
Biomaterials Science	bm	5.831	AE	Materials
Chemical Communications	cc	6.29	Hybrid	Multidisciplinary
CrystEngComm	ce	3.304	Hybrid	Materials
Physical Chemistry Chemical Physics	cp	3.906	Hybrid	Physical
Chemical Society Reviews	cs		AE (with in-house pre-submission assessment)	Multidisciplinary
Catalysis Science & Technology	cy	5.365	AE	Catalysis
Dalton Transactions	dt	4.099	Hybrid	Inorganic
Energy & Environmental Science	ee		In-house	Catalysis; Energy & Sustainability
Environmental Science: Processes & Impacts	em	2.491	AE (with in-house pre-screening)	Environmental
Environmental Science: Nano	en	6.087	Hybrid	Environmental; Nanoscience; Engineering
Environmental Science: Water Research & Technology	ew	3.649	AE	Environmental; Engineering
Faraday Discussions	fd	3.427	Other	Physical
Food & Function	fo	3.289	AE	Chemistry and Biology Interface
Green Chemistry	gc	8.586	Hybrid	Catalysis; Energy & Sustainability
Integrative Biology	ib	3.294	AE	Chemistry and Biology Interface
Journal of Analytical Atomic Spectrometry	ja	3.608	In-house	Analytical
Journal of Materials Chemistry	jm		Hybrid	Materials
Lab on a Chip	lc	5.995	Hybrid	Nanoscience; Engineering
Molecular BioSystems	mb		Hybrid	Chemistry and Biology Interface
MedChemComm	md	2.342	AE	Chemistry and Biology Interface
Molecular Systems Design & Engineering	me		In-house	Energy & Sustainability; Materials; Physical; Engineering
Materials Horizons	mh	13.183	In-house	Materials
Molecular Omics	mo		Hybrid	Chemistry and Biology Interface
Metallomics	mt	4.069	In-house	Chemistry and Biology Interface
Nanoscale Horizons	nh	9.391	In-house	Nanoscience
New Journal of Chemistry	nj	3.201	AE	Multidisciplinary
Natural Product Reports	np	11.406	In-house	Chemistry and Biology Interface
Nanoscale	nr	7.233	AE (with in-house pre-screening)	Nanoscience
Organic & Biomolecular Chemistry	ob	3.423	Hybrid	Chemistry and Biology Interface; Catalysis; Organic
Photochemical & Photobiological Sciences	pp	2.902	AE	Chemistry and Biology Interface; Physical
Polymer Chemistry	py	4.927	AE	Materials
Inorganic Chemistry Frontiers	qi	5.106	AE	Inorganic
Materials Chemistry Frontiers	qm		AE	Materials
Organic Chemistry Frontiers	qo	5.455	AE	Organic
RSC Advances	ra	2.936	AE	Multidisciplinary
Reaction Chemistry & Engineering	re	4.641	In-house	Catalysis; Engineering
Chemistry Education Research and Practice	rp	1.621	AE	Multidisciplinary
Chemical Science	sc	9.063	AE (with in-house pre-screening)	Multidisciplinary
Sustainable Energy & Fuels	se		AE	Catalysis; Energy & Sustainability
Soft Matter	sm	3.709	AE	Materials; Physical
Journal of Materials Chemistry A	ta	9.931	Hybrid	Energy & Sustainability; Materials
Journal of Materials Chemistry B	tb	4.776	Hybrid	Materials
Journal of Materials Chemistry C	tc	5.976	Hybrid	Materials
Toxicology Research	tx	1.89	AE	Chemistry and Biology Interface
Royal Society Open Science	-		AE	

[1] Impact factors based on 2017 Journal Citation Reports ® (Clarivate Analytics, June 2018)
[2] Journal Chemistry sub-disciplines based on http://www.rsc.org/journals-books-databases/about-journals/

Figure ESI_2[illegible]
categorisati[illegible]

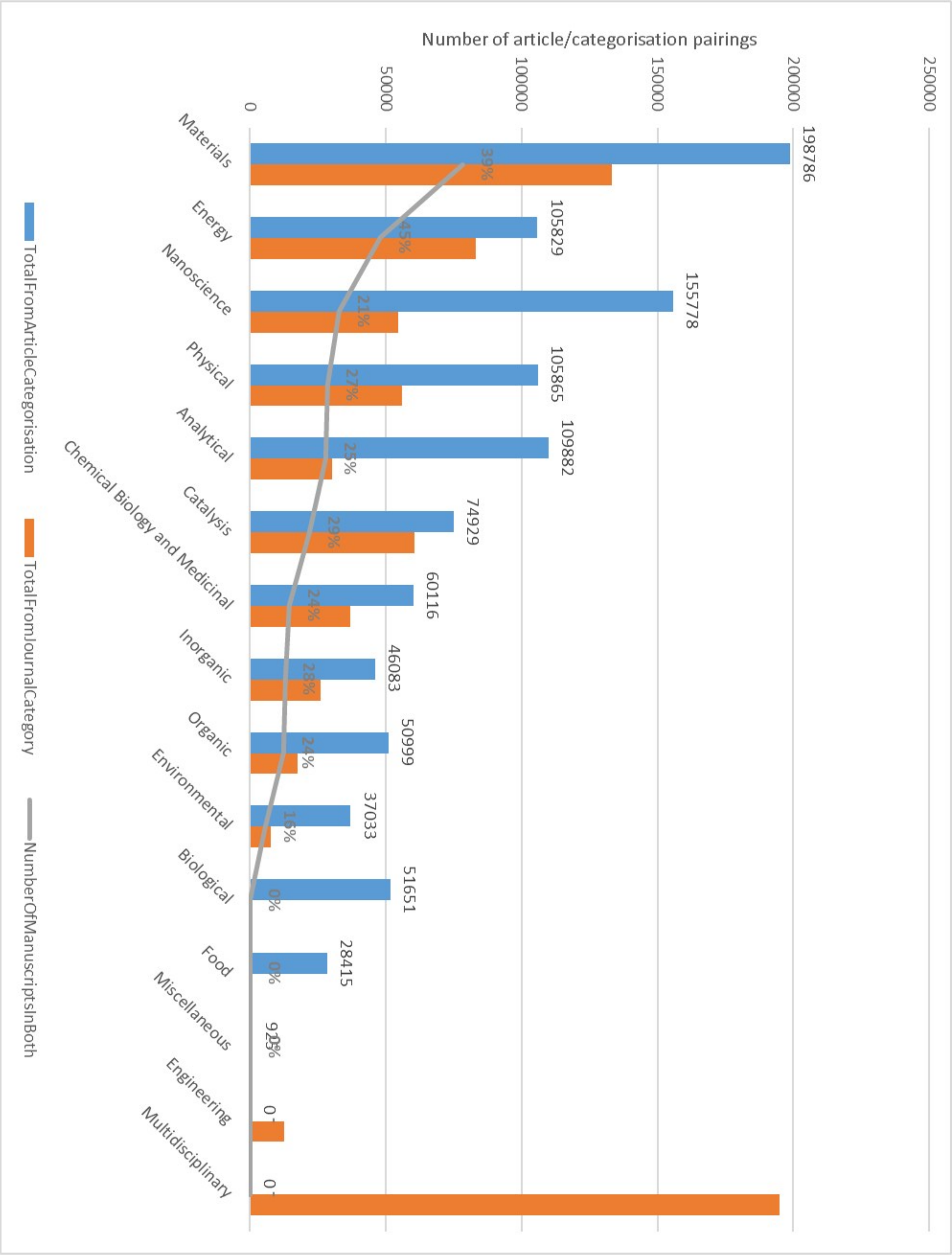

Table A1 (for Figure 1): Breakdown of chemistry researcher gender by HESA contract level[3]

HESA Contract Level	Female	Male	Unknown	Total For Level	Total Gender Deduced	Female Percentage	Male Percentage	LCI	UCI	LCI Count	UCI Count	Significance[4]	Adjusted P-Value
A0 to C2 Senior management	5	25	0	30	30	16.7	83.3	6.86	34.04	2.06	10.21		1.00e+00
D and E Head of Schools/ Senior Function head	10	60	0	70	70	14.3	85.7	7.75	24.54	5.43	17.17	Significant	2.41e-02
F1 Professor	65	540	0	605	605	10.7	89.3	8.51	13.48	51.47	81.53	Significant	4.59e-36
I0 Senior/principal lecturer, Reader, Principal Research fellow	105	410	0	515	515	20.4	79.6	17.13	24.09	88.20	124.05	Significant	2.44e-08
J0 Lecturer, Senior Lecturer, Senior Research Fellow	225	545	0	770	770	29.2	70.8	26.12	32.53	201.10	250.49		1.00e+00
K0 Lecturer, Research fellow, Senior research assistant, Teaching fellow	720	1475	0	2195	2195	32.8	67.2	30.87	34.79	677.58	763.74		1.00e+00
L0 Research assistant, Teaching assistant	320	545	0	865	865	37.0	63.0	33.84	40.26	292.72	348.27		1.00e+00
Doctorate Students	1660	2545	0	4205	4205	39.5	60.5	38.01	40.96	1598.31	1722.50	Significant	7.20e-12

[3] This publication contains data provided by the Higher Education Statistics Agency (HESA), based on the HESA Student and Staff record from 2015/16. Copyright: Higher Education Statistics Agency Limited. Neither the Higher Education Statistics Agency Limited nor HESA Services Limited can accept responsibility for any inferences or conclusions derived by third parties from data or other information supplied by HESA Services

[4] Significance calculated compared to baseline of average female percentage over all these contract levels (33.6%)

Table A2 (for Figure 2): Breakdown of chemistry researcher gender by RSC membership level

RSC membership level	Female	Male	Unknown	Total For Level	Total Gender Deduced	Female Percentage	Male Percentage	LCI	UCI	LCI Count	UCI Count	Significance[5]	Adjusted P-Value
Affiliate	389	632	366	1387	1021	38.1	61.9	35.17	41.12	359.09	419.82		1.00e+00
Affiliate Undergraduate	125	170	46	341	295	42.4	57.6	36.87	48.08	108.76	141.82		1.00e+00
Associate Member	988	1392	422	2802	2380	41.5	58.5	39.55	43.50	941.25	1035.40	Significant	2.04e-05
Fellow	297	1558	479	2334	1855	16.0	84.0	14.41	17.75	267.32	329.28	Significant	1.26e-76
Honorary Fellow	4	17	5	26	21	19.0	81.0	7.08	40.59	1.49	8.52		1.00e+00
Member	787	1838	508	3133	2625	30.0	70.0	28.26	31.76	741.78	833.76	Significant	9.01e-07

[5] Significance calculated compared to baseline of average female percentage over all membership levels (34.3%)

Table B2a (for Figure 3): Breakdown of corresponding author gender with number of submissions (numbers greater than 30 have been omitted)

Number of submissions	Female	Male	Unknown	Total For Level	Total Gender Deduced	Female Percentage	Male Percentage	LCI	UCI	LCI Count	UCI Count	Significance[6]	Adjusted P-Value
1	4413	8585	11798	24796	12998	34.0	66.0	33.14	34.77	4307.81	4519.42	Significant	9.63e-143
2	4622	9977	10354	24953	14599	31.7	68.3	30.91	32.42	4512.56	4732.85	Significant	2.08e-96
3	2517	5850	6542	14909	8367	30.1	69.9	29.11	31.07	2435.56	2599.97	Significant	3.38e-34
4	1642	3924	4290	9856	5566	29.5	70.5	28.32	30.71	1576.12	1709.46	Significant	5.96e-18
5	1083	2685	3080	6848	3768	28.7	71.3	27.32	30.21	1029.38	1138.25	Significant	3.27e-08
6	803	2038	2420	5261	2841	28.3	71.7	26.64	29.95	756.81	850.86	Significant	2.49e-04
7	584	1615	1964	4163	2199	26.6	73.4	24.75	28.44	544.32	625.47		1.00e+00
8	403	1373	1539	3315	1776	22.7	77.3	20.80	24.70	369.46	438.64		1.00e+00
9	373	1028	1402	2803	1401	26.6	73.4	24.37	29.00	341.49	406.30		1.00e+00
10	281	848	1115	2244	1129	24.9	75.1	22.45	27.50	253.50	310.42		1.00e+00
11	204	750	983	1937	954	21.4	78.6	18.90	24.10	180.28	229.91		1.00e+00
12	185	639	846	1670	824	22.5	77.5	19.73	25.43	162.58	209.52		1.00e+00
13	161	564	772	1497	725	22.2	77.8	19.33	25.38	140.13	183.99		1.00e+00
14	144	455	685	1284	599	24.0	76.0	20.79	27.62	124.51	165.47		1.00e+00
15	111	436	592	1139	547	20.3	79.7	17.13	23.87	93.69	130.57		1.00e+00
16	93	372	570	1035	465	20.0	80.0	16.61	23.88	77.23	111.06		1.00e+00
17	96	341	467	904	437	22.0	78.0	18.33	26.09	80.11	114.02		1.00e+00
18	84	279	460	823	363	23.1	76.9	19.09	27.76	69.29	100.75		1.00e+00
19	75	247	411	733	322	23.3	76.7	19.00	28.22	61.17	90.86		1.00e+00
20	77	229	349	655	306	25.2	74.8	20.62	30.32	63.10	92.79		1.00e+00
21	47	231	327	605	278	16.9	83.1	12.94	21.78	35.96	60.55		1.00e+00
22	58	187	344	589	245	23.7	76.3	18.77	29.39	45.98	72.01		1.00e+00
23	44	192	316	552	236	18.6	81.4	14.17	24.13	33.43	56.94		1.00e+00
24	41	145	276	462	186	22.0	78.0	16.66	28.56	30.98	53.12		1.00e+00
25	31	158	261	450	189	16.4	83.6	11.76	22.38	22.23	42.30		1.00e+00
26	24	130	236	390	154	15.6	84.4	10.64	22.20	16.39	34.19		1.00e+00
27	30	128	251	409	158	19.0	81.0	13.59	25.85	21.48	40.85		1.00e+00
28	34	110	202	346	144	23.6	76.4	17.38	31.21	25.03	44.94		1.00e+00
29	24	101	184	309	125	19.2	80.8	13.20	27.04	16.50	33.80		1.00e+00
30	35	71	188	294	106	33.0	67.0	24.78	42.45	26.27	44.99		1.00e+00

[6] Significance calculated compared to baseline of average for original submissions from female corresponding authors (23.9%)

Figure and Table B2b: Breakdown of first author gender with number of submissions (numbers greater than 30 have been omitted)

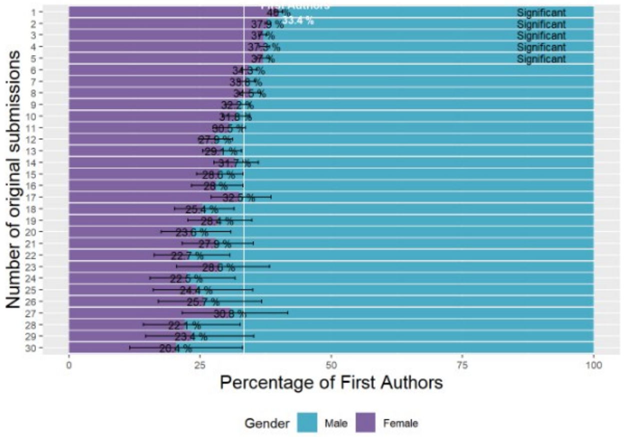

Number of submissions	Female	Male	Unknown	Total For Level	Total Gender Deduced	Female Percentage	Male Percentage	LCI	UCI	LCI Count	UCI Count	Significance[7]	Adjusted P-Value
1	8425	12625	22995	44045	21050	40.0	60.0	39.36	40.69	8286.07	8564.69	Significant	2.12e-85
2	11327	18548	26287	56162	29875	37.9	62.1	37.37	38.47	11163.11	11491.82	Significant	8.33e-56
3	6283	10719	16545	33547	17002	37.0	63.0	36.23	37.68	6160.16	6406.84	Significant	3.30e-18
4	3674	6184	10424	20282	9858	37.3	62.7	36.32	38.23	3580.41	3768.57	Significant	7.15e-12
5	2359	4010	7221	13590	6369	37.0	63.0	35.86	38.23	2283.98	2435.01	Significant	6.74e-06
6	1450	2779	5132	9361	4229	34.3	65.7	32.87	35.73	1390.12	1511.08		1.00e+00
7	983	1927	3825	6735	2910	33.8	66.2	32.08	35.52	933.64	1033.60		1.00e+00
8	699	1329	2864	4892	2028	34.5	65.5	32.43	36.56	657.68	741.51		1.00e+00
9	505	1064	2314	3883	1569	32.2	67.8	29.92	34.54	469.44	541.92		1.00e+00
10	380	814	1705	2899	1194	31.8	68.2	29.24	34.52	349.18	412.21		1.00e+00
11	264	602	1410	2276	866	30.5	69.5	27.51	33.63	238.23	291.26		1.00e+00
12	204	528	1145	1877	732	27.9	72.1	24.74	31.23	181.10	228.59		1.00e+00
13	171	417	989	1577	588	29.1	70.9	25.55	32.88	150.26	193.34		1.00e+00
14	146	314	722	1182	460	31.7	68.3	27.65	36.13	127.19	166.21		1.00e+00
15	115	287	625	1027	402	28.6	71.4	24.40	33.22	98.10	133.53		1.00e+00
16	90	231	559	880	321	28.0	72.0	23.40	33.20	75.11	106.56		1.00e+00
17	83	172	451	706	255	32.5	67.5	27.09	38.53	69.07	98.25		1.00e+00
18	58	170	400	628	228	25.4	74.6	20.21	31.48	46.08	71.78		1.00e+00
19	59	149	341	549	208	28.4	71.6	22.66	34.85	47.14	72.49		1.00e+00
20	37	120	297	454	157	23.6	76.4	17.58	30.82	27.60	48.38		1.00e+00
21	46	119	232	397	165	27.9	72.1	21.58	35.18	35.61	58.05		1.00e+00
22	29	99	256	384	128	22.7	77.3	16.22	30.69	20.76	39.28		1.00e+00
23	28	70	223	321	98	28.6	71.4	20.53	38.23	20.12	37.46		1.00e+00
24	23	79	188	290	102	22.5	77.5	15.46	31.63	15.77	32.26		1.00e+00
25	19	59	164	242	78	24.4	75.6	16.11	35.01	12.57	27.31		1.00e+00
26	19	55	136	210	74	25.7	74.3	17.03	36.72	12.60	27.18		1.00e+00
27	24	54	134	212	78	30.8	69.2	21.59	41.75	16.84	32.57		1.00e+00
28	17	60	120	197	77	22.1	77.9	14.18	32.63	10.91	25.13		1.00e+00
29	15	49	108	172	64	23.4	76.6	14.65	35.24	9.37	22.55		1.00e+00
30	11	43	100	154	54	20.4	79.6	11.60	33.07	6.27	17.86		1.00e+00

[7] Significance calculated compared to baseline of average for original submissions from female first authors (33.4%)

Table B3a (for Figures 4): Breakdown of submissions with corresponding author country and gender (only top 20 countries have been included)

Country	Female	Male	Unknown	Total For Level	Total Gender Deduced	Female Percentage	Male Percentage	LCI	UCI	LCI Count	UCI Count	Significance[8]	Adjusted P-Value
China	14058	36449	240233	290740	50507	27.8	72.2	27.44	28.23	13861.44	14256.26	Significant	1.62e-87
India	7728	23340	45072	76140	31068	24.9	75.1	24.40	25.36	7579.63	7878.30		1.00e+00
USA	7703	29274	19936	56913	36977	20.8	79.2	20.42	21.25	7551.06	7857.18	Significant	3.14e-40
South Korea	900	6304	21220	28424	7204	12.5	87.5	11.75	13.28	846.41	956.47	Significant	4.94e-127
Japan	1501	12885	9758	24144	14386	10.4	89.6	9.94	10.94	1430.63	1574.41	Significant	7.11e-320
United Kingdom	3113	14175	3569	20857	17288	18.0	82.0	17.44	18.59	3015.20	3213.25	Significant	3.54e-74
Germany	2909	13691	4139	20739	16600	17.5	82.5	16.95	18.11	2814.24	3006.26	Significant	3.52e-84
Ireland	1626	12572	5679	19877	14198	11.5	88.5	10.94	11.99	1553.09	1701.87	Significant	9.65e-306
France	3209	8561	2853	14623	11770	27.3	72.7	26.47	28.08	3115.19	3304.56	Significant	3.93e-13
Spain	3317	8431	2831	14579	11748	28.2	71.8	27.43	29.06	3222.22	3413.45	Significant	3.78e-23
Taiwan	367	937	11162	12466	1304	28.1	71.9	25.77	30.65	336.03	399.64		5.20e-01
Italy	4384	6032	978	11394	10416	42.1	57.9	41.14	43.04	4285.57	4483.04	Significant	5.15e-320
Australia	1352	5647	3673	10672	6999	19.3	80.7	18.41	20.26	1288.44	1417.91	Significant	2.73e-16
Canada	1218	5274	2967	9459	6492	18.8	81.2	17.83	19.73	1157.54	1280.86	Significant	1.57e-19
Singapore	476	2185	5412	8073	2661	17.9	82.1	16.48	19.39	438.47	515.99	Significant	1.79e-10
Brazil	1659	3807	1886	7352	5466	30.4	69.6	29.15	31.58	1593.15	1726.36	Significant	7.33e-24
Poland	2481	3301	777	6559	5782	42.9	57.1	41.64	44.19	2407.53	2555.01	Significant	5.62e-217
Russia	1261	3582	578	5421	4843	26.0	74.0	24.82	27.29	1202.07	1321.77		1.00e+00
Turkey	865	2203	1487	4555	3068	28.2	71.8	26.63	29.81	817.01	914.67	Significant	1.41e-04
Switzerland	769	2617	768	4154	3386	22.7	77.3	21.33	24.15	722.27	817.83		1.00e+00

[8] Significance calculated compared to baseline of average for submissions from female corresponding authors (23.9%)

Table B3b (for Figure 5): Breakdown of submissions by corresponding author gender calculated by gender-guesser method with continent of corresponding author address

Country	Female	Male	Unknown	Total For Level	Total Gender Deduced	Female Percentage	Male Percentage	LCI	UCI	LCI Count	UCI Count	Significance[9]	Adjusted P-Value
Asia	15470	59414	394005	468889	74884	20.7	79.3	20.37	20.95	15253.99	15688.27	Significant	2.59e-94
Europe	28446	85022	31662	145130	113468	25.1	74.9	24.82	25.32	28160.81	28733.10	Significant	4.49e-15
Americas	9788	35885	33681	79354	45673	21.4	78.6	21.06	21.81	9617.22	9960.98	Significant	2.54e-31
Oceania	1428	5178	5019	11625	6606	21.6	78.4	20.64	22.63	1363.52	1494.66		7.61e-02
Africa	1489	3593	3166	8248	5082	29.3	70.7	28.06	30.57	1426.22	1553.37	Significant	6.21e-15
Other	2	4	5	11	6	33.3	66.7	9.25	70.43	0.56	4.23		1.00e+00

Table B3c (for Figure 5): Breakdown of submissions by corresponding author gender calculated by ONS method with continent of corresponding author address

Country	Female	Male	Unknown	Total For Level	Total Gender Deduced	Female Percentage	Male Percentage	LCI	UCI	LCI Count	UCI Count	Significance[10]	Adjusted P-Value
Asia	29760	96740	342693	469193	126500	23.5	76.5	23.29	23.76	29465.34	30056.70		1.00e+00
Europe	28719	86601	29811	145131	115320	24.9	75.1	24.65	25.15	28432.13	29007.80	Significant	2.07e-10
Americas	11937	41657	25763	79357	53594	22.3	77.7	21.92	22.63	11749.27	12126.86	Significant	3.01e-14
Oceania	1579	6193	3853	11625	7772	20.3	79.7	19.44	21.23	1510.62	1649.66	Significant	3.86e-10
Africa	1881	3748	2618	8247	5629	33.4	66.6	32.20	34.66	1812.29	1950.98	Significant	1.21e-54
Other	2	5	6	13	7	28.6	71.4	7.56	64.76	0.53	4.53		1.00e+00

[9] Significance calculated compared to baseline of average for submissions from female corresponding authors calculated by gender-guesser(23.1%)

[10] Significance calculated compared to baseline of average for submissions from female corresponding authors calculated by ONS method (23.9%)

Table B4a (for Figure 6): Breakdown of submissions by corresponding author gender and number of authors

Number of authors	Female	Male	Unknown	Total For Level	Total Gender Deduced	Female Percentage	Male Percentage	LCI	UCI	LCI Count	UCI Count	Significance[11]	Adjusted P-Value
1	1554	6365	7313	15232	7919	19.6	80.4	18.76	20.51	1485.89	1624.44	Significant	6.33e-16
2	7388	25482	30251	63121	32870	22.5	77.5	22.03	22.93	7240.73	7537.39	Significant	3.81e-05
3	10768	36912	48264	95944	47680	22.6	77.4	22.21	22.96	10590.10	10948.00	Significant	5.87e-07
4	12457	40170	62075	114702	52627	23.7	76.3	23.31	24.04	12266.90	12649.13		1.00e+00
5	11707	36686	69013	117406	48393	24.2	75.8	23.81	24.58	11523.35	11892.63		1.00e+00
6	9861	30469	64817	105147	40330	24.5	75.5	24.03	24.87	9692.81	10031.15		1.00e+00
7	7571	21849	48058	77478	29420	25.7	74.3	25.24	26.24	7424.97	7718.90	Significant	7.57e-09
8	5101	14970	32349	52420	20071	25.4	74.6	24.82	26.02	4981.06	5222.83	Significant	1.16e-02
9	3145	9951	22079	35175	13096	24.0	76.0	23.29	24.75	3050.19	3241.81		1.00e+00
10	2314	6349	13541	22204	8663	26.7	73.3	25.79	27.65	2234.19	2395.60	Significant	1.17e-05
11	950	2790	3688	7428	3740	25.4	74.6	24.03	26.82	898.78	1003.11		1.00e+00
12	638	1779	2264	4681	2417	26.4	73.6	24.68	28.19	596.45	681.36		1.00e+00
13	335	990	1295	2620	1325	25.3	74.7	23.02	27.69	304.95	366.94		1.00e+00
14	191	601	605	1397	792	24.1	75.9	21.26	27.22	168.41	215.57		1.00e+00
15	117	364	377	858	481	24.3	75.7	20.70	28.36	99.56	136.40		1.00e+00
16	74	198	146	418	272	27.2	72.8	22.25	32.79	60.53	89.20		1.00e+00
17	48	120	133	301	168	28.6	71.4	22.26	35.84	37.40	60.21		1.00e+00
18	21	88	81	190	109	19.3	80.7	12.89	27.74	14.05	30.23		1.00e+00
19	11	46	46	103	57	19.3	80.7	10.96	31.51	6.25	17.96		1.00e+00
20	13	25	32	70	38	34.2	65.8	21.15	50.17	8.04	19.07		1.00e+00
21	16	14	22	52	30	53.3	46.7	36.14	69.77	10.84	20.93	Significant	2.27e-02
22	10	28	21	59	38	26.3	73.7	14.81	42.17	5.63	16.02		1.00e+00
23	4	6	3	13	10	40.0	60.0	16.71	68.84	1.67	6.88		1.00e+00
24	0	8	6	14	8	0.0	100.0	-4.78	37.22	NaN	NaN		1.00e+00
25	0	11	1	12	11	0.0	100.0	-4.14	30.02	NaN	NaN		8.51e-01
26	0	6	1	7	6	0.0	100.0	-5.24	44.28	NaN	NaN		1.00e+00
27	1	12	3	16	13	7.7	92.3	-0.74	35.42	-0.10	4.61		1.00e+00
28	0	2	2	4	2	0.0	100.0	-5.22	70.98	NaN	NaN		1.00e+00
29	1	2	2	5	3	33.3	66.7	5.63	79.76	0.17	2.39		1.00e+00
30	0	2	1	3	2	0.0	100.0	-5.22	70.98	NaN	NaN		1.00e+00

[11] Significance calculated compared to baseline of average for original submissions from female corresponding authors (23.9%)

Table B4b: Breakdown of submissions by first author gender and corresponding author gender

First author gender	Female	Male	Unknown	Total For Level	Total Gender Deduced	Female Percentage	Male Percentage	LCI	UCI	LCI Count	UCI Count	Significance[12]	Adjusted P-Value
F	12768	33298	30127	76193	46066	27.7	72.3	27.31	28.13	12580.57	12957.14	Significant	7.30e-75
M	13862	58424	55522	127808	72286	19.2	80.8	18.89	19.47	13655.73	14070.64	Significant	9.61e-200
U	17598	60957	200235	278790	78555	22.4	77.6	22.11	22.70	17370.02	17828.10	Significant	2.93e-18

[12] Significance calculated compared to baseline of average for original submissions from female corresponding authors (23.9%)

Table B5a (for Figure 9): Breakdown of submissions by corresponding author gender and date

Date	Female	Male	Unknown	Total For Level	Total Gender Deduced	Female Percentage	Male Percentage	LCI	UCI	LCI Count	UCI Count	Significance[13]	Adjusted P-Value
2014 Q1	2629	8169	13717	24515	10798	24.3	75.7	23.55	25.17	2542.58	2717.39		1
2014 Q2	2283	7355	12248	21886	9638	23.7	76.3	22.85	24.55	2202.21	2365.82		1
2014 Q3	2210	6849	11707	20766	9059	24.4	75.6	23.52	25.29	2130.87	2291.09		1
2014 Q4	2571	8098	14425	25094	10669	24.1	75.9	23.30	24.92	2485.42	2658.57		1
2015 Q1	3065	9820	17268	30153	12885	23.8	76.2	23.06	24.53	2971.28	3160.73		1
2015 Q2	4023	13617	22747	40387	17640	22.8	77.2	22.19	23.43	3914.82	4133.27		1
2015 Q3	4480	14471	24847	43798	18951	23.6	76.4	23.04	24.25	4366.38	4595.65		1
2015 Q4	4419	14758	25859	45036	19177	23.0	77.0	22.45	23.64	4305.74	4534.33		1
2016 Q1	4774	15061	25781	45616	19835	24.1	75.9	23.48	24.67	4656.99	4893.00		1
2016 Q2	5194	16100	27735	49029	21294	24.4	75.6	23.82	24.97	5072.16	5317.80		1
2016 Q3	5297	16397	28316	50010	21694	24.4	75.6	23.85	24.99	5173.97	5421.99		1
2016 Q4	4593	14371	25057	44021	18964	24.2	75.8	23.62	24.83	4478.36	4709.62		1
2017 Q1	3639	11443	19997	35079	15082	24.1	75.9	23.45	24.82	3537.01	3742.98		1
2017 Q2	4660	14382	24009	43051	19042	24.5	75.5	23.87	25.09	4544.71	4777.25		1
2017 Q3	4484	13826	24170	42480	18310	24.5	75.5	23.87	25.12	4370.94	4599.02		1
2017 Q4	4319	13332	23928	41579	17651	24.5	75.5	23.84	25.11	4208.04	4431.92		1
2018 Q1	4055	12692	22122	38869	16747	24.2	75.8	23.57	24.87	3947.34	4164.64		1
2018 Q2	3403	10912	19020	33335	14315	23.8	76.2	23.08	24.48	3304.19	3503.83		1

[13] Significance calculated compared to baseline of average for original submissions from female corresponding authors (23.9%)

Figure and Table B5b: Breakdown of submissions by first author gender and date

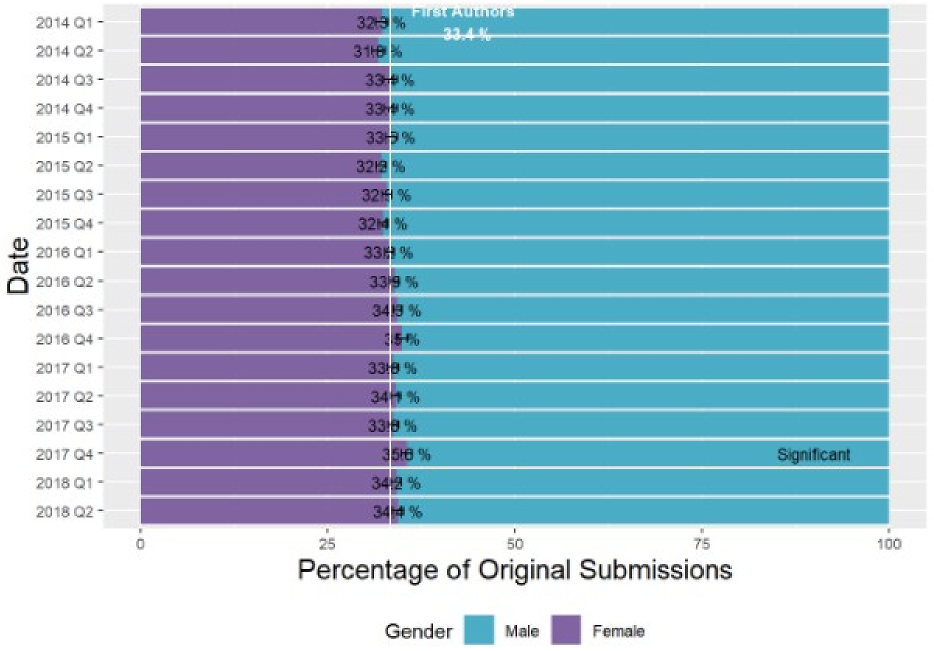

Date	Female		Male	Unknown	Total For Level	Total Gender Deduced	Female Percentage	Male Percentage	LCI	UCI	LCI Count	UCI Count	Significance[14]	Adjusted P-Value
2014 Q1	3549		7454	13512	24515	11003	32.3	67.7	31.39	33.13	3453.59	3645.77		1.0000000
2014 Q2	3120		6680	12086	21886	9800	31.8	68.2	30.92	32.77	3030.32	3211.07		1.0000000
2014 Q3	3127		6244	11395	20766	9371	33.4	66.6	32.42	34.33	3038.19	3217.09		1.0000000
2014 Q4	3696		7363	14035	25094	11059	33.4	66.6	32.55	34.31	3599.42	3793.85		1.0000000
2015 Q1	4452		8828	16873	30153	13280	33.5	66.5	32.73	34.33	4346.02	4559.25		1.0000000
2015 Q2	5845		12324	22218	40387	18169	32.2	67.8	31.49	32.85	5722.28	5969.09		1.0000000
2015 Q3	6307		12874	24617	43798	19181	32.9	67.1	32.22	33.55	6180.15	6435.17		1.0000000
2015 Q4	6448		13442	25146	45036	19890	32.4	67.6	31.77	33.07	6319.30	6578.05		1.0000000
2016 Q1	6825		13710	25081	45616	20535	33.2	66.8	32.59	33.88	6693.35	6957.94		1.0000000
2016 Q2	7417		14464	27148	49029	21881	33.9	66.1	33.27	34.53	7280.39	7554.85		1.0000000
2016 Q3	7586		14503	27921	50010	22089	34.3	65.7	33.72	34.97	7448.29	7724.92		1.0000000
2016 Q4	6816		12683	24522	44021	19499	35.0	65.0	34.29	35.63	6686.09	6947.07		0.0881163
2017 Q1	5234		10262	19583	35079	15496	33.8	66.2	33.04	34.53	5119.24	5350.00		1.0000000
2017 Q2	6585		12730	23736	43051	19315	34.1	65.9	33.43	34.76	6456.50	6714.72		1.0000000
2017 Q3	6301		12334	23845	42480	18635	33.8	66.2	33.14	34.50	6175.06	6428.18		1.0000000
2017 Q4	6386		11562	23631	41579	17948	35.6	64.4	34.88	36.28	6260.85	6512.25	Significant	0.0000133
2018 Q1	5795		11158	21916	38869	16953	34.2	65.8	33.47	34.90	5674.57	5916.64		1.0000000
2018 Q2	4995		9532	18808	33335	14527	34.4	65.6	33.62	35.16	4883.40	5107.80		1.0000000

[14] Significance calculated compared to baseline of average for original submissions from female first authors (33.4%)

Table B6a (for Figure 9): Breakdown of submissions by corresponding author gender and chemistry sub-discipline

Chemistry sub-discipline	Female	Male	Unknown	Total For Level	Total Gender Deduced	Female Percentage	Male Percentage	LCI	UCI	LCI Count	UCI Count	Significance[15]	Adjusted P-Value
Analytical	24652	65752	99884	190288	90404	27.3	72.7	26.98	27.56	24390.43	24915.31	Significant	4.37e-116
Biological	15249	39531	51321	106101	54780	27.8	72.2	27.46	28.21	15044.25	15455.45	Significant	2.65e-95
Catalysis	11161	41544	80591	133296	52705	21.2	78.8	20.83	21.53	10978.27	11345.94	Significant	3.29e-45
Chemical Biology and Medicinal	16669	43408	59232	119309	60077	27.7	72.3	27.39	28.11	16454.76	16884.95	Significant	6.56e-100
Energy	14020	53098	127353	194471	67118	20.9	79.1	20.58	21.20	13814.70	14227.53	Significant	3.86e-72
Environmental	8892	20399	35067	64358	29291	30.4	69.6	29.83	30.89	8738.53	9046.98	Significant	8.76e-136
Food	8347	15346	26582	50275	23693	35.2	64.8	34.62	35.84	8203.46	8491.67	Significant	1.17e-319
Inorganic	8942	33967	37642	80551	42909	20.8	79.2	20.46	21.23	8778.22	9108.02	Significant	7.87e-47
Materials	34889	115070	217713	367672	149959	23.3	76.7	23.05	23.48	34569.34	35210.72	Significant	1.16e-03
Miscellaneous	287	934	740	1961	1221	23.5	76.5	21.21	25.97	258.98	317.04		1.00e+00
Nanoscience	26539	82777	190604	299920	109316	24.3	75.7	24.02	24.53	26262.14	26817.83		1.00e+00
Organic	7992	35713	40993	84698	43705	18.3	81.7	17.93	18.65	7834.83	8151.61	Significant	3.38e-172
Physical	20861	75290	104267	200418	96151	21.7	78.3	21.44	21.96	20611.59	21112.59	Significant	4.98e-54

Table B6b (for Figure 9): Breakdown of submissions by first author gender and journal chemistry sub-discipline

Chemistry sub-discipline	Female	Male	Unknown	Total For Level	Total Gender Deduced	Female Percentage	Male Percentage	LCI	UCI	LCI Count	UCI Count	Significance[16]	Adjusted P-Value
Analytical	34230	57900	98158	190288	92130	37.2	62.8	36.84	37.47	33943	34518	Significant	8.76e-122
Biological	22856	31759	51486	106101	54615	41.8	58.2	41.44	42.26	22630	23082	Significant	2.70e-319
Catalysis	16917	37366	79013	133296	54283	31.2	68.8	30.78	31.56	16706	17129	Significant	6.46e-24
Chemical Biology and Medicinal	24907	35481	58921	119309	60388	41.2	58.8	40.85	41.64	24670	25144	Significant	5.97e-319
Energy	20009	49724	124738	194471	69733	28.7	71.3	28.36	29.03	19776	20244	Significant	4.36e-152
Environmental	11937	18289	34132	64358	30226	39.5	60.5	38.94	40.04	11771	12104	Significant	2.81e-104
Food	11374	13490	25411	50275	24864	45.7	54.3	45.13	46.36	11220	11528	Significant	1.23e-319
Inorganic	13570	29293	37688	80551	42863	31.7	68.3	31.22	32.10	13382	13759	Significant	7.28e-10
Materials	50411	102828	214433	367672	153239	32.9	67.1	32.66	33.13	50051	50772		1.00e+00
Miscellaneous	417	781	763	1961	1198	34.8	65.2	32.16	37.55	385	450		1.00e+00
Nanoscience	38382	76077	185461	299920	114459	33.5	66.5	33.26	33.81	38069	38696		1.00e+00
Organic	12717	31205	40776	84698	43922	29.0	71.0	28.53	29.38	12532	12904	Significant	2.82e-84
Physical	28905	67542	103971	200418	96447	30.0	70.0	29.68	30.26	28627	29185	Significant	5.54e-110

[15] Significance calculated compared to baseline of average for original submissions from female corresponding authors (23.9%)
[16] Significance calculated compared to baseline of average for original submissions from female first authors (33.4%)

Table B6c (for Figure 10): Binomial GLM model of corresponding author gender of original submissions, chemistry sub-discipline and journal impact factor

Impact factor (IF 2017)	Chemistry sub-discipline (cats)	fit[17]	Standard Error	LCI	UCI	Population Size
1.6	Analytical	0.327	0.013	0.323	0.330	26929
1.6	Biological	0.337	0.018	0.332	0.342	11190
1.6	Catalysis	0.231	0.021	0.226	0.235	10821
1.6	Chemical Biology and Medicinal	0.326	0.017	0.322	0.331	14891
1.6	Energy	0.232	0.020	0.227	0.236	11746
1.6	Environmental	0.330	0.019	0.325	0.336	13840
1.6	Food	0.409	0.021	0.402	0.415	8806
1.6	Inorganic	0.261	0.025	0.255	0.267	4082
1.6	Materials	0.267	0.012	0.264	0.270	29024
1.6	Miscellaneous	0.391	0.095	0.362	0.420	436
1.6	Nanoscience	0.280	0.014	0.277	0.284	21212
1.6	Organic	0.223	0.023	0.218	0.228	8540
1.6	Physical	0.241	0.015	0.237	0.244	15317
10	Analytical	0.185	0.024	0.180	0.190	5022
10	Biological	0.197	0.028	0.192	0.203	4391
10	Catalysis	0.196	0.023	0.191	0.201	7065
10	Chemical Biology and Medicinal	0.205	0.027	0.199	0.211	4420
10	Energy	0.203	0.017	0.200	0.206	17427
10	Environmental	0.261	0.037	0.252	0.270	1704
10	Food	0.229	0.048	0.218	0.240	730
10	Inorganic	0.143	0.037	0.137	0.149	4311
10	Materials	0.203	0.014	0.200	0.206	20561
10	Miscellaneous	0.039	0.257	0.028	0.053	111
10	Nanoscience	0.214	0.015	0.211	0.217	14377
10	Organic	0.135	0.032	0.131	0.140	4397
10	Physical	0.192	0.021	0.188	0.196	9548
13	Analytical	0.147	0.035	0.142	0.153	173
13	Biological	0.159	0.042	0.152	0.167	102
13	Catalysis	0.184	0.036	0.178	0.191	65
13	Chemical Biology and Medicinal	0.171	0.041	0.163	0.178	83
13	Energy	0.193	0.026	0.188	0.199	451
13	Environmental	0.238	0.054	0.226	0.251	51
13	Food	0.180	0.070	0.167	0.194	28
13	Inorganic	0.113	0.056	0.106	0.121	61
13	Materials	0.183	0.021	0.179	0.187	1042
13	Miscellaneous	0.015	0.365	0.009	0.023	0
13	Nanoscience	0.193	0.024	0.189	0.198	793
13	Organic	0.112	0.049	0.106	0.118	6
13	Physical	0.176	0.031	0.170	0.182	413
4.5	Analytical	0.272	0.008	0.270	0.274	34209
4.5	Biological	0.283	0.010	0.281	0.286	22141
4.5	Catalysis	0.218	0.012	0.216	0.221	18830
4.5	Chemical Biology and Medicinal	0.280	0.009	0.278	0.283	25333
4.5	Energy	0.222	0.012	0.219	0.224	13224
4.5	Environmental	0.305	0.013	0.302	0.309	7329
4.5	Food	0.341	0.015	0.336	0.345	10044
4.5	Inorganic	0.214	0.012	0.212	0.217	23890
4.5	Materials	0.243	0.007	0.242	0.245	50249
4.5	Miscellaneous	0.198	0.083	0.181	0.215	354
4.5	Nanoscience	0.256	0.008	0.254	0.258	28101
4.5	Organic	0.189	0.013	0.186	0.191	17181
4.5	Physical	0.223	0.008	0.221	0.225	44398
7.4	Analytical	0.223	0.014	0.220	0.226	21561
7.4	Biological	0.235	0.017	0.231	0.239	13109
7.4	Catalysis	0.206	0.014	0.203	0.209	13612
7.4	Chemical Biology and Medicinal	0.239	0.016	0.235	0.242	12592
7.4	Energy	0.212	0.011	0.209	0.214	14507
7.4	Environmental	0.281	0.024	0.275	0.287	4836
7.4	Food	0.278	0.030	0.271	0.286	2900
7.4	Inorganic	0.174	0.021	0.170	0.178	9603
7.4	Materials	0.221	0.008	0.219	0.223	41470
7.4	Miscellaneous	0.086	0.167	0.071	0.105	291
7.4	Nanoscience	0.233	0.009	0.231	0.235	39499
7.4	Organic	0.159	0.019	0.156	0.162	12878
7.4	Physical	0.206	0.012	0.203	0.209	22417

[17] Model fit: CorrespondingAuthorGender ~cats * IF2017

Figure Table B6d: Binomial GLM model of first author gender of original submissions, chemistry sub-discipline and journal impact factor

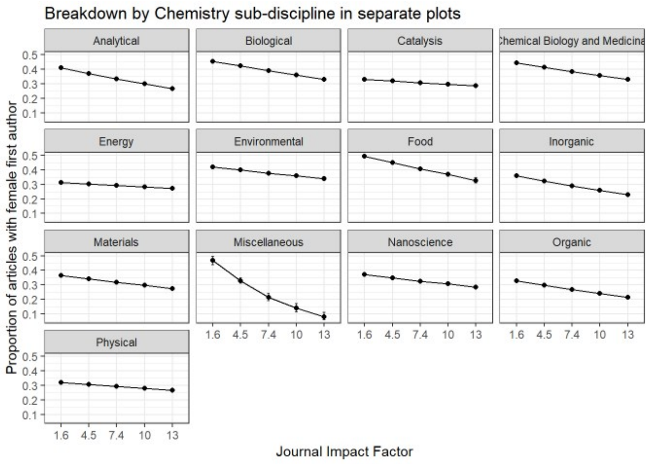

Impact factor (IF 2017)	Chemistry sub-discipline (cats)	fit[18]	Standard Error	LCI	UCI	Population Size
1.6	Analytical	0.409	0.012	0.406	0.413	28428
1.6	Biological	0.456	0.017	0.451	0.461	11332
1.6	Catalysis	0.330	0.018	0.325	0.336	11578
1.6	Chemical Biology and Medicinal	0.446	0.015	0.441	0.451	15188
1.6	Energy	0.313	0.017	0.308	0.318	12657
1.6	Environmental	0.419	0.018	0.413	0.425	14741
1.6	Food	0.493	0.020	0.486	0.499	9286
1.6	Inorganic	0.359	0.021	0.353	0.365	4358
1.6	Materials	0.363	0.010	0.360	0.367	31543
1.6	Miscellaneous	0.468	0.089	0.440	0.496	428
1.6	Nanoscience	0.372	0.013	0.368	0.376	23373
1.6	Organic	0.327	0.019	0.322	0.333	8762
1.6	Physical	0.320	0.013	0.317	0.324	16085
10	Analytical	0.301	0.021	0.295	0.306	4761
10	Biological	0.362	0.024	0.355	0.369	4035
10	Catalysis	0.298	0.020	0.292	0.303	7209
10	Chemical Biology and Medicinal	0.358	0.024	0.351	0.365	4173
10	Energy	0.283	0.015	0.279	0.286	18363
10	Environmental	0.360	0.034	0.350	0.370	1824
10	Food	0.368	0.043	0.355	0.381	695
10	Inorganic	0.258	0.031	0.251	0.266	4169
10	Materials	0.297	0.012	0.294	0.301	20812
10	Miscellaneous	0.139	0.188	0.112	0.170	106
10	Nanoscience	0.306	0.013	0.303	0.310	15225
10	Organic	0.242	0.026	0.236	0.248	4256
10	Physical	0.279	0.018	0.274	0.284	9293
13	Analytical	0.266	0.031	0.258	0.274	174
13	Biological	0.330	0.037	0.320	0.341	104
13	Catalysis	0.286	0.031	0.278	0.294	86
13	Chemical Biology and Medicinal	0.329	0.036	0.319	0.339	98
13	Energy	0.272	0.023	0.267	0.278	460
13	Environmental	0.339	0.049	0.325	0.354	48
13	Food	0.327	0.063	0.309	0.345	27
13	Inorganic	0.227	0.047	0.217	0.238	54
13	Materials	0.276	0.019	0.271	0.280	1020
13	Miscellaneous	0.081	0.270	0.059	0.111	0
13	Nanoscience	0.284	0.021	0.279	0.290	784
13	Organic	0.215	0.040	0.207	0.224	6
13	Physical	0.265	0.028	0.258	0.272	428
4.5	Analytical	0.370	0.007	0.368	0.372	34885
4.5	Biological	0.423	0.009	0.420	0.426	22089
4.5	Catalysis	0.319	0.010	0.316	0.322	19050
4.5	Chemical Biology and Medicinal	0.415	0.008	0.413	0.418	25511
4.5	Energy	0.302	0.011	0.299	0.305	13468

[18] Model: FirstAuthorGender ~ Category * ImpactFactor. ANOVA: Category is highly significant (p = 0.00e00); ImpactFactor is highly significant (p = 4.61e-263); Category*ImpactFactor interactions are highly significant (p = 5.68e-34)

4.5	Environmental	0.398	0.012	0.394	0.402	7381
4.5	Food	0.449	0.014	0.444	0.453	10704
4.5	Inorganic	0.322	0.011	0.319	0.325	23972
4.5	Materials	0.340	0.006	0.338	0.342	50428
4.5	Miscellaneous	0.329	0.066	0.310	0.348	371
4.5	Nanoscience	0.349	0.007	0.347	0.351	29155
4.5	Organic	0.296	0.011	0.293	0.299	17082
4.5	Physical	0.306	0.007	0.304	0.308	44097
7.4	Analytical	0.333	0.013	0.329	0.336	21443
7.4	Biological	0.390	0.014	0.386	0.395	13213
7.4	Catalysis	0.308	0.012	0.304	0.311	13936
7.4	Chemical Biology and Medicinal	0.385	0.014	0.381	0.389	12648
7.4	Energy	0.292	0.010	0.289	0.294	15105
7.4	Environmental	0.378	0.022	0.371	0.384	4708
7.4	Food	0.406	0.027	0.397	0.414	2966
7.4	Inorganic	0.287	0.018	0.283	0.292	9391
7.4	Materials	0.317	0.007	0.315	0.319	42098
7.4	Miscellaneous	0.214	0.120	0.189	0.241	265
7.4	Nanoscience	0.326	0.008	0.324	0.328	40684
7.4	Organic	0.267	0.016	0.263	0.271	13126
7.4	Physical	0.291	0.011	0.288	0.294	22583

Table C1 (for Figure 11): Breakdown of submissions by editor gender and journal editorial model

Journal editorial model	Female	Male	Unknown	Total For Level	Total Gender Deduced	Female Percentage	Male Percentage	LCI	UCI	Significance[19]	Adjusted P-Value
AE	59236	106205	60171	225612	165441	35.8	64.2	35.57	36.04	Significant	1.32e-316
AE (with in-house pre-screening)	13107	22092	30778	65977	35199	37.2	62.8	36.73	37.74	Significant	2.06e-29
AE (with in-house pre-submission assessment)	526	724	291	1541	1250	42.1	57.9	39.37	44.84		1.00e+00
Hybrid	109186	98552	38723	246461	207738	52.6	47.4	52.34	52.77	Significant	3.08e-318
In-house	12740	10169	189	23098	22909	55.6	44.4	54.97	56.25	Significant	1.13e-319
Other	9	9	1	19	18	50.0	50.0	29.03	70.97		1.00e+00

[19] Significance calculated compared to baseline of average for unique editors (40.4%)

Table C2a: Breakdown of submissions by corresponding author gender and whether the submission was rejected before peer review

Rejected without peer review?	Female	Male	Unknown	Total For Level	Total Gender Deduced	Female Percentage	Male Percentage	LCI	UCI	LCI Count	UCI Count	Significance[20]	Adjusted P-Value
Peer reviewed	51502	170106	263635	485243	221608	23.2	76.8	23.06	23.42	51113.33	51892.72	Significant	6.15e-08
Rejected without peer review	22804	66204	142857	231865	89008	25.6	74.4	25.33	25.91	22549.68	23060.19	Significant	6.79e-28

Table C2b: Breakdown of submissions by first author gender and whether the submission was rejected before peer review

Rejected without peer review?	Female	Male	Unknown	Total For Level	Total Gender Deduced	Female Percentage	Male Percentage	LCI	UCI	LCI Count	UCI Count	Significance[21]	Adjusted P-Value
Peer reviewed	74160	149565	261518	485243	223725	33.1	66.9	32.95	33.34	73724.24	74597.05		1.0000
Rejected without peer review	32111	61874	137880	231865	93985	34.2	65.8	33.86	34.47	31826.64	32396.57		0.0631

[20] Significance calculated compared to baseline of average for original submissions from female corresponding authors (23.9%)

[21] Significance calculated compared to baseline of average for original submissions from female first authors (33.4%)

Table C2c (for Figure 12): Binomial GLM model of proportion of submissions rejected without peer review and corresponding author gender controlled by whether the publication is single-authored or not

SingleAuthor	CorrespondingAuthorGender	fit[22]	Standard Error	LCI	UCI	Population Size
FALSE	Female (F)	0.315	0.0080	0.313	0.317	72752
FALSE	Male (M)	0.289	0.0046	0.287	0.290	229945
TRUE	Female (F)	0.430	0.0512	0.413	0.446	1554
TRUE	Male (M)	0.398	0.0256	0.390	0.406	6365

[22] Model fit: RejectedWithoutPeerReview ~ SingleAuthor * CorrespondingAuthorGender

Table C3a (for Figure 13a): Binomial GLM model of proportion of original submissions rejected without peer review, corresponding author gender and editor gender

EditorGender	CorrespondingAuthorGender	fit[23]	Standard Error	LCI	UCI	Population Size
Female (F)	Female (F)	0.337	0.015	0.332	0.341	20331
Male (M)	Female (F)	0.295	0.014	0.291	0.299	25453
Female (F)	Male (M)	0.312	0.008	0.310	0.315	65252
Male (M)	Male (M)	0.274	0.008	0.272	0.276	82357

[23] Model fit: RejectedWithoutPeerReview ~ CorrespondingAuthorGender * EditorGender

Table C3b (for Figure 13b): Binomial GLM model of proportion of original submissions rejected without peer review, corresponding author gender and journal editorial model

EditorialModelOfJournal	CorrespondingAuthorGender	fit[24]	Standard Error	LCI	UCI	Population Size
Associate Editors (AE)	Female (F)	0.268	0.013	0.265	0.271	31104
AE (with in-house pre-submission assessment)	Female (F)	0.128	0.199	0.102	0.159	227
Hybrid	Female (F)	0.314	0.012	0.311	0.317	31865
In-house	Female (F)	0.469	0.019	0.463	0.475	11108
Associate Editors (AE)	Male (M)	0.249	0.008	0.247	0.251	84128
AE (with in-house pre-submission assessment)	Male (M)	0.134	0.085	0.122	0.147	1188
Hybrid	Male (M)	0.276	0.007	0.274	0.277	111024
In-house	Male (M)	0.430	0.010	0.427	0.433	39947

[24] Model fit: RejectedWithoutPeerReview ~ CorrespondingAuthorGender * EditorialModelOfJournal

Table D1: Breakdown of reviewer invitations by reviewer gender and response

Reviewer response	Female	Male	Unknown	Total For Level	Total Gender Deduced	Female Percentage	Male Percentage	LCI	UCI	LCI Count	UCI Count	Significance[25]	Adjusted P-Value
Agreed	52663	192820	246056	491539	245483	21.5	78.5	21.29	21.62	52265	53063	Significant	5.00e-10
Declined	45149	162488	144593	352230	207637	21.7	78.3	21.57	21.92	44782	45518	Significant	1.15e-20
No Response	32640	133847	151768	318255	166487	19.6	80.4	19.42	19.80	32324	32959	Significant	1.85e-28

[25] Significance calculated compared to average for reviewer invitations to female reviewers (20.8%)

Table D2a: Breakdown of proportion of reviews by reviewer gender and corresponding author gender

Corresponding Author Gender	Female	Male	Unknown	Total For Level	Total Gender Deduced	Female Percentage	Male Percentage	LCI	UCI	LCI Count	UCI Count	Significance[26]	Adjusted P-Value
U	47533	176006	270986	494525	223539	21.3	78.7	21.09	21.43	47155	47913	Significant	1.59e-02
M	34620	144873	135091	314584	179493	19.3	80.7	19.11	19.47	34294	34949	Significant	7.26e-52
F	12810	40868	41263	94941	53678	23.9	76.1	23.51	24.23	12617	13005	Significant	1.54e-61

[26] Significance calculated compared to average for reviewer invitations to female reviewers (17.5%)

Table D2b (for Figure 14a): Binomial GLM model of proportion of reviews by female reviewers, corresponding author gender and reviewer response

Response	CorrespondingAuthorGender	fit[27]	Standard Error	LCI	UCI	Population Size
Agreed	F	0.259	0.014	0.255	0.263	23840
Declined	F	0.249	0.0150	0.246	0.253	23730
No Response	F	0.220	0.021	0.215	0.224	13844
Agreed	M	0.194	0.009	0.192	0.196	78844
Declined	M	0.204	0.009	0.202	0.206	70737
No Response	M	0.183	0.012	0.181	0.185	48336

[27] Model fit: ReviewerGender ~ CorrespondingAuthorGender * Response

Table D2c (for Figure 14b): Binomial GLM model of proportion of invitations by female reviewers, corresponding author gender and editor gender

EditorGender	CorrespondingAuthorGender	fit[28]	Standard Error	LCI	UCI	Population Size
Female (F)	Female (F)	0.256769	0.0207664	0.2517231	0.2618806	12151
Male (M)	Female (F)	0.2313658	0.0179076	0.2273098	0.2354722	17535
Female (F)	Male (M)	0.2165862	0.0120788	0.2139712	0.2192243	40395
Male (M)	Male (M)	0.1814619	0.0106878	0.1794363	0.1835052	58938

[28] Model fit: ReviewerGender ~ CorrespondingAuthorGender * EditorGender

Table D3a (for Figure 15a): Breakdown of reviews by reviewer gender and date

Date	Female	Male	Unknown	Total For Level	Total Gender Deduced	Female Percentage	Male Percentage	LCI	UCI	LCI Count	UCI Count	Significance[29]	Adjusted P-Value
2014 Q1	4539	18682	19673	42894	23221	19.5	80.5	19.04	20.06	4421.73	4658.61		0.0521991
2014 Q2	4093	16820	18692	39605	20913	19.6	80.4	19.04	20.11	3981.71	4206.62		0.2218114
2014 Q3	3791	15480	17455	36726	19271	19.7	80.3	19.12	20.24	3684.01	3900.32		1.0000000
2014 Q4	5008	18744	23632	47384	23752	21.1	78.9	20.57	21.61	4885.90	5132.32		1.0000000
2015 Q1	4728	17875	23166	45769	22603	20.9	79.1	20.39	21.45	4609.27	4848.96		1.0000000
2015 Q2	5502	20426	27151	53079	25928	21.2	78.8	20.73	21.72	5374.07	5632.14		1.0000000
2015 Q3	5683	20582	27811	54076	26265	21.6	78.4	21.14	22.14	5553.29	5814.88		1.0000000
2015 Q4	5886	21798	28587	56271	27684	21.3	78.7	20.78	21.75	5753.67	6020.53		1.0000000
2016 Q1	5591	20531	25640	51762	26122	21.4	78.6	20.91	21.90	5462.17	5722.02		1.0000000
2016 Q2	5687	21451	25601	52739	27138	21.0	79.0	20.48	21.44	5556.71	5819.52		1.0000000
2016 Q3	5527	20390	26373	52290	25917	21.3	78.7	20.83	21.83	5398.86	5657.34		1.0000000
2016 Q4	4938	18139	23511	46588	23077	21.4	78.6	20.87	21.93	4816.99	5061.21		1.0000000
2017 Q1	3721	14757	18125	36603	18478	20.1	79.9	19.57	20.72	3615.30	3828.99		1.0000000
2017 Q2	4698	18403	23500	46601	23101	20.3	79.7	19.82	20.86	4579.23	4819.04		1.0000000
2017 Q3	4526	17376	22750	44652	21902	20.7	79.3	20.13	21.21	4409.68	4644.57		1.0000000
2017 Q4	4643	16948	22190	43781	21591	21.5	78.5	20.96	22.06	4525.77	4762.42		1.0000000
2018 Q1	4227	15886	20517	40630	20113	21.0	79.0	20.46	21.58	4114.86	4341.36		1.0000000
2018 Q2	3681	13671	17496	34848	17352	21.2	78.8	20.61	21.83	3576.56	3787.66		1.0000000

Table D3b (for Figure 15b): Breakdown of reviews by reviewer gender and number of reviewers for that submission

Number of reviewers	Female	Male	Unknown	Total For Level	Total Gender Deduced	Female Percentage	Male Percentage	LCI	UCI	LCI Count	UCI Count	Significance[30]	Adjusted P-Value
1	14365	52559	61814	128738	66924	21.5	78.5	21.16	21.78	14157.92	14574.27		1
2	50422	191643	238853	480918	242065	20.8	79.2	20.67	20.99	50031.52	50814.72		1
3	26123	101566	127431	255120	127689	20.5	79.5	20.24	20.68	25841.61	26406.66		1
4	3688	14401	16979	35068	18089	20.4	79.6	19.81	20.98	3582.93	3795.34		1
5	323	1414	2028	3765	1737	18.6	81.4	16.83	20.49	292.41	356.00		1
6	40	144	200	384	184	21.7	78.3	16.36	28.27	30.11	52.02		1
7	2	19	28	49	21	9.5	90.5	1.45	30.12	0.30	6.33		1
8	0	1	7	8	1	0.0	100.0	-3.90	83.25	-	-		1

Table D3c (for Figure 15c): Breakdown of reviews by reviewer gender and number of revisions

[29] Significance calculated compared to baseline of average for reviews from female reviewers (20.8%)
[30] Significance calculated compared to baseline of average for reviews from female reviewers (20.8%)

Revision	Female	Male	Unknown	Total For Level	Total Gender Deduced	Female Percentage	Male Percentage	LCI	UCI	LCI Count	UCI Count	Significance[31]	Adjusted P-Value
0	76502	298193	367428	742123	374695	20.4	79.6	20.29	20.55	76019.53	76986.75	Significant	2.70e-03
1	16023	56573	72677	145273	72596	22.1	77.9	21.77	22.37	15805.06	16243.08	Significant	3.94e-12
2	2070	6173	6511	14754	8243	25.1	74.9	24.19	26.06	1993.79	2148.12	Significant	3.46e-17
3	301	704	633	1638	1005	30.0	70.0	27.20	32.86	273.34	330.19	Significant	8.25e-09
4	51	90	80	221	141	36.2	63.8	28.69	44.38	40.46	62.57	Significant	3.66e-03
5	14	11	9	34	25	56.0	44.0	37.05	73.35	9.26	18.34	Significant	2.99e-03
6	1	2	2	5	3	33.3	66.7	5.63	79.76	0.17	2.39		1.00e+00
7	1	1	0	2	2	50.0	50.0	9.45	90.55	0.19	1.81		7.45e-01

[31] Significance calculated compared to baseline of average for reviews from female reviewers (20.8%)

Table D4 (for Figure 16): Breakdown of reviews by reviewer gender and chemistry sub-discipline

Chemistry sub-discipline	Female	Male	Unknown	Total For Level	Total Gender Deduced	Female Percentage	Male Percentage	LCI	UCI	LCI Count	UCI Count	Significance[32]	Adjusted P-Value
Analytical	31448	100835	128803	261086	132283	23.8	76.2	23.54	24.00	31145.55	31752.46	Significant	2.90e-146
Biological	21260	65876	66613	153749	87136	24.4	75.6	24.11	24.68	21012.50	21509.46	Significant	2.98e-140
Catalysis	15153	70456	96631	182240	85609	17.7	82.3	17.45	17.96	14935.36	15373.12	Significant	6.09e-110
Chemical Biology and Medicinal	22356	70708	75888	168952	93064	24.0	76.0	23.75	24.30	22101.56	22612.44	Significant	4.04e-120
Energy	20466	84826	164926	270218	105292	19.4	80.6	19.20	19.68	20215.50	20718.84	Significant	4.98e-23
Environmental	10627	30112	39434	80173	40739	26.1	73.9	25.66	26.51	10454.21	10801.62	Significant	2.12e-139
Food	11075	24504	28422	64001	35579	31.1	68.9	30.65	31.61	10904.56	11246.89	Significant	1.76e-319
Inorganic	18906	60053	47845	126804	78959	23.9	76.1	23.65	24.24	18671.98	19142.02	Significant	1.29e-96
Materials	46370	183372	288303	518045	229742	20.2	79.8	20.02	20.35	45994.08	46748.21	Significant	6.39e-08
Miscellaneous	529	1709	1101	3339	2238	23.6	76.4	21.92	25.44	490.63	569.40		1.00e+00
Nanoscience	35833	136450	248849	421132	172283	20.8	79.2	20.61	20.99	35503.94	36164.31		1.00e+00
Organic	10371	58987	49590	118948	69358	15.0	85.0	14.69	15.22	10188.27	10556.42	Significant	6.85e-319
Physical	27293	125224	138222	290739	152517	17.9	82.1	17.70	18.09	27000.83	27587.63	Significant	5.09e-173
NA	98	408	297	803	506	19.4	80.6	16.15	23.04	81.73	116.60		1.00e+00

[32] Significance calculated compared to baseline of average for reviews from female reviewers (20.8%)

Table E1a (for Figure 17): Multinomial GLM model of proportion of review recommendations and corresponding author gender

Corresponding author gender	Reviewer major decision	fit (Proportion of reviews)	Standard Error	LCI	UCI
F	accept	0.212	0.0018	0.209	0.216
M	accept	0.221	0.0010	0.219	0.223
F	minor.revision	0.288	0.0020	0.284	0.291
M	minor.revision	0.304	0.0011	0.302	0.306
F	major.revision	0.206	0.0017	0.203	0.210
M	major.revision	0.192	0.0009	0.190	0.194
F	reject	0.294	0.0020	0.290	0.298
M	reject	0.283	0.0011	0.281	0.286

Table E2 (for Figure 18): Multinomial GLM model of proportion of first round review recommendations and reviewer gender

Reviewer gender	Reviewer major decision	fit (Proportion of reviews)	Standard Error	LCI	UCI
F	accept	0.226	0.0019	0.222	0.230
M	accept	0.217	0.0010	0.215	0.219
F	minor.revision	0.300	0.0021	0.296	0.304
M	minor.revision	0.300	0.0011	0.298	0.302
F	major.revision	0.213	0.0019	0.209	0.217
M	major.revision	0.191	0.0009	0.189	0.192
F	reject	0.261	0.0020	0.257	0.265
M	reject	0.292	0.0011	0.290	0.294

Table E3 (for Figure 19): Multinomial GLM model of proportion of review recommendations, corresponding author gender and reviewer gender

Reviewer gender	Corresponding author gender	Reviewer major decision	fit (Proportion of reviews)	Standard Error	LCI	UCI
F	F	accept	0.116	0.110	0.122	0.00317
F	F	major.revision	0.261	0.252	0.269	0.00434
F	F	minor.revision	0.318	0.309	0.327	0.00460
F	F	reject	0.305	0.296	0.314	0.00455
F	M	accept	0.128	0.124	0.131	0.00199
F	M	major.revision	0.243	0.238	0.248	0.00256
F	M	minor.revision	0.322	0.317	0.328	0.00279
F	M	reject	0.308	0.302	0.313	0.00276
M	F	accept	0.106	0.103	0.109	0.00168
M	F	major.revision	0.231	0.227	0.236	0.00231
M	F	minor.revision	0.308	0.303	0.313	0.00253
M	F	reject	0.354	0.349	0.360	0.00262
M	M	accept	0.126	0.124	0.128	0.00096
M	M	major.revision	0.214	0.212	0.217	0.00119
M	M	minor.revision	0.329	0.327	0.332	0.00136
M	M	reject	0.331	0.328	0.334	0.00136

Table F1a: Breakdown of submissions by corresponding author gender and final outcome

Final outcome	Female	Male	Unknown	Total For Level	Total Gender Deduced	Female Percentage	Male Percentage	LCI	UCI	LCI Count	UCI Count	Significance[33]	Adjusted P-Value
accepted	35206	118443	174039	327688	153649	22.9	77.1	22.70	23.12	34884.16	35529.92	Significant	1.25e-14
rejected	38130	114749	227835	380714	152879	24.9	75.1	24.73	25.16	37799.39	38462.54	Significant	3.21e-16
revise	125	424	464	1013	549	22.8	77.2	19.45	26.46	106.79	145.29		1.00e+00
undecided	845	2694	4154	7693	3539	23.9	76.1	22.50	25.31	796.30	895.70		1.00e+00

[33] Significance calculated compared to baseline of average for original submissions from female corresponding authors (23.9%)

Table F1b: Breakdown of submissions by first author gender and final outcome

Final outcome	Female	Male	Unknown	Total For Level	Total Gender Deduced	Female Percentage	Male Percentage	LCI	UCI	LCI Count	UCI Count	Significance[34]	Adjusted P-Value
accepted	51007	103217	173464	327688	154224	33.1	66.9	32.84	33.31	50645.53	51369.78		1
rejected	53971	105477	221266	380714	159448	33.8	66.2	33.62	34.08	53601.29	54341.95		1
revise	175	332	506	1013	507	34.5	65.5	30.51	38.76	154.67	196.51		1
undecided	1118	2413	4162	7693	3531	31.7	68.3	30.15	33.22	1064.55	1172.86		1

[34] Significance calculated compared to baseline of average for original submissions from female first authors (33.4%)

Table F2a: Breakdown of reviews by reviewer gender and whether the reviewer recommendation agreed with the Editor's status

StautsAndRecommendationAgree	Female	Male	Unknown	Total For Level	Total Gender Deduced	Female Percentage	Male Percentage	LCI	UCI	LCI Count	UCI Count	Significance[35]	Adjusted P-Value
Agree	78845	298073	365499	742417	376918	20.9	79.1	20.79	21.05	78356.71	79335.53		1
Disagree	16118	63674	81841	161633	79792	20.2	79.8	19.92	20.48	15896.86	16341.43		1

[35] Significance calculated compared to baseline of average for reviews from female reviewers (20.8%)

Table F2b (for Figure 20a): Binomial GLM model of "Status And Recommendation Agree" variable, reviewer gender and corresponding author gender

Reviewer gender	Corresponding author gender	fit[36]	Standard Error	LCI	UCI	Population Size
F	F	0.834	0.024	0.830	0.838	12810
M	F	0.834	0.014	0.831	0.837	34620
F	M	0.827	0.013	0.825	0.830	40868
M	M	0.828	0.007	0.827	0.829	144873

[36] Model fit: StatusAndRecommendationAgree ~ ReviewerGender * CorrespondingAuthorGender

Table F2c (for Figure 20b): Binomial GLM model of "Status And Recommendation Agree" variable, reviewer gender and editor gender

Reviewer gender	Editor gender	fit[37]	Standard Error	LCI	UCI	Population Size
F	F	0.840	0.017	0.836	0.843	24416
M	F	0.833	0.016	0.830	0.836	27395
F	M	0.820	0.009	0.818	0.822	77755
M	M	0.834	0.008	0.832	0.835	117564

[37] Model fit: StatusAndRecommendationAgree ~ ReviewerGender * CorrespondingAuthorGender

Table F3a (for Figure 21): Breakdown of accepted submissions by corresponding author gender and chemistry sub-discipline

Chemistry sub-discipline	Female	Male	Unknown	Total For Level	Total Gender Deduced	Female Percentage	Male Percentage	LCI	UCI	LCI Count	UCI Count	Significance[38]	Adjusted P-Value
Analytical	15372	42251	57222	114845	57623	26.7	73.3	26.32	27.04	15165	15581	Significant	5.46e-49
Biological	11113	29220	33631	73964	40333	27.6	72.4	27.12	27.99	10938	11290	Significant	1.20e-59
Catalysis	6608	26243	47088	79939	32851	20.1	79.9	19.69	20.55	6467	6752	Significant	6.44e-56
Chemical Biology and Medicinal	11759	30769	37698	80226	42528	27.7	72.3	27.23	28.08	11579	11941	Significant	1.72e-66
Energy	8801	34096	76723	119620	42897	20.5	79.5	20.14	20.90	8638	8966	Significant	4.74e-58
Environmental	5343	12324	19602	37269	17667	30.2	69.8	29.57	30.92	5224	5463	Significant	2.80e-78
Food	5188	9828	14728	29744	15016	34.5	65.5	33.79	35.31	5074	5303	Significant	6.49e-185
Inorganic	6146	23898	22122	52166	30044	20.5	79.5	20.00	20.92	6010	6284	Significant	1.68e-41
Materials	23220	77945	134972	236137	101165	23.0	77.0	22.69	23.21	22959	23483	Significant	1.30e-07
Miscellaneous	287	934	738	1959	1221	23.5	76.5	21.21	25.97	258.98	317.04		1.00e+00
Nanoscience	17713	55679	119826	193218	73392	24.1	75.9	23.83	24.45	17487	17941		1.00e+00
Organic	4911	23062	22931	50904	27973	17.6	82.4	17.11	18.01	4788	5037	Significant	9.56e-142
Physical	14380	53127	66430	133937	67507	21.3	78.7	20.99	21.61	14173	14590	Significant	4.97e-53

[38] Significance calculated compared to baseline of average for original submissions from female corresponding authors (23.9%)

Figure and Table F3b: Breakdown of accepted submissions by first author gender and chemistry sub-discipline

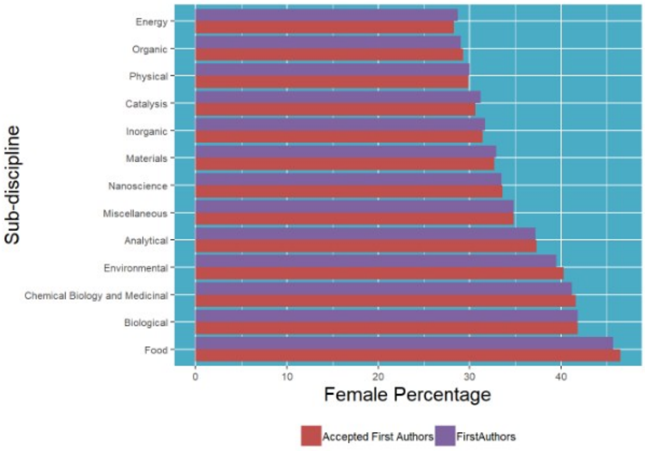

Chemistry sub-discipline	Female	Male	Unknown	Total For Level	Total Gender Deduced	Female Percentage	Male Percentage	LCI	UCI	LCI Count	UCI Count	Significance[39]	Adjusted P-Value
Analytical	21673	36364	56808	114845	58037	37.3	62.7	36.95	37.74	21445.10	21901.88	Significant	7.12e-84
Biological	16672	23188	34104	73964	39860	41.8	58.2	41.34	42.31	16479.30	16865.33	Significant	2.02e-264
Catalysis	10289	23285	46365	79939	33574	30.6	69.4	30.15	31.14	10124.18	10455.30	Significant	1.59e-22
Chemical Biology and Medicinal	17609	24721	37896	80226	42330	41.6	58.4	41.13	42.07	17410.57	17808.07	Significant	4.60e-266
Energy	12452	31564	75604	119620	44016	28.3	71.7	27.87	28.71	12267.63	12638.04	Significant	6.22e-113
Environmental	7249	10759	19261	37269	18008	40.3	59.7	39.54	40.97	7120.40	7378.35	Significant	4.87e-78
Food	7190	8257	14297	29744	15447	46.5	53.5	45.76	47.33	7068.64	7311.62	Significant	5.37e-246
Inorganic	9294	20299	22573	52166	29593	31.4	68.6	30.88	31.94	9138.23	9451.20	Significant	8.27e-09
Materials	33512	68899	133726	236137	102411	32.7	67.3	32.44	33.01	33218.37	33806.95		4.37e-01
Miscellaneous	417	781	761	1959	1198	34.8	65.2	32.16	37.55	385.31	449.86		1.00e+00
Nanoscience	25632	50677	116909	193218	76309	33.6	66.4	33.26	33.93	25376.92	25888.34		1.00e+00
Organic	8135	19646	23123	50904	27781	29.3	70.7	28.75	29.82	7987.14	8284.45	Significant	1.56e-44
Physical	20068	47150	66719	133937	67218	29.9	70.1	29.51	30.20	19836.24	20301.31	Significant	2.62e-81

[39] Significance calculated compared to baseline of average for original submissions from female first authors (33.4%)

Table F3c (for Figure 22): Binomial GLM model of corresponding author gender of accepted submissions, chemistry sub-discipline and journal impact factor

Impact factor (IF 2017)	Chemistry sub-discipline (cats)	fit[40]	Standard Error	LCI	UCI	Population Size
1.6	Analytical	0.329	0.017	0.324	0.334	17694
1.6	Biological	0.336	0.021	0.330	0.342	9076
1.6	Catalysis	0.228	0.026	0.223	0.234	7863
1.6	Chemical Biology and Medicinal	0.327	0.020	0.321	0.332	11403
1.6	Energy	0.230	0.023	0.224	0.235	9270
1.6	Environmental	0.329	0.025	0.322	0.336	9176
1.6	Food	0.400	0.027	0.392	0.408	6028
1.6	Inorganic	0.263	0.030	0.256	0.271	2889
1.6	Materials	0.267	0.014	0.264	0.271	23023
1.6	Miscellaneous	0.391	0.095	0.362	0.420	436
1.6	Nanoscience	0.284	0.017	0.280	0.288	17162
1.6	Organic	0.226	0.028	0.219	0.232	6192
1.6	Physical	0.240	0.018	0.236	0.244	11861
10	Analytical	0.162	0.033	0.157	0.168	2523
10	Biological	0.186	0.034	0.180	0.193	2812
10	Catalysis	0.174	0.032	0.168	0.180	3914
10	Chemical Biology and Medicinal	0.198	0.034	0.191	0.205	2703
10	Energy	0.194	0.021	0.190	0.199	10875
10	Environmental	0.250	0.052	0.238	0.263	851
10	Food	0.213	0.066	0.199	0.227	423
10	Inorganic	0.127	0.050	0.120	0.134	2256
10	Materials	0.191	0.018	0.187	0.195	12079
10	Miscellaneous	0.039	0.257	0.028	0.053	111
10	Nanoscience	0.204	0.020	0.200	0.208	8413
10	Organic	0.113	0.045	0.108	0.119	2406
10	Physical	0.181	0.027	0.176	0.187	5553
13	Analytical	0.122	0.048	0.116	0.129	65
13	Biological	0.147	0.051	0.139	0.155	50
13	Catalysis	0.157	0.049	0.149	0.165	24
13	Chemical Biology and Medicinal	0.162	0.051	0.153	0.171	27
13	Energy	0.183	0.033	0.177	0.189	219
13	Environmental	0.225	0.076	0.209	0.242	21
13	Food	0.163	0.096	0.147	0.181	13
13	Inorganic	0.096	0.076	0.087	0.104	38
13	Materials	0.168	0.028	0.163	0.173	506
13	Miscellaneous	0.015	0.365	0.009	0.023	0
13	Nanoscience	0.180	0.031	0.174	0.186	359
13	Organic	0.087	0.068	0.080	0.094	2
13	Physical	0.164	0.040	0.157	0.171	244
4.5	Analytical	0.262	0.010	0.260	0.265	22997
4.5	Biological	0.278	0.012	0.275	0.281	16595
4.5	Catalysis	0.208	0.015	0.205	0.211	12179
4.5	Chemical Biology and Medicinal	0.278	0.011	0.275	0.280	17938
4.5	Energy	0.217	0.014	0.214	0.220	9729
4.5	Environmental	0.300	0.017	0.295	0.305	4469
4.5	Food	0.328	0.020	0.322	0.334	6243
4.5	Inorganic	0.208	0.015	0.205	0.211	18110
4.5	Materials	0.239	0.008	0.237	0.241	35428
4.5	Miscellaneous	0.198	0.083	0.181	0.215	354
4.5	Nanoscience	0.254	0.009	0.252	0.257	19799
4.5	Organic	0.180	0.016	0.177	0.183	11503
4.5	Physical	0.218	0.010	0.216	0.220	32659
7.4	Analytical	0.205	0.020	0.201	0.209	12709
7.4	Biological	0.226	0.021	0.222	0.231	9004
7.4	Catalysis	0.189	0.019	0.186	0.193	7784
7.4	Chemical Biology and Medicinal	0.233	0.020	0.228	0.238	8338
7.4	Energy	0.205	0.014	0.202	0.208	9043
7.4	Environmental	0.273	0.033	0.265	0.281	2689
7.4	Food	0.263	0.042	0.253	0.274	1567
7.4	Inorganic	0.161	0.029	0.156	0.166	6048
7.4	Materials	0.213	0.011	0.210	0.215	25862
7.4	Miscellaneous	0.086	0.167	0.071	0.105	291
7.4	Nanoscience	0.227	0.012	0.224	0.230	24554
7.4	Organic	0.142	0.027	0.137	0.146	7311
7.4	Physical	0.198	0.016	0.195	0.201	14697

[40] Model fit: CorrespondingAuthorGender ~cats * IF2017

Figure and Table F3d: Binomial GLM model of first author gender of accepted submissions, chemistry sub-discipline and journal impact factor

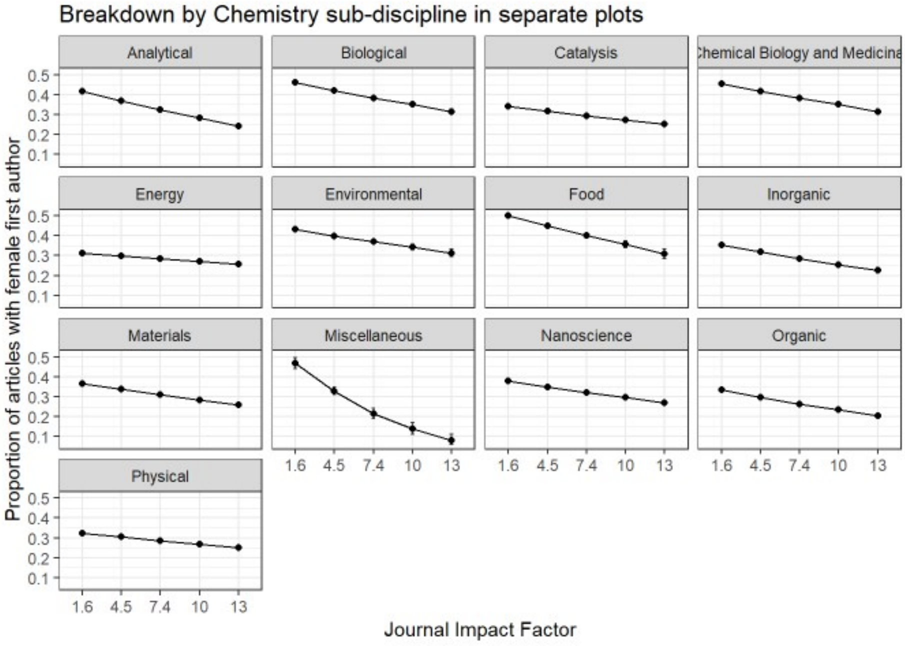

Impact factor (IF 2017)	Chemistry sub-discipline (cats)	fit[41]	Standard Error	LCI	UCI	Population Size
1.6	Analytical	0.418	0.015	0.413	0.423	18623
1.6	Biological	0.460	0.019	0.454	0.466	9101
1.6	Catalysis	0.341	0.022	0.335	0.347	8502
1.6	Chemical Biology and Medicinal	0.455	0.018	0.449	0.461	11536
1.6	Energy	0.311	0.020	0.305	0.316	10024
1.6	Environmental	0.430	0.023	0.423	0.437	9744
1.6	Food	0.502	0.025	0.494	0.510	6250
1.6	Inorganic	0.353	0.026	0.346	0.361	3054
1.6	Materials	0.367	0.012	0.363	0.371	25141
1.6	Miscellaneous	0.468	0.089	0.440	0.496	428
1.6	Nanoscience	0.378	0.015	0.374	0.383	18939
1.6	Organic	0.335	0.024	0.329	0.342	6300
1.6	Physical	0.324	0.016	0.319	0.328	12481
10	Analytical	0.283	0.029	0.276	0.291	2262
10	Biological	0.350	0.030	0.341	0.359	2511
10	Catalysis	0.272	0.027	0.266	0.279	4051
10	Chemical Biology and Medicinal	0.350	0.030	0.341	0.358	2469
10	Energy	0.271	0.019	0.267	0.276	11342
10	Environmental	0.343	0.048	0.329	0.356	913
10	Food	0.356	0.059	0.339	0.374	375
10	Inorganic	0.255	0.041	0.245	0.265	2154
10	Materials	0.284	0.016	0.280	0.288	12036
10	Miscellaneous	0.139	0.188	0.112	0.170	106
10	Nanoscience	0.296	0.017	0.291	0.300	8955
10	Organic	0.234	0.036	0.226	0.242	2253
10	Physical	0.268	0.024	0.262	0.274	5383
13	Analytical	0.242	0.043	0.232	0.252	72
13	Biological	0.314	0.045	0.302	0.326	56
13	Catalysis	0.250	0.042	0.240	0.260	24
13	Chemical Biology and Medicinal	0.315	0.045	0.302	0.327	37
13	Energy	0.258	0.029	0.251	0.265	184
13	Environmental	0.313	0.069	0.295	0.333	21
13	Food	0.309	0.085	0.286	0.333	15
13	Inorganic	0.225	0.063	0.211	0.239	31
13	Materials	0.258	0.024	0.252	0.264	445

[41] Model fit: Model: FirstAuthorGender ~ Category * ImpactFactor. ANOVA: Category is highly significant (p = 0.00e00); ImpactFactor is highly significant (p = 8.36e-261); Category*ImpactFactor interactions are highly significant (p = 8.87e-22).

13	Miscellaneous	0.081	0.270	0.059	0.111	0
13	Nanoscience	0.269	0.027	0.262	0.276	328
13	Organic	0.203	0.053	0.193	0.215	2
13	Physical	0.250	0.036	0.241	0.259	246
4.5	Analytical	0.369	0.009	0.366	0.371	23173
4.5	Biological	0.421	0.011	0.418	0.424	16422
4.5	Catalysis	0.316	0.013	0.313	0.320	12045
4.5	Chemical Biology and Medicinal	0.418	0.010	0.415	0.421	17910
4.5	Energy	0.297	0.013	0.293	0.300	9688
4.5	Environmental	0.399	0.016	0.394	0.404	4428
4.5	Food	0.450	0.018	0.445	0.456	6482
4.5	Inorganic	0.317	0.013	0.314	0.321	17907
4.5	Materials	0.337	0.007	0.335	0.339	34986
4.5	Miscellaneous	0.329	0.066	0.310	0.348	371
4.5	Nanoscience	0.349	0.008	0.346	0.351	20298
4.5	Organic	0.298	0.013	0.294	0.302	11310
4.5	Physical	0.304	0.009	0.302	0.306	31981
7.4	Analytical	0.322	0.017	0.317	0.327	12355
7.4	Biological	0.383	0.018	0.378	0.388	9002
7.4	Catalysis	0.293	0.016	0.288	0.297	7768
7.4	Chemical Biology and Medicinal	0.381	0.018	0.376	0.387	8260
7.4	Energy	0.283	0.012	0.280	0.286	9113
7.4	Environmental	0.369	0.030	0.360	0.378	2453
7.4	Food	0.400	0.037	0.388	0.411	1603
7.4	Inorganic	0.283	0.024	0.277	0.290	5777
7.4	Materials	0.309	0.010	0.306	0.311	25792
7.4	Miscellaneous	0.214	0.120	0.189	0.241	265
7.4	Nanoscience	0.320	0.010	0.317	0.323	24835
7.4	Organic	0.263	0.021	0.258	0.268	7366
7.4	Physical	0.285	0.014	0.281	0.289	14693

Table F4a (for Figure 23): Breakdown of accepted submissions by corresponding author gender and final number of revisions

Final number of revisions	Female	Male	Unknown	Total For Level	Total Gender Deduced	Female Percentage	Male Percentage	LCI	UCI	LCI Count	UCI Count	Significance[42]	Adjusted P-Value
0	1300	4780	5900	11980	6080	21.4	78.6	20.37	22.43	1238.44	1363.76	Significant	2.04e-02
1	24709	85294	122212	232215	110003	22.5	77.5	22.22	22.71	24438.77	24981.35	Significant	2.37e-24
2	7877	24488	38794	71159	32365	24.3	75.7	23.87	24.81	7726.68	8029.29		1.00e+00
3	1071	3263	5995	10329	4334	24.7	75.3	23.45	26.02	1016.32	1127.62		1.00e+00
4	217	536	968	1721	753	28.8	71.2	25.70	32.16	193.49	242.13		1.00e+00
5	32	82	170	284	114	28.1	71.9	20.61	36.96	23.50	42.13		1.00e+00

[42] Significance calculated compared to baseline of average for original submissions from female corresponding authors (23.9%)

Figure and Table F4b: Breakdown of accepted submissions by first author gender and final number of revisions

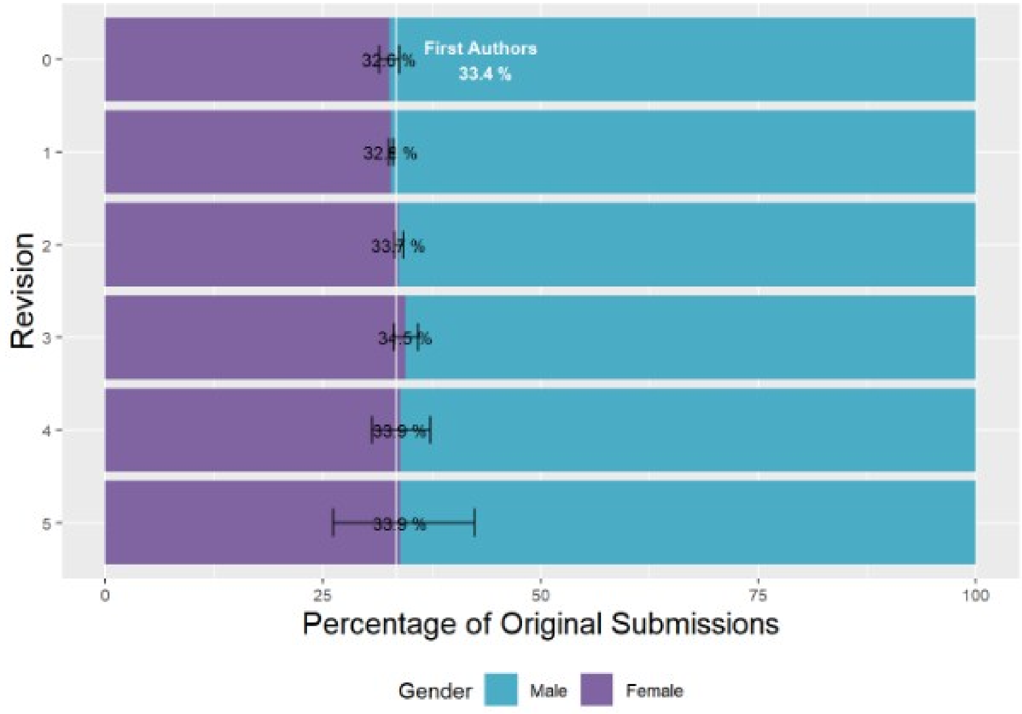

Final number of revisions	Female	Male	Unknown	Total For Level	Total Gender Deduced	Female Percentage	Male Percentage	LCI	UCI	LCI Count	UCI Count	Significance[43]	Adjusted P-Value
0	1968	4064	5948	11980	6032	32.6	67.4	31.45	33.82	1897.31	2040.02		1
1	36176	74007	122032	232215	110183	32.8	67.2	32.56	33.11	35871.14	36482.17		1
2	10976	21553	38630	71159	32529	33.7	66.3	33.23	34.26	10809.49	11143.76		1
3	1587	3008	5734	10329	4595	34.5	65.5	33.18	35.92	1524.44	1650.75		1
4	257	501	963	1721	758	33.9	66.1	30.62	37.35	232.12	283.11		1
5	43	84	157	284	127	33.9	66.1	26.20	42.47	33.27	53.93		1

[43] Significance calculated compared to baseline of average for original submissions from female first authors (33.4%)

Table G1a (for Figure 24): Gender breakdown of corresponding and first authors of cited and citing articles of citations in comparison to those of all publications

Author type	Female	Male	Unknown	Total For Level	Total Gender Deduced	Female Percentage	Male Percentage	LCI	UCI	LCI Count	UCI Count	Significance[44]	Adjusted P-Value
RSC Published Articles' Corresponding Authors	35206	118443	174039	327688	153649	22.9	77.1	22.7	23.12	34884.16	35529.92	Significant	0
RSC Citing RSC Articles' Corresponding Authors	11132	43606	86335	141073	54738	20.3	79.7	20	20.68	10948.57	11317.71	Significant	0
RSC Cited RSC Articles' Corresponding Authors	10906	48379	81788	141073	59285	18.4	81.6	18.09	18.71	10722.31	11092.12	Significant	0
RSC Published RSC Articles' First Authors	51007	103217	173464	327688	154224	33.1	66.9	32.84	33.31	50645.53	51369.78	Significant	0
RSC Citing RSC Articles' First Authors	17659	39193	84221	141073	56852	31.1	68.9	30.68	31.44	17443.48	17875.98	Significant	0
RSC Cited RSC Articles' First Authors	18160	41865	81048	141073	60025	30.3	69.7	29.89	30.62	17940.18	18381.33	Significant	0

[44] Significance calculated compared to baseline of average for all authors of original submissions to the RSC (35.8%)

Table G1b (for Figure 25): Breakdown of articles' corresponding author gender by number of RSC citations to it

Number of RSC citations	Female	Male	Unknown	Total For Level	Total Gender Deduced	Female Percentage	Male Percentage	LCI	UCI	LCI Count	UCI Count	Significance[45]	Adjusted P-Value
1	3073	11735	19505	34313	14808	20.8	79.2	20.11	21.41	2977.40	3170.85	Significant	5.05e-09
2	1970	7838	12270	22078	9808	20.1	79.9	19.30	20.89	1893.38	2048.92		1.99e-01
3	1347	5463	8607	15417	6810	19.8	80.2	18.85	20.74	1283.73	1412.59		1.00e+00
4	808	3984	6556	11348	4792	16.9	83.1	15.83	17.95	758.46	860.08		1.00e+00
5	650	3045	4960	8655	3695	17.6	82.4	16.40	18.85	605.87	696.62		1.00e+00
6	492	2274	3984	6750	2766	17.8	82.2	16.41	19.26	453.81	532.67		1.00e+00
7	427	1764	3332	5523	2191	19.5	80.5	17.88	21.20	391.82	464.52		1.00e+00
8	248	1408	2824	4480	1656	15.0	85.0	13.34	16.78	220.85	277.83		4.34e-01
9	198	1188	2322	3708	1386	14.3	85.7	12.54	16.23	173.80	224.94		7.84e-02
10	180	990	1860	3030	1170	15.4	84.6	13.43	17.57	157.10	205.55		1.00e+00
11	231	693	1760	2684	924	25.0	75.0	22.31	27.89	206.18	257.74	Significant	5.82e-04
12	120	744	1356	2220	864	13.9	86.1	11.74	16.36	101.41	141.36		4.42e-01
13	91	793	1157	2041	884	10.3	89.7	8.45	12.48	74.72	110.32	Significant	2.92e-08
14	112	490	854	1456	602	18.6	81.4	15.69	21.92	94.47	131.93		1.00e+00
15	75	570	975	1620	645	11.6	88.4	9.37	14.34	60.42	92.51	Significant	2.24e-03
16	64	480	848	1392	544	11.8	88.2	9.31	14.76	50.64	80.28	Significant	1.71e-02
17	102	493	680	1275	595	17.1	82.9	14.32	20.39	85.21	121.30		1.00e+00
18	108	486	504	1098	594	18.2	81.8	15.28	21.49	90.77	127.66		1.00e+00
19	38	285	418	741	323	11.8	88.2	8.66	15.76	27.99	50.92		4.94e-01
20	80	120	520	720	200	40.0	60.0	33.46	46.92	66.92	93.84	Significant	2.71e-10
21	0	252	357	609	252	0.0	100.0	-0.31	1.81	NaN	NaN	Significant	2.49e-20
22	66	110	506	682	176	37.5	62.5	30.68	44.85	54.00	78.94	Significant	4.13e-07
23	0	138	391	529	138	0.0	100.0	-0.55	3.26	NaN	NaN	Significant	2.02e-10
24	48	96	264	408	144	33.3	66.7	26.14	41.39	37.65	59.60	Significant	2.88e-03
25	75	100	300	475	175	42.9	57.1	35.75	50.27	62.57	87.97	Significant	1.54e-11
26	0	208	182	390	208	0.0	100.0	-0.37	2.18	NaN	NaN	Significant	1.34e-16
27	27	54	135	216	81	33.3	66.7	24.00	44.17	19.44	35.78		1.09e-01
28	0	280	112	392	280	0.0	100.0	-0.28	1.63	NaN	NaN	Significant	9.01e-23
29	29	232	261	522	261	11.1	88.9	7.81	15.54	20.38	40.57		4.59e-01
30	30	90	90	210	120	25.0	75.0	18.07	33.48	21.69	40.17		1.00e+00
31	31	93	310	434	124	25.0	75.0	18.17	33.33	22.53	41.33		1.00e+00
32	0	0	192	192	0	0.0	0.0	0.00	0.00	0.00	0.00		0.00e+00
33	33	66	297	396	99	33.3	66.7	24.80	43.11	24.56	42.68	Significant	3.72e-02
34	0	170	204	374	170	0.0	100.0	-0.45	2.66	NaN	NaN	Significant	3.16e-13
35	0	70	105	175	70	0.0	100.0	-1.03	6.23	NaN	NaN	Significant	8.59e-05
36	0	72	144	216	72	0.0	100.0	-1.00	6.07	NaN	NaN	Significant	5.61e-05
37	37	111	148	296	148	25.0	75.0	18.69	32.58	27.66	48.21		1.00e+00
38	0	114	152	266	114	0.0	100.0	-0.66	3.92	NaN	NaN	Significant	2.11e-08
39	0	39	117	156	39	0.0	100.0	-1.71	10.68	NaN	NaN	Significant	2.39e-02
40	0	120	40	160	120	0.0	100.0	-0.63	3.73	NaN	NaN	Significant	5.62e-09
41	0	0	123	123	0	0.0	0.0	0.00	0.00	0.00	0.00		0.00e+00
42	0	0	42	42	0	0.0	0.0	0.00	0.00	0.00	0.00		0.00e+00
43	0	43	43	86	43	0.0	100.0	-1.58	9.78	NaN	NaN	Significant	1.07e-02
45	0	0	180	180	0	0.0	0.0	0.00	0.00	0.00	0.00		0.00e+00
46	0	46	46	92	46	0.0	100.0	-1.49	9.20	NaN	NaN	Significant	7.53e-03
47	0	0	47	47	0	0.0	0.0	0.00	0.00	0.00	0.00		0.00e+00
48	0	48	96	144	48	0.0	100.0	-1.44	8.85	NaN	NaN	Significant	4.97e-03
49	0	98	98	196	98	0.0	100.0	-0.76	4.53	NaN	NaN	Significant	3.74e-07
50	0	50	100	150	50	0.0	100.0	-1.39	8.52	NaN	NaN	Significant	3.29e-03
51	51	0	51	102	51	100.0	0.0	91.63	101.36	46.73	51.70	Significant	1.63e-36
52	0	52	52	104	52	0.0	100.0	-1.34	8.22	NaN	NaN	Significant	2.18e-03
55	0	110	0	110	110	0.0	100.0	-0.68	4.05	NaN	NaN	Significant	2.94e-08
56	0	56	0	56	56	0.0	100.0	-1.26	7.68	NaN	NaN	Significant	9.58e-04
57	0	57	0	57	57	0.0	100.0	-1.24	7.55	NaN	NaN	Significant	1.01e-03
58	0	58	0	58	58	0.0	100.0	-1.22	7.43	NaN	NaN	Significant	6.37e-04
59	0	0	118	118	0	0.0	0.0	0.00	0.00	0.00	0.00		0.00e+00
61	0	61	61	122	61	0.0	100.0	-1.16	7.09	NaN	NaN	Significant	4.35e-04
63	0	63	0	63	63	0.0	100.0	-1.13	6.88	NaN	NaN	Significant	2.86e-04
64	0	64	0	64	64	0.0	100.0	-1.12	6.78	NaN	NaN	Significant	3.12e-04
65	65	65	0	130	130	50.0	50.0	41.53	58.47	53.99	76.01	Significant	4.71e-14
68	0	68	68	136	68	0.0	100.0	-1.06	6.40	NaN	NaN	Significant	1.32e-04
74	0	0	74	74	0	0.0	0.0	0.00	0.00	0.00	0.00		0.00e+00
76	0	0	76	76	0	0.0	0.0	0.00	0.00	0.00	0.00		0.00e+00
77	0	0	77	77	0	0.0	0.0	0.00	0.00	0.00	0.00		0.00e+00
81	0	0	81	81	0	0.0	0.0	0.00	0.00	0.00	0.00		0.00e+00
82	0	0	82	82	0	0.0	0.0	0.00	0.00	0.00	0.00		0.00e+00
87	0	0	87	87	0	0.0	0.0	0.00	0.00	0.00	0.00		0.00e+00
88	0	0	88	88	0	0.0	0.0	0.00	0.00	0.00	0.00		0.00e+00
89	0	89	0	89	89	0.0	100.0	-0.83	4.96	NaN	NaN	Significant	1.97e-06
93	0	93	0	93	93	0.0	100.0	-0.79	4.76	NaN	NaN	Significant	8.48e-07
135	0	0	135	135	0	0.0	0.0	0.00	0.00	0.00	0.00		0.00e+00
178	0	0	178	178	0	0.0	0.0	0.00	0.00	0.00	0.00		0.00e+00
186	0	0	186	186	0	0.0	0.0	0.00	0.00	0.00	0.00		0.00e+00

[45] Significance calculated compared to baseline of average for all corresponding authors of cited articles, 18.4%

Table G2 (for Figure 26): Binomial GLM model of citation success of published articles and corresponding author gender controlled by whether the article was unanimously accepted in its first revision.

CorrespondingAuthorGender	UnanimousAccept	fit[46]	Standard Error	LCI	UCI	Population Size
F	Not unanimous accept	0.0354	0.0240	0.0344	0.0365	50764
M	Not unanimous accept	0.0445	0.0123	0.0439	0.0452	155831
F	Unanimous accept	0.1671	0.0308	0.1617	0.1727	7564
M	Unanimous accept	0.1972	0.0150	0.1942	0.2003	28122

[46] Model fit: Cited ~ UnanimousAccept * CorrAuthGender

Table G3 (for Figure 27): Breakdown of cited corresponding author gender of citations by the cited article's publication year

Cited article year	Female	Male	Unknown	Total For Level	Total Gender Deduced	Female Percentage	Male Percentage	LCI	UCI	LCI Count	UCI Count	Significance[47]	Adjusted P-Value
2012	5212	24115	37803	67130	29327	17.8	82.2	17.34	18.21	5084.92	5341.55		1.000
2013	2635	12269	19723	34627	14904	17.7	82.3	17.08	18.30	2544.95	2727.53		1.000
2014	767	3255	6183	10205	4022	19.1	80.9	17.89	20.31	719.35	817.02		1.000
2015	671	2579	5296	8546	3250	20.6	79.4	19.29	22.07	626.90	717.35		1.000
2016	689	2617	5267	8573	3306	20.8	79.2	19.49	22.26	644.35	735.89		1.000
2017	673	2537	5376	8586	3210	21.0	79.0	19.59	22.41	628.91	719.32		0.716
2018	259	1007	2140	3406	1266	20.5	79.5	18.33	22.77	232.00	288.27		1.000

[47] Significance calculated compared to baseline of average for all corresponding authors of cited articles, 18.4%

Table G4 (for Figure 28): Breakdown of gender of de-duplicated authors of all citing articles and number of self-citations in RSC citation data set

RSC self-citation count	Female	Male	Unknown	Total For Level	Total Gender Deduced	Female Percentage	Male Percentage	LCI	UCI	LCI Count	UCI Count	Significance[48]	Adjusted P-Value
0	8990	19880	33803	62673	28870	31.1	68.9	30.61	31.68	8836.52	9144.93	Significant	1.43e-319
1	4812	11629	19071	35512	16441	29.3	70.7	28.58	29.97	4698.46	4927.13	Significant	3.11e-246
2	836	2306	4337	7479	3142	26.6	73.4	25.09	28.18	788.36	885.43	Significant	3.60e-26
3	253	809	1700	2762	1062	23.8	76.2	21.36	26.48	226.80	281.20	Significant	1.12e-02
4	112	416	764	1292	528	21.2	78.8	17.93	24.91	94.69	131.51		1.00e+00
5	59	197	479	735	256	23.0	77.0	18.29	28.60	46.83	73.21		1.00e+00
6	29	120	273	422	149	19.5	80.5	13.86	26.60	20.65	39.63		1.00e+00
7	26	68	173	267	94	27.7	72.3	19.59	37.48	18.41	35.24		1.00e+00
8	9	57	145	211	66	13.6	86.4	7.12	24.15	4.70	15.94		1.00e+00
9	13	36	82	131	49	26.5	73.5	16.10	40.37	7.89	19.78		1.00e+00
10	7	30	64	101	37	18.9	81.1	9.17	34.51	3.39	12.77		1.00e+00
11	5	15	40	60	20	25.0	75.0	10.81	47.25	2.16	9.45		1.00e+00
12	5	18	36	59	23	21.7	78.3	9.23	42.33	2.12	9.74		1.00e+00
13	0	13	26	39	13	0.0	100.0	-3.78	26.59	NaN	NaN		1.00e+00
14	0	11	21	32	11	0.0	100.0	-4.14	30.02	NaN	NaN		1.00e+00
15	2	9	14	25	11	18.2	81.8	3.99	48.85	0.44	5.37		1.00e+00
16	3	9	15	27	12	25.0	75.0	8.27	53.85	0.99	6.46		1.00e+00
17	1	2	10	13	3	33.3	66.7	5.63	79.76	0.17	2.39		1.00e+00
18	1	5	10	16	6	16.7	83.3	1.14	58.22	0.07	3.49		1.00e+00
19	0	4	6	10	4	0.0	100.0	-5.61	54.60	NaN	NaN		1.00e+00
20	1	5	6	12	6	16.7	83.3	1.14	58.22	0.07	3.49		1.00e+00
21	1	1	4	6	2	50.0	50.0	9.45	90.55	0.19	1.81		6.68e-01
22	2	3	2	7	5	40.0	60.0	11.60	77.09	0.58	3.85		1.00e+00
23	1	3	4	8	4	25.0	75.0	3.41	71.09	0.14	2.84		1.00e+00
24	2	3	2	7	5	40.0	60.0	11.60	77.09	0.58	3.85		1.00e+00
25	3	4	3	10	7	42.9	57.1	15.75	75.02	1.10	5.25		8.52e-01
26	0	1	2	3	1	0.0	100.0	-3.90	83.25	NaN	NaN		1.00e+00
27	0	1	1	2	1	0.0	100.0	-3.90	83.25	NaN	NaN		1.00e+00
28	0	0	3	3	0	0.0	0.0	0.00	0.00	0.00	0.00		0.00e+00
29	0	1	0	1	1	0.0	100.0	-3.90	83.25	NaN	NaN		1.00e+00
30	0	0	1	1	0	0.0	0.0	0.00	0.00	0.00	0.00		0.00e+00
33	0	0	1	1	0	0.0	0.0	0.00	0.00	0.00	0.00		0.00e+00
35	0	1	0	1	1	0.0	100.0	-3.90	83.25	NaN	NaN		1.00e+00
36	0	0	1	1	0	0.0	0.0	0.00	0.00	0.00	0.00		0.00e+00
37	1	0	2	3	1	100.0	0.0	16.75	103.90	0.17	1.04		1.84e-01
40	0	1	1	2	1	0.0	100.0	-3.90	83.25	NaN	NaN		1.00e+00
41	0	0	1	1	0	0.0	0.0	0.00	0.00	0.00	0.00		0.00e+00
56	1	0	0	1	1	100.0	0.0	16.75	103.90	0.17	1.04		1.84e-01

[48] Significance calculated compared to baseline of average for all corresponding authors of cited articles, 18.4%

Table G5: Breakdown of cited corresponding author gender of citations and citing corresponding author gender

Citing corresponding Author Gender	Female	Male	Unknown	Total For Level	Total Gender Deduced	Female Percentage	Male Percentage	LCI	UCI	LCI Count	UCI Count	Significance[49]	Adjusted P-Value
F	959	3721	5161	9841	4680	20.5	79.5	19.36	21.67	906.01	1014.25		1
M	3321	15431	18450	37202	18752	17.7	82.3	17.17	18.26	3219.78	3424.70		1
U	5335	22823	48204	76362	28158	18.9	81.1	18.49	19.41	5207.31	5465.08		1

[49] Significance calculated compared to baseline of average for all corresponding authors of cited articles, 18.4%

Table G6a (for Figure 29): Gender breakdown of cited corresponding authors of citations by chemistry sub-discipline in comparison to that of corresponding authors of all accepted submissions

Chemistry sub-discipline	Female	Male	Unknown	Total For Level	Total Gender Deduced	Female Percentage	Male Percentage	LCI	UCI	LCI Count	UCI Count	Significance[50]	Adjusted P-Value
Analytical	2291	8168	12809	23268	10459	21.9	78.1	21.12	22.71	2209	2375	Significant	1.60e-15
Biological	1223	4161	4409	9793	5384	22.7	77.3	21.62	23.85	1164	1284	Significant	1.07e-11
Catalysis	1535	6543	12014	20092	8078	19.0	81.0	18.16	19.87	1467	1605		1.00e+00
Chemical Biology and Medicinal	1387	4627	4992	11006	6014	23.1	76.9	22.02	24.14	1324	1452	Significant	7.43e-16
Energy	1963	10570	22308	34841	12533	15.7	84.3	15.04	16.31	1885	2044	Significant	9.43e-12
Environmental	476	1598	2399	4473	2074	23.0	77.0	21.19	24.81	440	515	Significant	4.28e-04
Food	299	852	1009	2160	1151	26.0	74.0	23.53	28.59	271	329	Significant	2.64e-07
Inorganic	1490	7946	8964	18400	9436	15.8	84.2	15.07	16.54	1422	1561	Significant	2.61e-07
Materials	4511	19549	34905	58965	24060	18.7	81.3	18.26	19.25	4394	4631		1.00e+00
Miscellaneous	74	481	554	1109	555	13.3	86.7	10.74	16.43	60	91		9.73e-01
Nanoscience	3110	12493	30212	45815	15603	19.9	80.1	19.31	20.57	3013	3209	Significant	1.60e-02
Organic	1232	6120	6618	13970	7352	16.8	83.2	15.92	17.63	1171	1296		1.00e+00
Physical	2140	10274	14518	26932	12414	17.2	82.8	16.58	17.91	2059	2224		1.00e+00
NA	441	1657	4106	6204	2098	21.0	79.0	19.33	22.82	406	479		1.00e+00

[50] Significance calculated compared to baseline of average for original submissions from female corresponding authors (23.9%)

Table G6b (for Figure 30): Binomial GLM model of cited corresponding author gender of citations, chemistry sub-discipline and journal impact factor

Impact factor (IF 2017)	Chemistry sub-discipline (cats)	fit[51]	Standard Error	LCI	UCI	Population Size
1.6	Analytical	0.271	0.054	0.258	0.285	995
1.6	Biological	0.309	0.080	0.288	0.332	349
1.6	Catalysis	0.217	0.072	0.202	0.233	632
1.6	Chemical Biology and Medicinal	0.299	0.069	0.281	0.318	543
1.6	Energy	0.148	0.070	0.137	0.159	826
1.6	Environmental	0.234	0.099	0.212	0.257	487
1.6	Food	0.337	0.126	0.302	0.374	176
1.6	Inorganic	0.171	0.070	0.159	0.184	257
1.6	Materials	0.225	0.041	0.216	0.234	1637
1.6	Miscellaneous	0.174	0.244	0.133	0.223	48
1.6	Nanoscience	0.228	0.052	0.216	0.240	1186
1.6	Organic	0.210	0.069	0.196	0.225	608
1.6	Physical	0.219	0.055	0.207	0.231	724
10	Analytical	0.160	0.076	0.148	0.174	596
10	Biological	0.142	0.103	0.127	0.159	428
10	Catalysis	0.180	0.065	0.168	0.193	782
10	Chemical Biology and Medicinal	0.184	0.092	0.167	0.202	479
10	Energy	0.197	0.053	0.186	0.208	1721
10	Environmental	0.247	0.145	0.214	0.283	89
10	Food	0.165	0.220	0.130	0.208	41
10	Inorganic	0.133	0.090	0.120	0.147	860
10	Materials	0.179	0.045	0.171	0.187	2033
10	Miscellaneous	0.141	0.266	0.105	0.188	108
10	Nanoscience	0.220	0.050	0.209	0.231	1349
10	Organic	0.157	0.071	0.145	0.169	862
10	Physical	0.140	0.074	0.129	0.152	941
13	Analytical	0.131	0.116	0.115	0.149	9
13	Biological	0.104	0.160	0.086	0.124	4
13	Catalysis	0.168	0.105	0.150	0.187	4
13	Chemical Biology and Medicinal	0.152	0.142	0.130	0.177	1
13	Energy	0.217	0.086	0.199	0.237	25
13	Environmental	0.252	0.216	0.204	0.308	2
13	Food	0.124	0.327	0.085	0.177	1
13	Inorganic	0.122	0.140	0.104	0.142	4
13	Materials	0.164	0.071	0.152	0.177	73
13	Miscellaneous	0.131	0.412	0.082	0.203	0
13	Nanoscience	0.217	0.079	0.200	0.235	47
13	Organic	0.141	0.112	0.125	0.159	0
13	Physical	0.118	0.114	0.104	0.134	22
4.5	Analytical	0.228	0.026	0.222	0.234	3592
4.5	Biological	0.241	0.039	0.232	0.250	1614
4.5	Catalysis	0.204	0.040	0.196	0.212	2096
4.5	Chemical Biology and Medicinal	0.255	0.036	0.246	0.264	1985
4.5	Energy	0.163	0.041	0.156	0.171	1906
4.5	Environmental	0.238	0.059	0.225	0.252	601
4.5	Food	0.269	0.073	0.251	0.288	523
4.5	Inorganic	0.157	0.034	0.151	0.163	4220
4.5	Materials	0.208	0.022	0.203	0.213	6578
4.5	Miscellaneous	0.162	0.140	0.139	0.188	167
4.5	Nanoscience	0.225	0.028	0.219	0.231	2878
4.5	Organic	0.190	0.038	0.183	0.198	1900
4.5	Physical	0.188	0.028	0.183	0.194	4915
7.4	Analytical	0.190	0.043	0.182	0.199	3660
7.4	Biological	0.184	0.058	0.173	0.195	1852
7.4	Catalysis	0.191	0.037	0.184	0.198	2574
7.4	Chemical Biology and Medicinal	0.215	0.053	0.204	0.227	1488
7.4	Energy	0.180	0.033	0.174	0.187	2563
7.4	Environmental	0.243	0.089	0.222	0.264	414
7.4	Food	0.210	0.133	0.183	0.239	226
7.4	Inorganic	0.144	0.050	0.136	0.152	2362
7.4	Materials	0.192	0.026	0.187	0.197	5884
7.4	Miscellaneous	0.151	0.161	0.126	0.179	134
7.4	Nanoscience	0.222	0.028	0.216	0.229	4722
7.4	Organic	0.172	0.042	0.165	0.180	2451
7.4	Physical	0.161	0.043	0.154	0.169	2697

Table H1 (for Figures 31): Breakdown of living Chemists with highest H-index ranking by gender and H-index

[51] Model fit: CitedCorrAuthorGender ~cats * IF2017

H-index range	Female	Male	Unknown	Total For Level	Total Gender Deduced	Female Percentage	Male Percentage	LCI	UCI	LCI Count	UCI Count	Significance[52]	Adjusted P-Value
055-059	9	87	0	96	96	9.4	90.6	4.82	17.06	4.62	16.38	Significant	5.33e-07
060-064	4	110	0	114	114	3.5	96.5	1.08	8.97	1.23	10.22	Significant	1.35e-14
065-069	4	70	0	74	74	5.4	94.6	1.72	13.50	1.27	9.99	Significant	8.16e-08
070-074	0	72	0	72	72	0.0	100.0	-1.00	6.07	NaN	NaN	Significant	1.67e-12
075-079	0	57	0	57	57	0.0	100.0	-1.24	7.55	NaN	NaN	Significant	8.87e-10
080-084	1	34	0	35	35	2.9	97.1	-0.77	15.81	-0.27	5.53	Significant	2.72e-04
085-089	0	29	0	29	29	0.0	100.0	-2.18	13.87	NaN	NaN	Significant	1.37e-04
090-094	0	18	0	18	18	0.0	100.0	-3.08	20.67	NaN	NaN	Significant	1.24e-02
095-099	1	9	0	10	10	10.0	90.0	-0.39	42.60	-0.04	4.26		1.00e+00
100-104	0	14	0	14	14	0.0	100.0	-3.62	25.15	NaN	NaN	Significant	4.75e-02
105-109	0	9	0	9	9	0.0	100.0	-4.55	34.46	NaN	NaN		2.82e-01
110-114	0	5	0	5	5	0.0	100.0	-5.46	48.91	NaN	NaN		8.38e-01
115-119	0	4	0	4	4	0.0	100.0	-5.61	54.60	NaN	NaN		1.00e+00
120-124	0	3	0	3	3	0.0	100.0	-5.60	61.75	NaN	NaN		1.00e+00
125-129	0	3	0	3	3	0.0	100.0	-5.60	61.75	NaN	NaN		1.00e+00
130-134	0	1	0	1	1	0.0	100.0	-3.90	83.25	NaN	NaN		1.00e+00
140-144	0	2	0	2	2	0.0	100.0	-5.22	70.98	NaN	NaN		1.00e+00
145 or greater	0	1	0	1	1	0.0	100.0	-3.90	83.25	NaN	NaN		1.00e+00

[52] Significance calculated compared to baseline of average for all authors of original submissions to the RSC (35.8%)
