# Supplementary material for: Is there a gender gap in chemical sciences scholarly communication?
Source: Chem Sci. 2020 Jan 28;11(8):2277–301. doi: 10.1039/c9sc04090k (PMC7057853; doi:10.1039/c9sc04090k)
Supplement: Supplementary file 1 [file SC-011-C9SC04090K-s001.pdf]

Electronic Supplementary Material (ESI) for Chemical Science.
This journal is © The Royal Society of Chemistry 2020

C9SC04090K – Editor and Reviewer Details

Alán Aspuru-Guzik
Associate Editor, University of Toronto, Canada

Flaminio Squazzoni
Reviewer 1, University of Milan, Italy

Miriam B. Goodman
Reviewer 2, Stanford University, USA

Misha Teplitskiy
Reviewer 3, University of Michigan, USA

Quick Access

Round 1

Round 1 – Decision Letter 2

Round 1 – Reviewers' Comments 4

Round 1 – Authors' Responses 11

Round 2

Round 2 – Decision Letter 13

Round 2 – Reviewers' Comments 14

Round 2 – Authors' Responses 18

Round 3

Round 3 – Decision Letter 21

Round 1 – Decision Letter

19-Sep-2019

Dear Dr Day:

I have received the reviews for this unique paper. As you can see, there are major methodological concerns of reviewer #3 that I think should be addressed with care. The 54-figure comment is something that I already had noticed. Can you consolidate in multi-panel and bring it down in number? Sending some less relevant ones to the SI if necessary?

I am not an expert in social science statistics but I see that the 3 reviewers have comments in this regard. I recommend that before resubmission you consider the points raised and maybe run by another expert in the subfield.

Best,
Alan

Manuscript ID: SC-EDG-08-2019-004090
TITLE: Is there a gender bias in Chemical Sciences scholarly communication?

Thank you for your submission to Chemical Science, published by the Royal Society of Chemistry. I sent your manuscript to reviewers and I have now received their reports which are copied below.

I have carefully evaluated your manuscript and the reviewers' reports, and the reports indicate that major revisions are necessary.

Please submit a revised manuscript which addresses all of the reviewers' comments. Further peer review of your revised manuscript may be needed. When you submit your revised manuscript please include a point by point response to the reviewers' comments and highlight the changes you have made. Full details of the files you need to submit are listed at the end of this email.

Please submit your revised manuscript as soon as possible using this link:

You should submit your revised manuscript as soon as possible; please note you will receive a series of automatic reminders. If your revisions will take a significant length of time, please contact me. If I do not hear from you, I may withdraw your manuscript from consideration and you will have to resubmit. Any resubmission will receive a new submission date.

The Royal Society of Chemistry requires the submitting author to provide their ORCID iD when they submit a revised manuscript. This is quick and easy to do as part of the revised manuscript submission process. We will publish this information with the article, and you may choose to have your ORCID record updated automatically with details of the publication.

Please also encourage your co-authors to sign up for their own ORCID account and associate it with their account on our manuscript submission system. Please note that we are unable to do this on behalf of your co-authors. For further information see: http://www.rsc.org/journals-books-databases/journal-authors-reviewers/processes-policies/#attribution-id.

I look forward to receiving your revised manuscript.

Yours sincerely,

Alán Aspuru-Guzik
Professor of Chemistry and Computer Science
Faculty Member Vector Institute for Artificial Intelligence
University of Toronto

Associate Editor, Chemical Science
80 St George St, Toronto ON M5S 2H6 aspuru@utoronto.ca

Round 1 – Reviewers' Comments

Reviewer 1

Recommendation: Revisions required

Comments:
This is a nice paper on gender bias in scholarly journals with original data. Results are interesting. However, data and analysis must be improved to fully support authors' findings.

While paper categorisation does not have any problematic issue, the method used to estimate gender of authors, reviewers and editors could implicitely bias findings. While authors used US and UK data and adjusted data manually (though details here are not sufficient to understand the procedure), it is likely that names from certain countries/ethnical groups were systematically underestimated or missatributed. Completing data minining by using different web sources, such as http://namespedia.com/ and other available sources (e.g., Nameguess (gpeters.com), Nameberry.com, indiachildnames.com), could help ensure that data mining did not systematically underrepresent women and men from certain groups. On the other hand, using manual coders from a variety of different countries and languages could help minimize such a bias. Even a control on a subsample of random cases of uncertain gender attribution from assistants from different countries and cultural origins could help verify whether such a potential bias has been present. Note that while I agreed on the idea of removing any unknown name and concentrate on attributions with a consisent probability, this could mean that authors from non Western orogins could be dramatically mis-represented and this could have an effect on the estimation of gender effects. This problem cannot be simply solved by assuming a normal distribution of men and women amonst under-represented ethnics.

While GLM is a robust and well-established statistical technique, here the predictive power of the model relies on a complex set of interactions and conditions which can confound the established correlations.

First, you have different journals with different number of submissions, IF, peer review models. Second, any manuscript has a different number of authors and there is research showing a strong correlation between the number of co-authors and the likelihood of acceptance/rejection. Concentrating on the corresponding authors without controlling for the number of authors can compromise the results of the analysis as you could end up comparing - for instance - a manuscript submitted by a solo women with a manuscript submitted by a men with 5 female coauthors. Adding controls on your regressions by considering mixed effects models is the most appropriate approach. You could for instance add random effects on journals.

When considering editorial decisions in subsequent rounds, it is important to rule out the influence of n number of peer review rounds as in these cases, you have learning effects (e.g., reviewers learning from other reviewers). It would be better to concentrate on the first round of peer review and the first editorial decision to capture the 'true' gender bias and then present the fate of manuscripts in subsequent rounds separately.

Another control could be the gender composition of authors and isolating the solo men/solo women alone/team manuscripts. A model comparing the probability of positive/negative editorial decision in the first round could clearn a lot of confounders.
In short, at the moment, your model could compare: a manuscript submitted by a women alone to the journal X, which received a few submissions, in a period of low IF with a manuscript submitted

by 25 male authors to the journal Y, which received tons of submissions and is more selective, in a period of high IF etc. With this setp-up, it is hard to determine that it's the gender alone that explains different gender rates of success. Furthermore, if you add the gatekeeping function of reviewers, you add another layer of complexification. To turn to the example, you are comparing the former manuscript (with the previous charecteristics) which received a higher review score (all acceptance recommendation) with the latter (with all its previous characteristics) but receiving a low review score (all rejections). Is it the gender of the author that predicts the editorial decision or the quality of the manuscripts as perceved by reviewers?

This same problem can affect the analysis on citations. Citations seem not being weighted by journal, year, and issue. Time and journal prestige matter for citations and comparison between articles must be controlled for time and compared coherently.

Self-citations consider only the name identity of corresponding authors. This underestimates the number of self-citations to co-authored articles, as admitted by authors themselves. Howevwer, this could lead to underestimate the self-citation propensities of men who might typically co-author more articles and have larger teams. It is also unclear whether authors have restricted their attentions to records published in the very same journals or made an extensive analysis on a larger dataset to reconstruct self-citation patterns across journals.

Regarding H-index, considering that records are time dependent, it would be essential to weight the index by age, e.g., by considering the range between the year of the first and last publication. It is reasonable to suppose that women were more present among the last generations of scholars. It would be nice if you would compare men and women in the same time window, to check if gender differences in recognition have reduced over time.

Additional Questions:
How would you rate this article?:
Significant (10-25% - not suitable for publication in Chemical Science)

It is the responsibility of authors to provide fully convincing evidence for the homogeneity, purity and identity of all compounds they claim as new. Is adequate supporting information provided to support the claims made in this manuscript?:
Yes

Do the references contain appropriate and balanced citations?:
No

What contribution does this article make to the subject area it addresses? (1 = poor; 10 = outstanding; only articles rated 9 or 10 are suitable for Chemical Science, articles rated 8 or lower suggest the work is unsuitable for Chemical Science):
9

Reviewer 2

Recommendation: Revisions required

Comments:
Day, Corbett, and Boyle have undertaken a comprehensive analysis of scholarly publishing in chemistry under the Royal Society of Chemistry. The data collection and analysis reported in this manuscript is impressive and the datasets themselves have the potential to drive positive change in the scholarly publishing landscape, especially with respect to gender distribution, diversity, and the potential for gender bias in publishing. The manuscript would be easier to read and have a bigger impact if it provided readers with a more clearly defined point of view. The primary rationale for collecting the data seems to be that it hasn't been done in Chemistry. Do the authors believe that chemistry differs from other communities of science? Do the data support such a difference? As noted below, because the RSC publishes many titles this data set can take on the relationship between gender and impact factor, including submission rates. This is an aspect of this data set that differs from others addressing gender bias in scholarly publication and the authors might consider emphasizing this as a key rationale for performing the analysis. Below, I provide additional suggestions to improve the presentation of the results, commentaries on the methodology, and critiques of some of implicit assumptions underpinning the report.

The authors establish the 'baseline female percentage for chemistry researchers' using data from a number of sources, including sources that would not be available to other authors (i.e. membership in the RSC and the pool of authors submitted to RSC journals). It is simply terrific that the fraction of female chemistry researchers was determined from complimentary, but disparate sources. It is also excellent to know that there is broad agreement among these sources, arriving at a baseline rate of 35.8% of chemistry researchers are female.

Without stating it explicitly, the authors assume that comparison to this metric suffices to answer the question posed in the title. Regardless of the outcome of the subsequent analysis, however, the match (or mismatch) between the baseline rate and the proportion of female authors is not sufficient by itself to establish whether or not gender bias has or has not influenced the selection (prescreening decisions), review, or citation process. What we need to know in addition is the success rate as a function of gender. In particular, is there a gender gap in the proportion of manuscripts with female vs. male first (& last) authors that are advanced to the peer-review stage (not prescreened)? Does such a gap in success rate exists for manuscripts accepted for publication? In citation rates after publication? A study of submissions to eLife (Murray et al, available on bioRxiv) analyzes success rates and reports that submissions with male corresponding (last) authors are significantly more likely to advance to the peer review stage and to be accepted for publication than those with female corresponding authors. In principle, such a gender gap in success rates could arise even if the proportion of proportion of published articles with female corresponding (last) matched the baseline rate of female chemistry investigators. Similarly, a gender gap in success rates might be absent even if the proportion of articles submitted with female corresponding authors were less than the baseline rate. I hope these examples suffice to illustrate the value of analyzing success rates and the limitations of comparing the proportion of female authors vs. the baseline rate. Ideally, the authors should undertake the additional analysis of success rates as a function of author gender. At a minimum, they should make their analytical assumptions about the meaning of the mismatch between the baseline rate and the proportion of female corresponding explicit and discuss the limitations of using this comparison as a proxy for the presence or absence of gender bias in the review or citation process.

The method used to estimate gender from names is state-of-the-art and relies on a now widely used software package. Because it relies on databases of Western names, this approach performs poorly for non-Western names. It is terrific that the authors dig into this deeper and inform the reader that a large number of RSC submissions originate with research groups in China and the poor performance of the gender estimator affects the analysis. A quick and dirty analysis of the performance of the gender estimator indicates that it provides reasonable gender estimates for only 62% of authors, 57% of reviewers, but 74% of editors. What is the impact of this limitation of the gender-name estimator on the analysis, especially its disproportionate impact on submissions from non-Western countries?

• The manuscript has 54 figures! This is a very large number of figures and impairs the reader’s experience. Some of the figures should be consolidated or transformed into summary tables and the underlying source data disseminated along with the manuscript. I found the use of the red color to indicate female scientists and blue to indicate male scientists irksome.

• The finding that the proportion of female authors is inversely proportional to journal impact factor (Figures 14, 15, 40, 41) is important. The ability to perform this analysis should receive a greater emphasis in the introduction. The finding should be emphasized in the discussion from both a policy perspective and from the reliance on impact factor as a proxy for excellence and impact in the evaluation of individual researchers. A similar result has been reported in a preprint analyzing published peer-reviewed papers in neuroscience. (https://www.biorxiv.org/content/10.1101/275362v2.full)

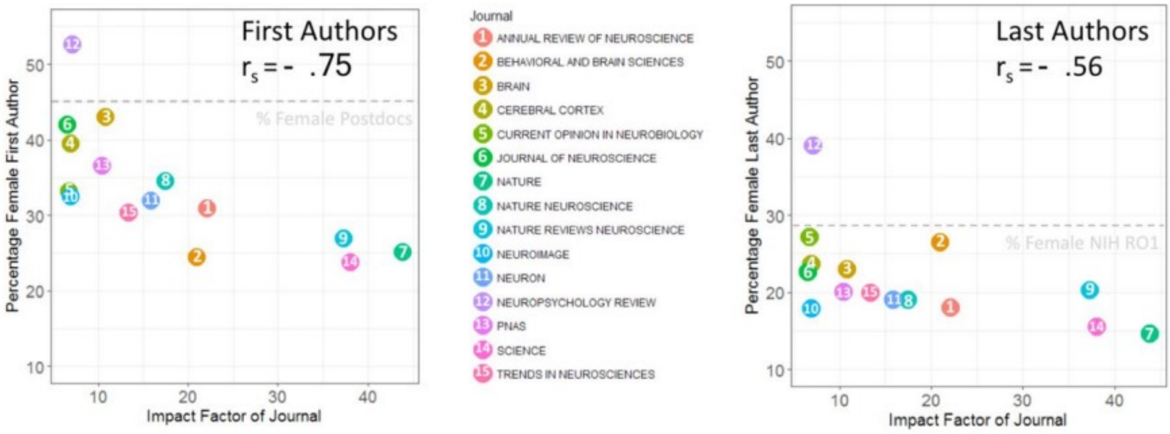

• Please report the time frame of the analysis in the abstract and introduction. It would also be helpful to know the number of submissions and publications included in the analysis. These metadata help readers and perhaps policy makers assess the statistical power of the study.

• Although it may be typical style for this journal and type of article, Table 1 is distracting. Removing it and reducing the number of subheads would improve readability.

• (p. 4). “…people with unknown gender were discounted from the percentage calculation.” The word “discounted” has unfortunate implications with respect to worth and its use here implies that the people whose gender could not be inferred were worth less than those whose gender could be inferred from their names. Consider replacing “discounted” with “omitted”.

• The term ‘prescreened’ is confusing and might be publishing jargon. From context, I infer that this is a euphemism for manuscripts that are evaluated at the editorial level and rejected prior to peer review. It would be better if the term were explicitly defined.

Additional Questions:
How would you rate this article?:
Significant (10-25% - not suitable for publication in Chemical Science)

It is the responsibility of authors to provide fully convincing evidence for the homogeneity, purity and identity of all compounds they claim as new. Is adequate supporting information provided to support the claims made in this manuscript?:

Do the references contain appropriate and balanced citations?:
Yes

What contribution does this article make to the subject area it addresses? (1 = poor; 10 = outstanding; only articles rated 9 or 10 are suitable for Chemical Science, articles rated 8 or lower suggest the work is unsuitable for Chemical Science):
7

Reviewer 3

Recommendation: Reject

Comments:
This manuscript presents a thorough description of the publication processes at Royal Society of Chemistry journals, focusing on how outcomes in each step of the process differ for men and women. It also considers how the broader chemical community cites the works by men and women the journals ultimately publish. Peer review processes are highly consequential but secretive, so these data are very unique and of unusually large scale. As the title indicates, the goal is to locate gender biases, if any, in publishing and in the future rectify them via policies targeted to the relevant stage of publishing. Keeping this goal in mind, I will focus on the biggest issue -- can this analysis inform policy recommendations? -- and make smaller comments about the analysis itself.

Can this analysis inform policy recommendations?

The big issue in studies of bias, gender or other kinds, is identification – usually there are multiple mechanisms that can lead to an outcome, so can the analysis identify/isolate the one of interest? Here, author gender may be correlated with other features of their submissions (e.g. quality), so, if we find disparate outcomes, it is unclear whether they were caused by author gender or these other features. Indeed, biases earlier in the research process that favor men, such as student recruitment (https://www.pnas.org/content/109/41/16474.short) or grant amounts (https://jamanetwork.com/journals/jama/article-abstract/2726973) can enable men to submit manuscripts of higher quality to journals. On the other hand, men and women might hold themselves to different standards when submitting, with women submitting higher quality works (http://www.erinhengel.com/research/publishing_female.pdf, also Figure 19 in manuscript seems to support this). So, we have many reasons to expect submission quality to differ by gender (in any direction), so it is crucial to control for this if we want to identify that publishing disparities are driven by gender.

The ideal way to do this is a research design that keeps quality constant

1.a. "Audit"-style experiments, where the name on the submissions is experimentally manipulated. The only gender-related study of this type I'm aware of finds no bias: https://www.nature.com/articles/s41562-018-0517-y

1.b. Designs that utilize multiple reviewers per manuscript, so all features of the manuscript are controlled for, e.g. https://www.sciencedirect.com/science/article/pii/S0048733318301598

2. A very imperfect but common way of controlling for quality is by adding to the statistical models variables that we think might proxy for quality, e.g. previous citation counts, professional status, etc.

I was surprised that the authors followed none of these strategies. In the social science literature on this topic, (2) is considered the bare minimum, and only somewhat suggestive, with (1b) being used sometimes and (1a) very rarely. So to strengthen the conclusions we can draw from these data I'd suggest the authors pursue at least (2). It would also be interesting to do (1b), and seeing whether male and female reviewers *of the same manuscript* evaluate it differently when it's authored by men/women. The latter analysis is still imperfect, because no different would not necessarily imply no bias -- gender-related biases are generally society-wide and may bias men *and* women similarly.

Without some approach to control for quality and other submission characteristics (journal fit, momentum of topic, methodology) I'm afraid we really can't conclude much regarding gender bias in evaluation and citing, although gender differences in submission are still instructive.

Improving the analysis

In general, the authors use GLM (logit?) models. These are especially useful if one includes control variables, like the ones I mentioned above for quality. However, almost all the regressions do not use any controls. For just two variables, these models are therefore unnecessary and the authors can just compute raw percentages or crosstabs from the data; if anything, the models add unnecessary structure. But I'd instead recommend collecting other data on the submitters, especially anything plausibly related to quality / methodological approach / topic, and including it in the regressions.

Minor points

- My assumption is that all RSC journals are single-blind? I didn't actually find this mentioned in the paper.
- Pre-screening is what is elsewhere called "desk reject" stage, right? That might be a useful footnote somewhere.
- p.g. 7, top left: "By 'original submission' we mean first revisions of an article with a particular manuscript ID, so that a single original submission includes all revisions that …". I assume you didn't intend to put that latter clause there?
- Pg. 10, section B5: Coloring cells using the negative log10 of p-value struck me as an idiosyncratic choice, or at least one I've never come across before. Furthermore, the p-value will depend on sample size in each cell, so the coloration is really hard to interpret. I'd just choose something easy, like percent, with 100% = blue, 0% = red, and mark statistically significantly different cells with asterisks.
- Pg. 12, Figure 14 caption. Mentions just 1 p-value (p=3.75e-97) for what are many interactions, but surely that's not the p-value for each one?

Pg. 12, right middle. "However, while the mean impact factor of journals of female….". The stats in the parentheses seem off. That difference in means should be statistically significant if the very small standard errors are correctly reported.

Additional Questions:
How would you rate this article?: Routine (25-50% - not suitable for publication in Chemical Science)

It is the responsibility of authors to provide fully convincing evidence for the homogeneity, purity and identity of all compounds they claim as new. Is adequate supporting information provided to support the claims made in this manuscript?:

Do the references contain appropriate and balanced citations?:

What contribution does this article make to the subject area it addresses? (1 = poor; 10 = outstanding; only articles rated 9 or 10 are suitable for Chemical Science, articles rated 8 or lower suggest the work is unsuitable for Chemical Science):

Round 1 – Authors' Responses

Dear Professor Aspuru-Guzik,

Thankyou very much for your response to our submission "Is there a gender bias in Chemical Sciences scholarly communication?" and the referees comments – we very much appreciate the time that they took and feedback that they supplied. As a result, we have very carefully and extensively revised our paper to address their various comments as follows (we submit a separate document detailing how we've addressed each Referee's feedback in detail to elaborate on the points below too).

As suggested I have moved figures pertaining to First Author breakdowns to the ESI, removed some figures e.g. for redundant GLM models as suggested by referees, quoted more raw figures in the manuscript itself and consolidated some figures into multipanel figures and managed to bring down the total considerably to 32 (including the figures pertaining to the additional analyses requested by the reviewers).

Since the initial submission we consulted with Dr Pragya Agarwal, a social scientist academic and author with a background in gender and inclusivity issues about the paper.

We have addressed the methodological concerns by the following actions (which are discussed in more detail in responses to referees specific points where appropriate):

- Being more careful with our use of the term "gender bias". We acknowledge that we have more effectively shown a gender gap or imbalance which may have multiple causes, rather than presented evidence of a conscious or unconscious gender bias. Please note the change in title of this submission to "Is there a gender gap in Chemical Sciences scholarly communication?" amongst numerous other terminology changes. We have added discussion of the possible underlying issue of submission quality varying with gender in the conclusions.
- Investigated controls for quality e.g. single-author submissions, indicating submissions with unanimous referee decisions for initial revisions; and highlighting breakdowns by journal category and impact factor
- Adding a comparison to an alternative gender inference method and defining the scope of the paper more clearly
- Calculating success rates for publication stages to complement the binomial significance comparisons
- Redefining our "self-citation" definition in line with that suggested and recalculating that section
- Added an analysis of the age distribution of the chemists with the highest H-index in section to emphasise that the H-index method greatly favours researchers who are further on in their career.

We believe that we have addressed the concerns of the referees and that the article is improved as a result. We submit:
- IsThere AGenderGapInChemicalSciencesScholarlyCommunications.docx (the revised manuscript)
- ChangesHighlightedIsThere AGenderGapInChemicalSciencesScholarlyCommunications.docx (a manuscript which tracks all changes between revision 1 and revision 2, with figures removed)
- ESIIsThere AGenderGapInChemicalSciencesScholarlyCommunications.docx (electronic supplementary information)
- AddressingReviewersComments.docx (document which addresses each referees comments point by point and elaborates on what changes were made to the article)

We appreciate your further consideration of the paper, and that of the referees, and await your

decision about the revised manuscript.

Please note that during the process of our revisions we also decided that we would like to adjust the title to “Is there a gender gap in Chemical Sciences scholarly communication?” (and discuss this further in our responses to the referees).

Please also note that we have submitted a preprint of this latest revision of the article to ChemRxiv with the permission of the RSC.

Regards,
Aileen

Round 2 – Decision Letter

20-Dec-2019

Dear Dr Day:

Manuscript ID: SC-EDG-08-2019-004090.R1
TITLE: Is there a gender gap in Chemical Sciences scholarly communication?

Thank you for your submission to Chemical Science, published by the Royal Society of Chemistry. I sent your revised manuscript to one of the original reviewers and I have now received their report which is copied below.

After careful evaluation of your revised manuscript and the reviewer's report, I will be pleased to accept your manuscript for publication after further revisions.

Please revise your manuscript to fully address the reviewer's comments. When you submit your revised manuscript please include a point by point response to the reviewer's comments and highlight the changes you have made. Full details of the files you need to submit are listed at the end of this email.

Please submit your revised manuscript as soon as possible using this link :

You should submit your revised manuscript as soon as possible; please note you will receive a series of automatic reminders. If your revisions will take a significant length of time, please contact me. If I do not hear from you, I may withdraw your manuscript from consideration and you will have to resubmit. Any resubmission will receive a new submission date.

The Royal Society of Chemistry requires the submitting author to provide their ORCID iD when they submit a revised manuscript. This is quick and easy to do as part of the revised manuscript submission process. We will publish this information with the article, and you may choose to have your ORCID record updated automatically with details of the publication.

Please also encourage your co-authors to sign up for their own ORCID account and associate it with their account on our manuscript submission system. Please note that we are unable to do this on behalf of your co-authors. For further information see: http://www.rsc.org/journals-books-databases/journal-authors-reviewers/processes-policies/#attribution-id.

I look forward to receiving your revised manuscript.

Yours sincerely,
Alán Aspuru-Guzik
Professor of Chemistry and Computer Science
Faculty Member Vector Institute for Artificial Intelligence
University of Toronto

Associate Editor, Chemical Science
80 St George St, Toronto ON M5S 2H6 aspuru@utoronto.ca

Round 2 – Reviewers' Comments

Reviewer 3

Recommendation: Revisions required

Comments:
I commend the authors on what is clearly extensive effort in improving the manuscript and trying to address reviewers' several concerns. The change in title and language away from bias towards "gap" is helpful. I have just one issue remaining:

I still don't think enough was done to account for potential differences in quality of submissions. The authors attempted the following two strategies.

"method (1b) – using whether the reviewers decisions were unanimous as a control"

This choice is motivated by "Articles that are accepted with reviewer consensus might be expected to be less controversially higher quality papers than those where at least one reviewer suggested rejection or major revision" (pp. 6). I think the intuition may well be correct, but I don't see how it helps the goal of this paper, which is largely to explain outcomes of peer review, i.e. reviewers' recommendations, by author gender. So how can we use the outcome of peer review as also an input/explanatory variable? If there is gender bias favoring men, then they will get more consensus-accepts. If men submit manuscripts of higher quality, they will also get more consensus-accepts. So I don't think this approach gets us anywhere.

"method (2) – investigating whether single-authored papers might be an appropriate control"

The authors note that this approach also doesn't work.

The authors also note that ***"We're reluctant to use a proxy for quality such as previous citation count or professional status since we believe the quality should be judged from the submission itself rather than the history of its author (which might have been influenced by previous biases)."***

I am sympathetic to this line of thinking, and don't put too much credibility in studies that attempt to use such controls. However, using only variables endogeneous to the process just rearranges the dependent variable somewhat, it can't *explain* the process. So, without any controls for quality, I think one has to be circumscribed in what can be concluded from the data, especially given that the most credible piece of prior work finds no bias (Forcher et al. 2019, https://www.nature.com/articles/s41562-018-0517-y).

So what I recommend is

1. Get rid of the 2 attempted controls, method 1b and method 2. I don't think they help interpretation and axing them would help shorten the paper
2. In the Conclusion section, get rid of "…but have investigated using single-authored submissions (unsuccessfully) and unanimous referee decisions (more successfully) as controls which are proxies for quality. In all these cases (with or without controls) there are consistently lower female proportions of successful submissions in all groups." To a distracted reader (which is probably most us) the structure of this sentence makes it sound like some exogeneous information was brought to bear and it validated the interpretation of bias.

3. Add a small “disclaimer” sentence somewhere in the introduction, emphasizing that you are reporting gender gaps, but what explains these gaps is not entirely clear, and there is some debate in the literature. This would be a good place to cite the Forcher et al 2019 paper, unless there is some compelling reason not to (P.S. It’s not my paper)

With all these comments I am pushing the authors to soften their conclusions, particularly for readers who may be unfamiliar with this literature. But I don’t think the value of the paper is much diminished. It’s still a very valuable analysis, and I look forward to seeing it in print.

P.S. Minor point: In my first review I took issue with a p-value on Page 12, Figure 14. I think it’s now Figure 10 in the new draft. This may be a conventions issue across disciplines, but in my area, the syntax *corr_author_gender ~ category*impact_factor* usually means

Corr_author_gender = Beta1*category + Beta2*impact_factor + Beta3*category*impact_factor + error.

So if category is a categorical variable with many levels, there will be a separate intercept and separate slope for each one, and associated p-values with each. So it’s not clear what the one reported p-value was referring to, perhaps the p-value for the overall model fit? If it’s for the interactions, there should be a separate p-value for each interaction term (category-specific slope) and each intercept (category-specific intercept). Perhaps I’m misunderstanding the regression specification; if the authors have a different one in mind, I would recommend noting that in the paper somewhere

Additional Questions:
How would you rate this article?: Routine (25-50% - not suitable for publication in Chemical Science)

It is the responsibility of authors to provide fully convincing evidence for the homogeneity, purity and identity of all compounds they claim as new. Is adequate supporting information provided to support the claims made in this manuscript?:
Yes

Do the references contain appropriate and balanced citations?:
Yes

What contribution does this article make to the subject area it addresses? (1 = poor; 10 = outstanding; only articles rated 9 or 10 are suitable for Chemical Science, articles rated 8 or lower suggest the work is unsuitable for Chemical Science):
5

Reviewer 2

Recommendation: Accept

Comments:
In their revision, Day, Corbett & Boyle have successfully address the concerns and conceptually challenges that I raised in the initial round of review. The authors are to be commended for undertaking additional analyses of the underlying data (such as computing success rates) and for revising the language to more accurately represent the nuances of gender bias and disparities. These changes improve the reader's experience while also increasing the impact of the study on the research community.

In my view, it is especially important to emphasize that the results here support a conceptual model of compounding advantage to male chemists and disadvantage to their female counterparts. Within the context of this study, manuscripts submitted by female authors are not only less likely to advance to the review stage and those that cross this bridge are more likely to undergo multiple rounds of review, delaying the ultimate appearance of the peer reviewed publication. It is not hard to see how this might create the illusion of reduced productivity compared relative to male counterparts at fixed evaluation points (such as grant proposal review, appointment, or promotion).

Apart from this general comment, I would like to call out a few instances of odd phrasing that the authors might wish to address:
1. (p. 18) “Inclusion of names that are more gender ambiguous and could not be assigned a gender reliably, introduces other considerations, e.g. geographical, which we do not want to affect the significances calculated.” The final clause is self-evident, but the author’s desire to avoid this complication is distinct from its existence. Perhaps this sentence and those immediately preceding it could be revised to clarify the rationale for proceeding despite the limitations of the gender-name estimation procedure.
2. (p. 42) “We have seen small but significant gender-based differences during the publication process against female authors over the limited time range under investigation.” In revising this sentence to replace “bias” with “gender-based differences”, the authors left behind “against”. Delete “against female authors” and it reads well. Please also review the manuscript for other, similar instances of imperfect revision.

3. (p. 43) “Rather than one dominant factor, it is more akin to “death by a thousand cuts”. It is apparent that this female drop off through the publication process is most marked for female corresponding authors, but is also apparent to a lesser degree for female first authors.”

Additional Questions:
How would you rate this article?: Very significant (top 10% - suitable for publication in Chemical Science)

It is the responsibility of authors to provide fully convincing evidence for the homogeneity, purity and identity of all compounds they claim as new. Is adequate supporting information provided to support the claims made in this manuscript?:

Do the references contain appropriate and balanced citations?:
Yes

What contribution does this article make to the subject area it addresses? (1 = poor; 10 =

outstanding; only articles rated 9 or 10 are suitable for Chemical Science, articles rated 8 or lower suggest the work is unsuitable for Chemical Science):
9

Round 2 – Authors' Responses

Dear Professor Aspuru-Guzik,
Thankyou very much for your acceptance of our submission "Is there a gender gap in Chemical Sciences scholarly communication?" and for you and the referees taking extra time to review these changes. We have addressed their additional comments as follows. We await your decision on the final revised manuscript.
Regards,
Aileen

Referee 2 Comments
- In their revision, Day, Corbett & Boyle have successfully address the concerns and conceptually challenges that I raised in the initial round of review. The authors are to be commended for undertaking additional analyses of the underlying data (such as computing success rates) and for revising the language to more accurately represent the nuances of gender bias and disparities. These changes improve the reader's experience while also increasing the impact of the study on the research community.
In my view, it is especially important to emphasize that the results here support a conceptual model of compounding advantage to male chemists and disadvantage to their female counterparts. Within the context of this study, manuscripts submitted by female authors are not only less likely to advance to the review stage and those that cross this bridge are more likely to undergo multiple rounds of review, delaying the ultimate appearance of the peer reviewed publication. It is not hard to see how this might create the illusion of reduced productivity compared relative to male counterparts at fixed evaluation points (such as grant proposal review, appointment, or promotion).
AED> Thankyou for highlighting this point – we've made this point in a more toned-down way (in view of Referee 3's comments to soften our conclusions) in one of the final paragraphs of the paper.

- Apart from this general comment, I would like to call out a few instances of odd phrasing that the authors might wish to address:
- (p. 18) "Inclusion of names that are more gender ambiguous and could not be assigned a gender reliably, introduces other considerations, e.g. geographical, which we do not want to affect the significances calculated." The final clause is self-evident, but the author's desire to avoid this complication is distinct from its existence. Perhaps this sentence and those immediately preceding it could be revised to clarify the rationale for proceeding despite the limitations of the gender-name estimation procedure.
AED> We have rephrased this sentence and the paragraph around it.
- (p. 42) "We have seen small but significant gender-based differences during the publication process against female authors over the limited time range under investigation." In revising this sentence to replace "bias" with "gender-based differences", the authors left behind "against". Delete "against female authors" and it reads well. Please also review the manuscript for other, similar instances of imperfect revision.
AED> I have adjusted the instance highlighted and thoroughly read and checked the rest of the paper as a result of this and Referee 3's comments.
- (p. 43) "Rather than one dominant factor, it is more akin to "death by a thousand cuts". It is apparent that this female drop off through the publication process is most marked for female corresponding authors, but is also apparent to a lesser degree for female first authors."
AED> This has been tidied up.

Referee 3
- I commend the authors on what is clearly extensive effort in improving the manuscript and trying to address reviewers' several concerns. The change in title and language away from bias towards

“gap” is helpful. I have just one issue remaining:
- I still don’t think enough was done to account for potential differences in quality of submissions. The authors attempted the following two strategies.
- “method (1b) – using whether the reviewers decisions were unanimous as a control”
- This choice is motivated by “Articles that are accepted with reviewer consensus might be expected to be less controversially higher quality papers than those where at least one reviewer suggested rejection or major revision” (pp. 6). I think the intuition may well be correct, but I don’t see how it helps the goal of this paper, which is largely to explain outcomes of peer review, i.e. reviewers’ recommendations, by author gender. So how can we use the outcome of peer review as also an input/explanatory variable? If there is gender bias favoring men, then they will get more consensus-accepts. If men submit manuscripts of higher quality, they will also get more consensus-accepts. So I don’t think this approach gets us anywhere.
- “method (2) – investigating whether single-authored papers might be an appropriate control”
- The authors note that this approach also doesn’t work.
- The authors also note that “We’re reluctant to use a proxy for quality such as previous citation count or professional status since we believe the quality should be judged from the submission itself rather than the history of its author (which might have been influenced by previous biases).” I am sympathetic to this line of thinking, and don’t put too much credibility in studies that attempt to use such controls. However, using only variables endogeneous to the process just rearranges the dependent variable somewhat, it can’t explain the process. So, without any controls for quality, I think one has to be circumscribed in what can be concluded from the data, especially given that the most credible piece of prior work finds no bias (Forcher et al. 2019, https://www.nature.com/articles/s41562-018-0517-y)
- So what I recommend is
- Get rid of the 2 attempted controls, method 1b and method 2. I don’t think they help interpretation and axing them would help shorten the paper
AED> I take your point about method 1b being somewhat circular, and this was the longer control to discuss, so I have just mentioned that we considered it in the methodology and removed the bits in sections E1 and E2 which refer to it as a control and discussion of it as such in the conclusions. However, in G2, Figure 26, we think that citation success proportion versus whether the paper was unanimously accepted is interesting, so have kept that in but just not referred to it as a “control”. I have kept method 1a since it’s not too long and other readers might well have your concerns and it shows that we thought about it and explored it. It might also be useful to other people considering similar studies to give them ideas for controls but to let them know that it didn’t work for us.
- In the Conclusion section, get rid of “…but have investigated using single-authored submissions (unsuccessfully) and unanimous referee decisions (more successfully) as controls which are proxies for quality. In all these cases (with or without controls) there are consistently lower female proportions of successful submissions in all groups.” To a distracted reader (which is probably most us) the structure of this sentence makes it sound like some exogeneous information was brought to bear and it validated the interpretation of bias.
AED> I have reworded these sentences to be clearer and more qualified in the point that they are making.
- Add a small “disclaimer” sentence somewhere in the introduction, emphasizing that you are reporting gender gaps, but what explains these gaps is not entirely clear, and there is some debate in the literature. This would be a good place to cite the Forcher et al 2019 paper, unless there is some compelling reason not to (P.S. It’s not my paper)
AED> We have added that disclaimer as a penultimate paragraph of the introduction
- With all these comments I am pushing the authors to soften their conclusions, particularly for readers who may be unfamiliar with this literature. But I don’t think the value of the paper is much diminished. It’s still a very valuable analysis, and I look forward to seeing it in print.
AED> I have re-read the paper thoroughly and adjusted the terminology in it where appropriate

- P.S. Minor point: In my first review I took issue with a p-value on Page 12, Figure 14. I think it's now Figure 10 in the new draft. This may be a conventions issue across disciplines, but in my area, the syntax corr_author_gender ~ category*impact_factor usually means
- Corr_author_gender = Beta1*category + Beta2*impact_factor + Beta3*category*impact_factor + error.
- So if category is a categorical variable with many levels, there will be a separate intercept and separate slope for each one, and associated p-values with each. So it's not clear what the one reported p-value was referring to, perhaps the p-value for the overall model fit? If it's for the interactions, there should be a separate p-value for each interaction term (category-specific slope) and each intercept (categoryspecific-intercept). Perhaps I'm misunderstanding the regression specification; if the authors have a different one in mind, I would recommend noting that in the paper somewhere

AED> Apologies, it appears that some of the caption for this figure were truncated. These are the Pr(>Chi) values which come out of the ANOVA chisq test for the model corr_author_gender ~ category*impact_factor which we quote 3 values for – one for category, one for impact_factor and one for category:impact_factor. I've updated the caption (for that figure and similar ones) slightly so that hopefully this is clearer.

Round 3 – Decision Letter

13-Jan-2020

Dear Dr Day:

Manuscript ID: SC-EDG-08-2019-004090.R2
TITLE: Is there a gender gap in Chemical Sciences scholarly communication?

Thank you for submitting your revised manuscript to Chemical Science. After considering the changes you have made, I am pleased to accept your manuscript for publication in its current form. I have copied any final comments from the reviewer(s) below.

You will shortly receive a separate email from us requesting you to submit a licence to publish for your article, so that we can proceed with publication of your manuscript.

I would like to offer my congratulations on the acceptance of your manuscript. Please feel free to use the attached image should you wish to promote your upcoming Chemical Science article on social media.

Chemical Science welcomes cover artwork submissions. If you are interested in submitting cover artwork to highlight your article and the work of your group, please respond to this email within one week and note that all artwork submissions must be received within two weeks of acceptance.

As an author you are entitled to a 25% discount on books published by the Royal Society of Chemistry. To receive this discount, enter the promotional code JLTH25 when purchasing from our online bookshop (pubs.rsc.org/bookshop). Please contact booksales@rsc.org if you have any problems.

Discover more Royal Society of Chemistry author services and benefits here:

http://www.rsc.org/journals-books-databases/about-journals/benefits-of-publishing-with-us/

Thank you for publishing with Chemical Science, a journal published by the Royal Society of Chemistry – connecting the world of science to advance chemical knowledge for a better future.

With best wishes,

Alán Aspuru-Guzik
Professor of Chemistry and Computer Science
Faculty Member Vector Institute for Artificial Intelligence
University of Toronto

Associate Editor, Chemical Science
80 St George St, Toronto ON M5S 2H6 aspuru@utoronto.ca
